# Induction of Isochromanones by Co-Cultivation of the Marine Fungus *Cosmospora* sp. and the Phytopathogen *Magnaporthe* *oryzae*

**DOI:** 10.3390/ijms23020782

**Published:** 2022-01-11

**Authors:** Ernest Oppong-Danquah, Martina Blümel, Silvia Scarpato, Alfonso Mangoni, Deniz Tasdemir

**Affiliations:** 1 GEOMAR Centre for Marine Biotechnology (GEOMAR-Biotech), Research Unit Marine Natural Product Chemistry, GEOMAR Helmholtz Centre for Ocean Research Kiel, Am Kiel-Kanal 44, 24106 Kiel, Germany; eoppong-danquah@geomar.de (E.O.-D.); mbluemel@geomar.de (M.B.); 2 Dipartimento di Farmacia, Università degli Studi di Napoli Federico II, via Domenico Montesano 49, 80131 Napoli, Italy; silvia.scarpato@unina.it (S.S.); alfonso.mangoni@unina.it (A.M.); 3 Faculty of Mathematics and Natural Science, Kiel University, Christian-Albrechts-Platz 4, 24118 Kiel, Germany

**Keywords:** marine fungi, *Cosmospora* sp., soudanone, *Magnaporthe oryzae*, co-culture, phytopathogen, molecular networking, metabolomics

## Abstract

Microbial co-cultivation is a promising approach for the activation of biosynthetic gene clusters (BGCs) that remain transcriptionally silent under artificial culture conditions. As part of our project aiming at the discovery of marine-derived fungal agrochemicals, we previously used four phytopathogens as model competitors in the co-cultivation of 21 marine fungal strains. Based on comparative untargeted metabolomics analyses and anti-phytopathogenic activities of the co-cultures, we selected the co-culture of marine *Cosmospora* sp. with the phytopathogen *Magnaporthe oryzae* for in-depth chemical studies. UPLC-MS/MS-based molecular networking (MN) of the co-culture extract revealed an enhanced diversity of compounds in several molecular families, including isochromanones, specifically induced in the co-culture. Large scale co-cultivation of *Cosmospora* sp. and *M.* *oryzae* resulted in the isolation of five isochromanones from the whole co-culture extract, namely the known soudanones A, E, D (**1**-**3**) and their two new derivatives, soudanones H-I (**4**-**5**), the known isochromans, pseudoanguillosporins A and B (**6**, **7**), naphtho-*γ*-pyrones, cephalochromin and ustilaginoidin G (**8**, **9**), and ergosterol (**10**). Their structures were established by NMR, HR-ESIMS, FT-IR, electronic circular dichroism (ECD) spectroscopy, polarimetry ([α]_D_), and Mosher’s ester reaction. Bioactivity assays revealed antimicrobial activity of compounds **2** and **3** against the phytopathogens *M. oryzae* and *Phytophthora infestans*, while pseudoanguillosporin A (**6**) showed the broadest and strongest anti-phytopathogenic activity against *Pseudomonas syringae*, *Xanthomonas campestris*, *M. oryzae* and *P. infestans*. This is the first study assessing the anti-phytopathogenic activities of soudanones.

## 1. Introduction

Fungi are prolific producers of bioactive natural products that have found valuable applications in the agrochemical industry as pesticides and biocontrol agents [1]. There is an urgent need for new, natural agrochemicals against crop diseases, but the rediscovery of the known compounds poses a major hurdle to fungal natural product biodiscovery endeavours. Although recent fungal genomics have indexed huge numbers of biosynthetic gene clusters (BGCs), the majority of BGCs remain transcriptionally silent under standard laboratory conditions [2]. The cryptic BGCs encode the enzymatic machinery for the synthesis of a plethora of yet untapped secondary metabolites (SMs). Therefore, the application of culture-based approaches that awaken the silent BGCs promises the discovery of bioactive and novel compounds [3]. One of the effective strategies employed to enhance the expression of silent BGCs in fungal cultures is co-cultivation, which is based on the premise that two or more microorganisms growing within a confined environment respond to environmental cues, which trigger the activation of BGCs to produce, often new, bioactive SMs. Co-cultivation has already proven successful to yield new bioactive compounds or enhance the production of previously identified compounds.

Different strategies exist for designing co-cultivation experiments, such as the co-cultivation of microbes from the same habitat, or the co-cultivation of microorganisms unlikely to co-occur in the same habitat [4,5]. Phytopathogens are considered effective competitors in co-cultivation experiments as, in natural settings, they must overcome plant innate immunity, including beneficial epiphytes and endophytes prior to their colonization [6]. Upon contact with a host plant, the pathogen has to successfully outcompete host symbionts, regarding space and nutrients, which is usually achieved by direct antagonism [7]. Consequently, phytopathogens lend themselves as useful models to investigate competitive microbial interactions.

In a previous study, we obtained 123 marine fungal isolates belonging to 30 genera from the Baltic Sea [4]. Based on phylogenetic and metabolic diversity, 21 fungal isolates were selected and co-cultured with two bacterial (*Pseudomonas syringae* and *Ralstonia solanacearum*) and two fungal (*Magnaporthe oryzae* and *Botrytis cinerea*) phytopathogens on two different solid media, Saboroud Agar (SA) and Potato Dextrose Agar (PDA). Comparative metabolomics of the crude organic extracts of co-cultures and the axenic cultures using HRMS/MS-based molecular networking (MN) and in vitro anti-phytopathogenic activity assays led to the prioritization of the co-culture of *Cosmospora* sp. and *M. oryzae* in the PDA medium. This co-culture was selected for in-depth chemical analysis because of (i) its unique chemical diversity, (ii) its clear induction of several compounds in the co-culture, and (iii) its antimicrobial activity against numerous phytopathogens [4].

Herein, we have investigated the chemical composition of the upscaled co-cultures of *Cosmospora* and *M. oryzae* and their axenic-cultures grown on a PDA medium by MN-based metabolomics to observe the specific induction of the isochromanone type SMs, which were absent in the mono-cultures. Chromatographic separation of the CH_2_Cl_2_-soluble portion of the co-cultures led to the isolation of two new isochromanones, soudanones H-I (**4**-**5**), and the known soudanones A, E, D (**1**-**3**), together with the known isochromans, pseudoanguillosporins A and B (**6**, **7**), the known naphtho-*γ*-pyrones, cephalochromin and ustilaginoidin G (**8**, **9**), and ergosterol (**10**). Herein, we report on the in-depth metabolomics analyses, followed by the isolation and structure elucidation of the induced isochromanones (**1**-**5**) and the known compounds (**6**-**10**), as well as their anti-phytopathogenic activities.

## 2. Results

### 2.1. Co-Culture and Description of MN and Annotations

**Figure 1:**
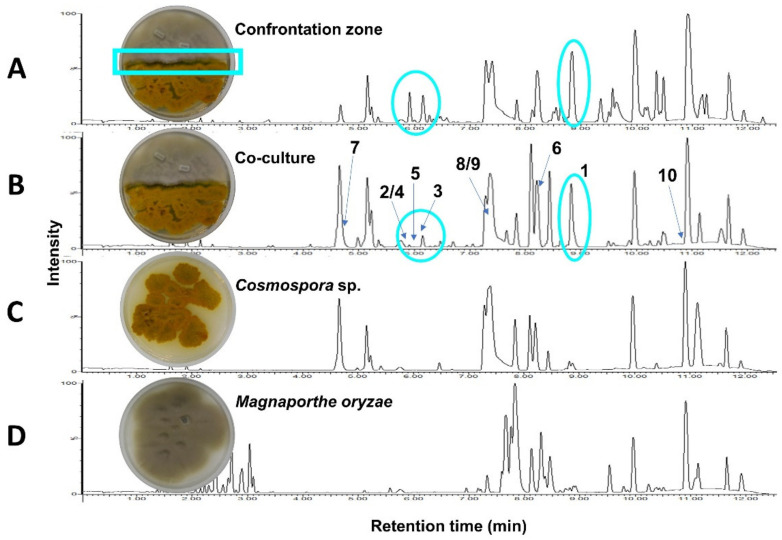
UPLC-MS chromatograms of the extracts obtained from (**A**) the confrontation zone (blue rectangle) excised from (**B**) the whole co-culture of *Cosmospora* sp. and *Magnaporthe oryzae*, and the monocultures of (**C**) *Cosmospora* sp., and (**D**) *M. oryzae*. Peak ions corresponding to compounds **1–5** (highlighted in blue) were induced in the whole co-culture (**B**) but of low intensities. They were however observed in higher intensities in the confrontation zone (**A**) (also highlighted in oval blue).

A UPLC chromatogram of the crude EtOAc extract of a 21-day PDA whole co-culture of *Cosmospora* sp. and *M. oryzae* revealed the induction of the compounds **1**-**5** (Figure 1B), which were absent in the monocultures (Figure 1C,D). These metabolites were accumulated in the confrontation zone (Figure 1A), i.e., at the site of direct interaction between two fungi. We hypothesized that compounds **1**-**5** were produced for competitive advantage and so they were prioritized for isolation from the whole co-cultures.

**Figure 2:**
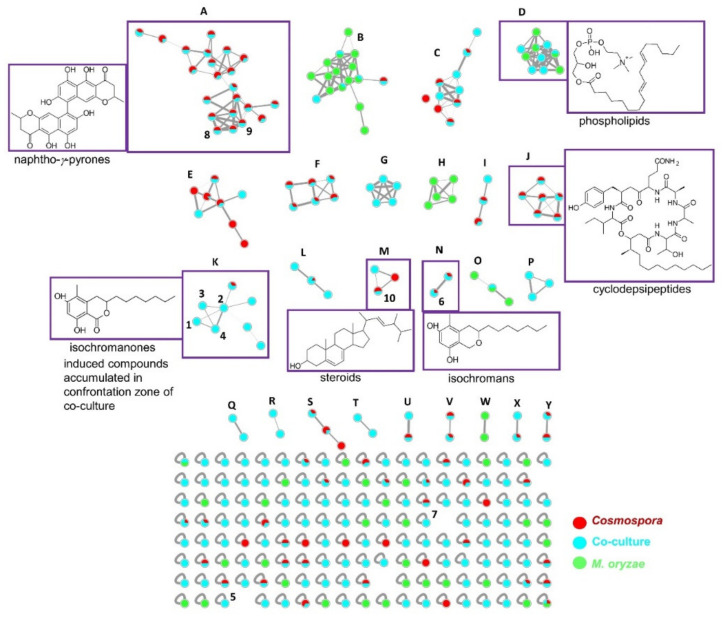
Molecular network (MN) of *Cosmospora* sp. (red), *M. oryzae* (green) and their co-culture (blue). Dereplicated clusters (A-Y) and representative structures are highlighted in purple next to each other. Compounds **1**-**4** are the induced isochromanones in the co-culture, while compounds **5** and **7** were displayed as singletons in the MN. Some nodes in clusters represent isotopic nodes with a mass difference of +1 Da from the neighbouring node.

In order to investigate the metabolome and to obtain more information on the induced compounds (**1**-**5**), a UPLC-QToF-MS/MS based MN was generated with the crude extracts of the fungal co-culture and the corresponding axenic cultures (Figure 2). The MN analysis revealed that co-cultivation generally increased the chemical space of the fungi, indicated by a size increase of several clusters with co-culture induced compounds (blue only nodes), such as clusters C, D, E, I, K, L and M. Moreover, we observed five clusters (G, P, Q, R and T) that were exclusively induced in the co-cultures. The latter clusters remained unidentified as they showed no match to known compounds in several commercial and public databases. Unfortunately, no ion belonging to these clusters could be purified in sufficient quantity to allow chemical identification.

The biggest cluster, A, shared by the *Cosmospora* sp. and the co-culture extracts, was annotated to the naphtho-*γ*-pyrone class of compounds, which included ustilaginoidins A (*m/z* 515.1259 [M+H]^+^), D (*m/z* 547.1599 [M+H]^+^), E (*m/z* 533.1479 [M+H]^+^) and V (*m/z* 535.1237 [M+H]^+^) (Figure 2 and Supplementary Table S1) [8,9]. Two additional nodes (compounds), **8** (*m/z* 519.1284 [M+H]^+^) and **9** (*m/z* 517.1150 [M+H]^+^), belonging to this cluster were produced in significant amounts. They were purified and identified as the known naphtho-*γ*-pyrones, cephalochromin (**8**) and ustilaginoidin G (**9**), to confirm the cluster annotation.

Another small cluster annotation confirmed by purification was the isochroman class of compounds (cluster N). It contained two nodes originating from both *Cosmospora* sp. mono-culture and the co-culture extracts. One node, *m/z* 279.1939 [M+H]^+^, was purified and identified as the known isochroman, pseudoanguillosporin A (**6**) [10]. The second node, *m/z* 279.0970 [M+H]^+^, could not be assigned to any known compound in databases, so remains unidentified. Another node (singleton), *m/z* 295.1912 [M+H]^+^, had a mass difference of 16 Da from **6**, being indicative of an additional oxygen atom. It was identified as the known isochroman, pseudoanguillosporin B (**7**), that bears a hydroxy substitution on the alkyl side chain [10]. Pseudoanguillosporin B did not cluster with the isochromans in cluster N because the product ions displayed a similarity score (cosine score) of <0.7, as estimated from the MN algorithm (refer to Section 4.4).

Cluster M was identified as a steroid family, based on the purification and identification of **10** as ergosterol *m/z* 419.3290 [M+Na]^+^ [11,12]. Other putative annotations, based on manual dereplication, include the cyclodepsipeptides acuminatums B (*m/z* 888.5449 [M+H]^+^) and C (*m/z* 874.5460 [M+H]^+^) [13] in cluster J (nodes shared between *Cosmospora* sp. and co-culture), as well as a phospholipid (*m/z* 520.3740 [M+H]^+^) [14] in cluster D, which was shared by *M. oryzae* and the co-culture. All annotations are listed in Table S1.

**Figure 3:**
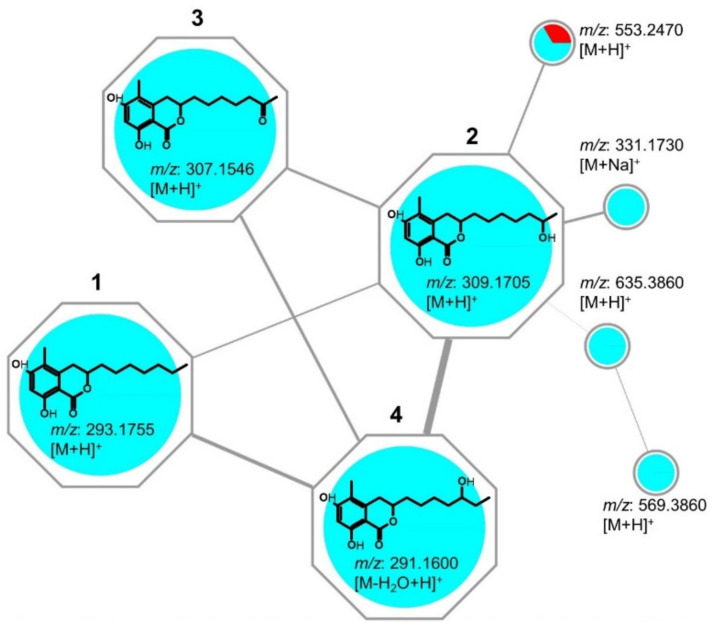
Close-up of molecular family cluster K containing the known compounds (**1**, **2** and **3**) induced in the co-culture (and significantly observed in the confrontation zone). Likewise, the new compound **4** and the other nodes were induced in the co-culture, except *m/z* 553.2470 [M+H]^+^, which was also produced in the *Cosmospora* mono-culture. Nodes coloured blue originate from the co-culture, and red from *Cosmospora* monoculture. The compound **5** is observed as a singleton (Figure 2).

MN identified the induced compounds **1**-**5** as belonging to a common molecular family, K (Figure 2), the isochromanones. The cluster K contained 8 nodes (Figure 3), of which 7 were exclusively observed in the co-culture. The first node, (**1**) *m/z* 293.1755 [M+H]^+^, was isolated and identified as the known isochromanone, soudanone A. Another node, (**2**) *m/z* 309.1705 [M+H]^+^, showed a mass difference of 16 Da from **1**, indicative of hydroxylation, and was identified as soudanone E. The sodiated adduct of **2** (*m/z* 331.1730 [M+Na]^+^) was also observed. As indicated in the experimental section, the clusters were generated with a defined cosine score (similarity index) above 0.7 and the edges were modulated accordingly. The thick edge between nodes **2** and **4** indicates high structural similarity. As expected, these nodes shared highly similar MS/MS spectra, thus node **4** was predicted to be a positional isomer of **2,** the new soudanone H, visualized in the network as a dehydrated adduct (*m/z* 291.1600 [M-H_2_O+H]^+^. Node (**3**), *m/z* 307.1546 [M + H]^+^, had a mass difference of 2 Da from nodes **2** and **4**, which suggested the oxidation of a hydroxyl group to a ketone. Node **3** was identified as the known soudanone D by isolation. Other observed nodes in this cluster (K) were *m/z* 635.3860 [M+H]^+^, *m/z* 569.3860 [M+H]^+^, and *m/z* 553.2470 [M+H]^+^. Unfortunately, these nodes (compounds) representing potentially new isochromanones remain unidentified because they could not be purified in sufficient quantities. Notably, all nodes in this cluster originated from the co-culture (blue nodes) except *m/z* 553.2470 [M+H]^+^, which was also detected in the *Cosmospora* sp. monoculture (red node, Figure 3). In addition, another new isochromanone, (**5**) *m/z* 309.1341 [M + H]^+^, soudanone I, was identified as a single node in the MN (Figure 2). It did not cluster with the isochromanone molecular family, because it displayed slightly different product ions due to the shorter side chain and the terminal esterification. All identifications and annotations are listed in Table S1.

### 2.2. Isolation & Structure Elucidation

**Figure 4:**
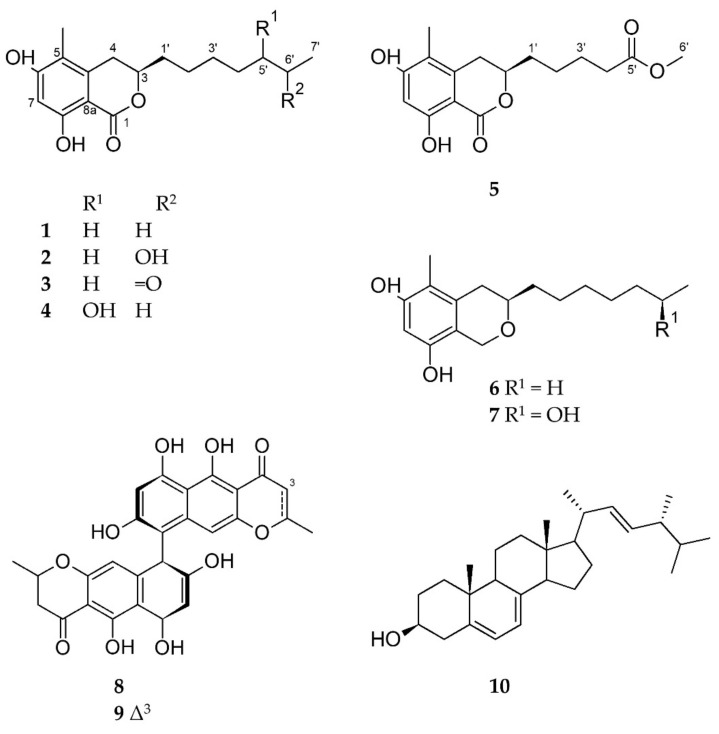
Chemical structures of compounds **1–10.**

For isolation of the compounds, the crude EtOAc extract obtained from scaled-up solid co-cultures (100 PDA plates) of *Cosmospora* sp. and *M. oryzae* was subjected to a modified Kupchan solvent partitioning scheme [15] to yield *n*-hexane, CH_2_Cl_2_ and aqueous MeOH subextracts. Only the CH_2_Cl_2_ subextract exhibited activity against the phytopathogens *X. campestris*, *P. infestans* and *M. oryzae* (Table S2), and contained the prioritized isochromanones (**1**-**5**). The CH_2_Cl_2_ subextract was selected for further work-up and fractionated over a C18 SPE cartridge, followed by repeated RP-HPLC separations to afford the co-culture induced compounds **1**-**5**, as well as compounds **6**-**10** (Figure 4).

**Figure 5:**
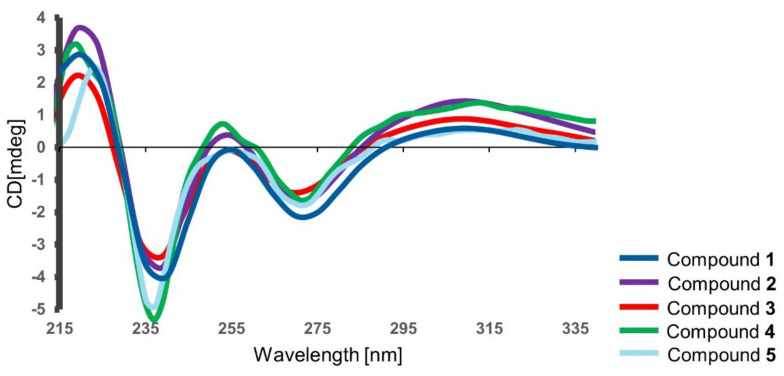
Experimental electronic circular dichroism (ECD) spectra of compounds **1** (dark blue), **2** (purple) and **3** (red), **4** (green) and **5** (light blue) in MeOH.

Compound **1** was obtained as a yellowish amorphous film with the molecular formula C_17_H_25_O_4_ deduced by HR-ESIMS (*m/z* 293.1755 ([M+H]^+^). 1D and 2D NMR data analyses (Table 1 and Table 2, Figures S1–S10), as well as the FT-IR data (Figure S11), led to the identification of **1** as soudanone A, an isochromanone previously reported from a *Cadophora* sp. isolated from the Soudan Underground Mine [16]. Compound **1** exhibited the same sign and a similar magnitude of specific rotation value (${\left[\alpha \right]}_{D}^{20}$-59, *c* 0.5, MeOH) as soudanone A (${\left[\alpha \right]}_{D}^{23}$-43, *c* 0.065, MeOH) [16]. In addition, the experimental electronic circular dichroism (ECD) spectrum of **1** (Figure 5) was similar to that of soudanone A, in that it possessed an *R* configuration at C-3 [16]. Hence, compound **1** was unambiguously identified as soudanone A.

Compound **2** was assigned the molecular formula C_17_H_25_O_5_, deduced by HR-ESIMS (*m/z* 309.1705 ([M+H]^+^, Figure S18). With the knowledge of compound **2** and **1** clustering in the MS/MS molecular network, compound **2** was assigned as an oxygenated derivative of **1** with an increase of 16 mass units. The HRMS, FT-IR, ^1^H and ^13^C NMR, COSY, HMBC and NOESY spectra (Table 1 and Table 2, Figures S12–S20) were consistent with the known soudanone E, that contains an OH group attached at a C-6’ position on the alkyl chain [16]. The optical rotation of **2** (${\left[\alpha \right]}_{D}^{20}$-50, *c* 0.05, MeOH) and soudanone E $({\left[\alpha \right]}_{D}^{25}$-40, *c* 0.065, MeOH) were comparable in sign and magnitude. The measured ECD spectrum of **2** was similar to **1** (Figure 5) and not affected by the presence of a second chiral centre at C-6’. Hence, we assigned the absolute configuration of C-3 as *R*. Next, we attempted to determine the configuration at C-6’, as yet uncharacterized in the literature [16], by comparing the NMR data of the side chain of **2** to those published for pseudoanguillosporin B (**7**) [10]. Pseudoanguillosporin B (**7**), a highly related isochroman type of molecule (also isolated in this study), contains a hydroxyl function at a C-6’ position with *R* configuration, determined by Mosher’s NMR method [10]. As shown in Table S3, compounds **2** and **7** showed almost identical ^1^H/^13^C NMR signals for positions in the side chain, particularly for H-6’/C-6’ and H_3_-7’/C-7’, suggesting an *R* configuration for C-6’. To confirm this assignment, we performed Mosher’s ester derivatization. Due to the very small amount of **2** available, we chose a nanomole-scale protocol using methoxyphenylacetic (MPA) acid as the chiral derivatizing agent [17]. The phenolic hydroxy groups were protected by reacting with (trimethylsilyl)diazomethane to give a dimethyl ether derivative, then the (*R*)-MPA and (*S*)-MPA esters were prepared (Figure S50). LC-MS analyses of the resulting reaction mixtures revealed compound **2** to be a mixture of two epimers at C-6’ in the 2:1 ratio (Figure S51; details can be found in the Supporting Information section). 1D-TOCSY experiments recorded on the crude reaction mixtures (Figure S52) were used to assign the ^1^H NMR signals of the MPA esters. Due to the small amounts of esters obtained, only the chemical shift of the terminal methyl (H_3_-7’) could be determined, but this was sufficient to assign configuration. For the most abundant epimer, H_3_-7’ was deshielded in the (*R*)-MPA ester and shielded in the (*S*)-MPA ester. This indicated the 6’*R* configuration for the major epimer. Therefore, compound **2** was identified as an inseparable 2:1 mixture of (3*R*,6’*R*)-soudanone E and (3*R*,6’*S*)-soudanone E. It is worth noting that neither ^1^H nor ^13^C NMR provided any clue that compound **2** was in fact a mixture of diastereomers. This means that the spectra of the two diastereomers are identical, and indeed several examples have been reported where two diastereomers with chiral centers far from each other show indistinguishable NMR spectra [18].

Compound **3** was purified as a yellow amorphous solid with a molecular formula of C_17_H_23_O_5_ on the basis of its HR-ESIMS data (*m/z* 307.1546 ([M+H]^+^). Analysis of the 1D and 2D NMR data (Table 2, Figures S21–S29, including the FT-IR spectrum), as well as the comparison of its specific rotation value (${\left[\alpha \right]}_{D}^{20}$-56, *c* 0.35, MeOH) with that of soudanone D (${\left[\alpha \right]}_{D}^{23}$-40, *c* 0.05, MeOH), led to the identification of compound **3** as soudanone D [16]. Compound **3** also displayed a similar ECD spectrum (Figure 5) as **1**, hence was unambiguously identified as (3*R*)-soudanone D.

**Figure 6:**
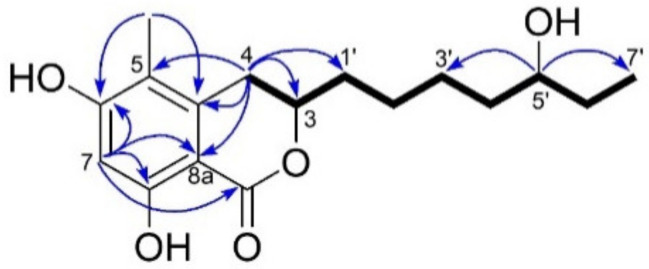
Key COSY (bold lines) and HMBC (blue arrows) correlations observed for compound **4.**

Compound **4** was assigned the same molecular formula as compound **2**, C_17_H_25_O_5_, deduced by HR-ESIMS (*m/z* 309.1703 ([M+H]^+^, Figure S36). This indicated 6 degrees of unsaturation. The FT-IR absorption bands, observed at 3390 and 1646 cm^−1^, indicated the presence of hydroxyl and carbonyl groups, respectively (Figure S38). The ^13^C NMR spectrum contained 17 carbon resonances corroborating the predicted molecular formula from the HR-ESIMS. These included a carbonyl (*δ*_C_ 170.5), six olefinic carbons (*δ*_C_ 139.5, 112.9, 160.5, 101.6, 162.5 and 102.2), two oxymethine carbons (*δ*_C_ 78.5, 73.3) and two methyl groups (*δ*_C_ 10.0 and 10.6). ^1^H NMR and HSQC spectra revealed an aromatic proton (*δ*_H_ 6.29 s, H-7), two oxymethine protons (*δ*_H_ 4.47 m, *δ*_H_ 3.54 m), an olefinic methyl (*δ*_H_ 2.07 s, H_3_-4), a primary methyl group (*δ*_H_ 0.95 t, *J* = 7.5 Hz), and six aliphatic methylene protons between *δ*_H_ 1.26-2.94 (Table 1 and Table 2**, Figures S30–S31**, Figure S33). This data was suggestive of the same isochromanone core structure found in compounds **1**-**3**, supported by whole set of 2D NMR experiments (Figures S32–S35). The COSY spectrum revealed only one large spin system (Figure 6 and Figure S32). It started from the diastereotopic methylene protons at *δ*_H_ 2.71 and 2.94 (H_2_-4) that coupled with the oxymethine proton at *δ*_H_ 4.47 (m, H-3), which in turn coupled to a methylene proton (*δ*_H_ 1.75 and 1.90, H_2_-1’). The spin network correlations further incorporated four methylenes (H_2_-2’-H_2_-6’), another oxymethine proton (*δ*_H_ 3.54 m, H-5’) and a terminal methyl group (*δ*_H_ 0.95 t, *J* = 7.5 Hz, H_3_-7’), resulting in an unbranched aliphatic chain similar to that observed in **2.** The major difference between **2** and **4** was represented by the attachment of the secondary OH function at C-5′ in **4**, instead of C-6′ in **2** (Figure 6). This assumption was verified on the basis of COSY correlations observed between H-5’/H-6’ and H-5’/H-4’, as well as by HMBC correlations between H-5’/C-7’and H-5’/C-3’ (Figure 6)**.** Thus, the side chain of **4** was confirmed as heptan-3-ol. The measured ECD spectrum of **4** was similar to that of **2** (Figure 5) and their optical rotation values were almost identical (**2** ${\left[\alpha \right]}_{D}^{20}$-50, *c* 0.05, MeOH; **4** ${\left[\alpha \right]}_{D}^{20}$-44, *c* 0.05, MeOH). The configuration of C-3 in **4** was thus deduced as (*R)*. The absolute configuration of C-5’ was investigated by Mosher’s ester method mentioned above. Due to the small amounts of sample, only the (*R*)-MPA ester was prepared (Figure S53), with the intention of using the variable-temperature NMR measurement to assign absolute configuration at C-5’ [17]. However, similar to compound **2**, compound **4** was determined to be a mixture of two epimers at C-5’. Based on the observation of two peaks of nearly equal intensity in the LC-MS chromatogram of the (*R*)-MPA ester (Figure S54), in this case the epimers were determined to be in the 1:1 ratio. This made it unnecessary to use NMR to assign the configuration at C-5’. Therefore, compound **4** was identified as an inseparable 1:1 mixture of epimers, for which we propose the trivial names of (3*R,* 5’*R*)-soudanone H and (3*R,* 5’*S*)-soudanone H.

Compound **5** was assigned the molecular formula C_16_H_21_O_6_ based on the *m/z* 309.1341 ([M+H]^+^) observed in the HR-ESIMS spectrum (Figure S45), indicating 7 degrees of unsaturation. In-depth analysis of the 1D and 2D NMR, and the FT-IR data of **5** (Figures S39–S47) provided evidence for the same isochromanone structure with a carbonyl function on the alkyl chain, as in **3**. The main differences between compounds **5** and **3** rested in the absence of one methylene group (H_2_-5’) in the proton spin system of the side chain of **5,** and the replacement of the methyl ketone terminal group with a carboxymethyl ester attached at C-4’. Due to the ester formation, H_2_-4’ was shifted to downfield and appeared as a triplet at *δ*_H_ 2.36 (*J* = 7.3 Hz). The presence of the carboxymethyl ester terminal in **5** was evident from the ^1^NMR resonances of C-5’ (*δ*_C_ 174.1 s) and CH_3_-6’ (*δ*_H_ 3.68 s; *δ*_C_ 51.7 q). COSY correlations on the side chain from H_2_-1’ through H_2_-4’ (Figure S41) and diagnostic HMBC correlations between H_3_-6’/C-5’ and H_2_-4’/C-5’ (Figure S43) confirmed that methyl pentanoate was the side chain of **5**. The measured ECD spectrum (Figure 5) and the optical rotation value of **5** ${\left[\alpha \right]}_{D}^{20}$-20 (*c* 0.07, CH_3_OH) were similar to compounds **1**-**4**, hence the configuration at C-3 was also proposed as (*R*). We propose the trivial name (3*R*)-soudanone I for compound **5**.

In addition to the five soudanones (**1**-**5**) described above, two known isochromans were isolated and identified as pseudoanguillosporins A (**6**) and B (**7**) by comparing their 1D NMR, HR-ESIMS and [α]_D_ data with those reported in the literature [10]. The other isolates **8**-**9** were identified as bis-naphtho-*γ*-pyrone type mycotoxins, cephalochromin (**8**) [10] and ustilaginoidin G (**9**) [8,19], while the compound **10** was characterized as ergosterol [11], based on their NMR, HR-ESIMS and [α]_D_ data.

### 2.3. Bioactivity of Pure Compounds

The inhibitory activities of the isolated compounds were tested against a panel of plant pathogens (Table 3). The new soudanones H and I (**4** and **5**) were not tested because they were obtained in very low amounts. Soudanone A (**1**) and ergosterol (**10**) were inactive against all phytopathogens, even at the highest test concentration (100 µg/mL), while soudanones E and D (**2** and **3**) exerted moderate activities against the oomycete *P. infestans*, the fungus *M. oryzae* and the bacterium *X. campestris* with IC_50_ values between 12.8 and 71.5 µg/mL. Pseudoanguillosporin A (**6**) exhibited the strongest (IC_50_ values 0.8–23.4 µg/mL) and broadest activity against all tested phytopathogens, except *E. amylovora*, which was not susceptible to any of the compounds. Cephalochromin (**8**) inhibited *P. infestans*, *R. solanacearum* and *X. campestris* (IC_50_ values 2.3, 27.6 and 12.1 µg/mL, respectively). Ustilaginoidin G (**9**) exerted moderate inhibition against *P. infestans* (IC_50_ value 7.2 µg/mL) and *X. campestris* (IC_50_ value 21.7 µg/mL).

### 2.4. Optimization Study to Enhance Production of the Soudanones

We intended to optimize the fermentation conditions to increase the production of the isochromanones to allow for further biological assays with pure compounds. Considering that the isochromanones were accumulated in the confrontation zone, i.e., the site of interaction between both co-cultivation partners, we tried co-cultivation by overlaying *Cosmospora* over pre-grown *M. oryzae* to examine if an enhanced contact area increases the production of the isochromanones (**1**-**5**). All five isochromanones were produced and observed in the resulting EtOAc crude extract (Figure S48). With the exception of **1**, whose production was evident from the UPLC-MS base peak chromatogram (Figure S48), soudanones **2**-**5** were still produced in small amounts (assessed by small peaks in UPLC-MS base peak chromatogram, Figure S48). In the next step, *Cosmospora* sp. and *M. oryzae* were co-cultured in a liquid culture regime using a potato dextrose broth (PDB) medium under aeration, which facilitates contact of competing strains and ensures constant oxygenation. However, co-cultivation in broth did not yield any of the isochromanones **1**-**5** (Figure S49). On the other hand, cephalochromin (**8**) and ustilaginoidin G (**9**) were already observed on day 3 of broth co-cultivation, peaking on day 6, with sustained levels of production as major peaks across all 21 days of cultivation (Figure S49). This result underscores the high complexity and unpredictability of fungal interactions. Although these approaches failed to increase further yields of **1**-**5**, other fermentation conditions, such as cultivation time, temperature, pH and media, may be further exploited to optimize the production of the isochromanones to enable further testing against a more diverse panel of pathogens.

## 3. Discussion

Enhancement of the chemical diversity of fungal metabolomes through co-cultivation is becoming a promising strategy for the discovery of new bioactive natural products. Recently, pathogens are emerging as effective model competitors to trigger or enhance the production of anti-pathogenic compounds (antibiotics) in microbial species which do not naturally co-exist with the pathogens. For example, Serrano et al. [5] showed the increased diversity of antifungal compounds following the interaction of the plant pathogen *B. cinerea* with a large panel of fungal strains isolated from different environmental niches, such as soils, leaf litters and rhizosphere. Several new molecules, identified by LC-MS, were induced in the co-cultures, supporting the hypothesis that co-cultivation with phytopathogens enhances chemical diversity [5]. Not only phytopathogens, but also human pathogens have been investigated as good competitors in co-cultivation experiments. The production of the antibiotics granaticin, granatomycin D and dihydrogranaticin B showed a multiple-fold increase when the tunicate derived actinomycete *Streptomyces* sp. was co-cultured with human pathogens, such as *Bacillus subtilis* and methicillin-resistant *Staphylococcus aureus* (MRSA) [20].

In our previous study, we demonstrated that the use of economically-relevant phytopathogens as challengers in co-cultivation experiments can increase the chemical space of marine-derived fungi, since over 11% of all compounds detected exclusively originated from the co-cultures [4]. In addition, the MN-based metabolomics and anti-phytopathogenic bioactivity screening resulted in the prioritization of the co-culture of the marine fungus *Cosmospora* sp. and one of the most devastating rice pathogens *M. oryzae* in the PDA medium [4]. In the present study, large scale co-culture fermentation of *Cosmospora* and *M. oryzae* was conducted for natural product isolation. Five isochromanones (**1–5**) were biosynthesized only in the co-culture of *Cosmospora* sp. and *M. oryzae*, while the closely related isochromans, pseudoanguillosporins A (**6**) and B (**7**), and the naphtho-*γ*-pyrones, cephalochromin (**8**) and ustilaginoidin G (**9**), were detected also in the *Cosmospora* monoculture. Comparative metabolomics of the mono- and co-cultures by MN revealed the overall enhancement of chemical diversity in the co-culture. Five unannotated clusters (G, P, Q, R and T) were exclusively induced in the co-culture, whereas other clusters, such as the isochromanones (cluster K), increased in size by induction of new compounds.

Isochromanones are a family of benzopyranones widely distributed in nature. They represent an important family of secondary metabolites from plants and fungi [21,22] and display diverse biological activities, such as antibacterial, antiparasitic, anticancer, herbicidal and fungicidal [23,24]. Hence, it is reasonable to assume that the biosynthesis of isochromanones was induced in response to the fungal competition for the benefit of the producer fungus, as hypothesized [25]. To this end, the potential benefits of the induced compounds in the co-culture and constitutive compounds originating from the axenic cultures were assessed by their antimicrobial activities against a panel of phytopathogens. The isochromanones **2** and **3** showed varying degrees of inhibition against the test phytopathogens. Soudanone E (**2**), that contains an OH group at C-6’ of the side chain, exhibited the highest activity against the oomycete *P. infestans*, the bacterium *X. campestris*, and the competing phytopathogen, *M. oryzae* (IC_50_ values 27.6, 71.6 and 12.8 µg/mL, respectively). Soudanone D (**3**), bearing a keto function at the same position of the alkyl chain, showed only weak activity against *P. infestans* and *M. oryzae* (IC_50_ values 52.1 and 60.3 µg/mL respectively), while soudanone A (**1**), with no oxygenation on the side chain, was devoid of any activity against the test pathogens. Thus, the hydroxylation on the aliphatic side chain, (at position C-6’) seems to be a structural feature required for the anti-phytopathogenic activity of the isochromanones. Unfortunately, we were unable to assess the bioactivity of the new soudanones H-I (**4** and **5**), due to the minor amounts isolated, to provide additional insights into the structure-activity relationships of soudanones.

The highly related isochromans, pseudoanguillosporins A (**6**) and B (**7**), with the same type side chain displayed a more potent and broader range of activities against the phytopathogenic bacteria compared to the isochromanones. Pseudoanguillosporin A (**6**), that lacks oxygenation on the side chain, exhibited overall the most potent anti-phytopathogenic activity and its inhibitory potential against *M. oryzae* (IC_50_ value 0.8 3.2 µg/mL) was comparable to that of the positive control nystatin (IC_50_ value 0.4 µg/mL). Such biological activities were also reported by Kock et al. [10] where percentage-growth inhibition was evaluated. Interestingly, pseudoanguillosporin B (**7**), that bears an OH group at C-6’, was inactive towards *M. oryzae* and *P. infestans* at 100 µg/mL (Table 3), representing an opposite trend for the anti-phytopathogenic activity of isochromans. Pseudoanguillosporin B (**7**) showed low activity against the phytopathogenic bacteria *X. campestris* and *R. solanacearum.* The naphtho-*γ*-pyrones, cephalochromin (**8**) and ustilaginoidin G (**9**), moderately inhibited the growth of *X. campestris*. This aligns with a previous study, in which cephalochromin (**8**) was shown selectively to inhibit the bacterial enoyl-acyl carrier protein, reductase FabI (a highly conserved enzyme from the type II fatty acid biosynthesis of many pathogens) in *S. aureus* and *E. coli* [26]. Anti-phytopathogenic activities were also reported for ustilaginoidin G (**9**) against *X. vesicatoria* (a bacterial spot of tomatoes) and *Agrobacterium tumefaciens* (a crown gall disease of several plants) [27]. Cephalochromin is biosynthesised by several fungal genera, such as *Cephalosporium*, *Pseudoanguillospora* and *Plenodomus* [28,29]. Interestingly, its production in *Plenodomus influorescens* (Dothideomycetes, Pleosporales) was notably enhanced upon interaction with a *Pyrenochaeta* sp. (Dothideomycetes, Pleosporales), both isolated from sediment [29]. Based on the bioactivities of the isolated functional metabolites, naphtho-*γ*-pyrones, isochromans and the induced isochromanones appear to inhibit the growth of several devastating crop pathogens.

As examined in our previous study, the co-cultures of *Cosmospora* and the other three plant pathogens (*B. cinerea*, *P. syringae* and *R. solanacearum*) did not induce the production of the isochromanones [4], except in their co-cultivation with *M. oryzae*. Therefore, the producer fungus could not be definitively identified. However, several lines of evidence support our hypothesis that the isochromanones **1–5** are produced by *Cosmospora* sp. Firstly, the isochromans, pseudoanguillosporins A (**6**) and B (**7**), produced in a *Cosmospora* monoculture, share many structural similarities with the isochromanones; both the heterocyclic ring and aliphatic side chains. Secondly, the molecular family cluster of the isochromanones (Figure 3) displayed one shared node between both the co-culture and the *Cosmospora* monoculture. Therefore, it is inferred that the isochromanones (soudanones) are likely to be expressed by *Cosmospora* sp. when triggered by *M. oryzae*. More so, it is reasonable to assume that *M. oryzae* would not produce antibiotics inhibiting its own growth in co-cultures without self-protection mechanisms such as detoxification [30]. It is, however, worth mentioning that *Pyricularia grisea* (in the same monophyletic clade as *M. oryzae* [31]) produced 6-hydroxymellein and 3-methoxy-6,8-dihydroxy-3-methyl-3,4-dihydroisocoumarin, which possess a similar isochromanone core structure to the soudanones [32]. Further work on genome mining would be necessary to confirm the real producer of the induced isochromanones.

*Cosmospora* is a teleomorph genus in the family Nectriaceae (Ascomycetes). Its asexual morphs are mostly affiliated to the genera *Acremonium* and *Fusarium*, which are well-studied for fungal secondary metabolites [33,34]. Despite their close relationship with well-studied genera, there is little insight into the biosynthetic machinery and secondary metabolite production in the genus *Cosmospora*, supporting the relevance of this study. Only five classes of compounds have been reported from *Cosmospora* spp.: sesquiterpene glycoside cosmosporasides with reported nitric oxide production-inhibitory effects in lipopolysaccharide-activated murine macrophage RAW264.7 cells [35]; isoxazolidinone, containing parnafungins with antifungal activity [36,37]; orcinol *p*-depside acquastatins with enzyme inhibitory activity against protein tyrosine phosphatase 1B [38]; naphtho-*γ*-pyrone cephalochromin with anticancer activity [28]; and the dichlororesorcinol cosmochlorins that increase osteoclast formation in RAW264.7 cells [39]. The current study is the first report on the production of the isochromans, pseudoanguillosporins, and soudanone type isochromanones from *Cosmospora* sp. Pseudoanguillosporins were previously isolated from *Pseudoanguillospora* sp and *Cadophora* sp. with anti-phytopathogenic and antifungal activities [10,16]. Currently, pseudoanguillosporin A is isolated from *Strobilurus* sp. commercially and marketed as an antifungal and a fungal mitochondrial inhibitor (https://adipogen.com, accessed on 01 December 2021). The soudanones were first characterized as antifungal agents produced in the axenic cultures of another Ascomycete *Cadophora* sp. [16]. Our study is the second report on the production of soudanones by fungi.

Although the optimization study was not entirely successful, the observation of the induced isochromanones in the overlaid co-cultivation on solid agar (Figure S48) and their accumulation in the confrontation zone (Figure 1), suggested that direct cell-to-cell contact between *Cosmospora* and *M. oryzae* on a PDA medium may be crucial for their production. Schroeckh et al. [40] demonstrated a similar phenomenon: only cell-to-cell contact between the fungus *Aspergillus nidulans* and the bacterium *Streptomyces rapamycinicus* resulted in a specific activation of a PKS gene to produce orsellinic acid and the depside lecanoric acid. Our data suggest a similar phenomenon and warrants further experiments to validate the hypothesis.

## 4. Materials and Methods

### 4.1. General Experimental Procedures

Optical rotations were measured with a monochromatic light source in MeOH at 20 °C on a Jasco P-2000 polarimeter (Jasco, Pfungstadt, Germany). FT-IR spectra were recorded on a PerkinElmer Spectrum Two FT-IR spectrometer (PerkinElmer, Boston, MA, USA). The 1D (^1^H and ^13^C) and 2D (COSY, HSQC, HMBC, NOESY and TOCSY) NMR spectra were obtained on a BRUKER AV 600 spectrometer (600 and 150 MHz for ^1^H and ^13^C NMR, respectively, Bruker^®^, Billerica, MA, USA) or a Bruker Avance III spectrometer (500 MHz for ^1^H NMR, Bruker^®^, Billerica, MA, USA). All spectra were acquired in solvents as specified in the text with referencing to residual ^1^H and ^13^C signals in the deuterated solvent. HRMS/MS data were recorded on a Xevo G2-XS QToF Mass Spectrometer (Waters^®^, Milford, MA, USA) connected to an Acquity UPLC I-Class System (Waters^®^, Milford, MA, USA). Crude extracts were fractionated using Chromabond SPE C18 column cartridges (Macherey-Nagel, Düren, Germany). TLC analysis was done on silica gel 60 F254 plates (pre-coated aluminium sheets) cut into 10 × 10 cm (Macherey-Nagel, Düren, Germany). Semipreparative and Preparative HPLCs were performed using a VWR Hitachi Chromaster system (VWR International, Allison Park, PA, USA) consisting of a 5310 column oven, a 5260 autosampler, a 5110 pump and a 5430 diode array detector. Separation was achieved using an octadecyl silica gel semipreparative column (Onyx, 10 mm × 100 mm, Phenomenex, Torrance, CA, USA) equipped with a guard column. Further purification was achieved on a Synergi Polar-RP 80 Å column (250 × 4.6 mm, Phenomenex, Torrance, CA, USA). Circular dichroism spectroscopy was performed in MeOH on a J-810 CD spectrometer (Jasco, Pfungstadt, Germany) with a 0.5-mm cuvette path length. The averages of triplicate scans were acquired and the CD signal of the MeOH was subtracted subsequently.

### 4.2. Fungal Collection and Taxonomy

The fungus *Cosmospora* sp. (GenBank accession number MH79129) was previously isolated from the Baltic Sea environment [4]. The plant pathogen *M. oryzae* was obtained from Deutsche Sammlung für Mikroorganismen und Zellkulturen (DSMZ, Braunschweig, Germany). *Cosmospora* sp. And *M. oryzae* were individually maintained on PDA medium (potato infusion powder 4 g, glucose monohydrate 20 g, agar 15 g in 1 L) as pre-cultures for 14 days.

### 4.3. UPLC-QtoF-MS Analysis

Analysis of all crude extracts (0.1 mg/mL) and controls (media blanks and solvents) were performed on an UPLC I-Class system coupled to a Xevo G2-XS QtoF Mass spectrometer (Waters^®^, Milford, MA, USA). Chromatography was achieved on an Acquity UPLC HSS T3 column (High Strength Silica C18, 1.8 μm, 100 × 2.1 mm, Waters^®^, Milford, MA, USA) maintained at 40 °C. A binary solvent system (A: 99.9% MilliQ^®^-water/0.1% formic acid ULC/MS grade and B: 99.9% acetonitrile ULC/MS grade/0.1% formic acid) was pumped at a flow rate of 0.4 mL/min with a gradient of 1% to 100% B in 11.5 min. The column was then washed for 3.5 min with 100% B, back to 1% B (initial condition) and reconditioned at 1% in 2.5 min. MS and MS/MS spectra were acquired in a data dependent analysis (DDA) mode with an electrospray ionization (ESI) source in the positive ionization mode. The *m/z* range was set to 50–1200 Da in the centroid data format. The QToF-MS source temperature was set to 150 °C, capillary voltage of 0.8 KV, cone and desolvation gas flow of 50 and 1200 L/h, respectively. Desolvation temperature was set to 550 °C with sampling cone and source offset at 40 and 80, respectively. Collision energy (CE) was ramped: low CE from 6-60 eV to high CE of 9-80 eV. As controls, solvent (MeOH) and non-inoculated medium were injected under the same conditions.

### 4.4. Molecular Networking and Annotations

A molecular family, as introduced by Nguyen et al. [41], describes structurally related compounds based on the similarity in mass spectral fragmentation patterns. A series of connected nodes in an MN typically represents a molecular family. UPLC-MS/MS RAW data files were converted to centroid .mzXML format using msconvert from the ProteoWizard suite version 3.0.10051 [42]. The converted .mzXML files were uploaded on Global Natural Products Social Molecular Networking (GNPS) platform with an open access File Transfer Protocol client WinSCP version 5.15.9. A molecular network was generated from the .mzXML files of the fungal cultures using the GNPS script suite [43]. The algorithm assumed precursor mass tolerance of 0.02 Da and a product ion tolerance of 0.02 Da to create consensus spectra. The network was generated using a minimum of 6 matched peaks and a defined cosine score above 0.7. The resulting MN was visualized using Cytoscape version 3.8.2 [44], and displayed with ‘directed’ style, with the edges modulated by cosine score. To simplify analysis of the network, only nodes that contained ions observed between retention times of 2-11.30 min were considered. Background nodes originating from the cultivation medium PDA and solvents (MeOH and EtoAc) were removed from the MN. Node colours were mapped based on the source of the spectra files, thus red for *Cosmospora* sp., green for *M. oryzae* and blue for the co-culture. The MN job on GNPS can be found at https://gnps.ucsd.edu/ProteoSAFe/status.jsp?task=2d3d301a8f3e43039432865c61f33028, accessed on 01 December 2021. Manual annotations were done using MassLynx^®^ (version 4.2, Waters^®^, Milford, MA, USA). to predict the molecular formulae of the *m/z* [M+H]^+^ or other adduct ions, e.g., *m/z* [M+Na]^+^, and searching them against databases such us NPAtlas [45] and DNP [46]. Hits were considered based on retention times and fragmentation patterns of the ions and biological sources of the hits. Dereplication was also achieved by isolation and characterization by NMR and other data such as HR-ESIMS, IR and [α]_D_. Identifications were also assigned confidence levels 1–4 as proposed by Sumner et al. [47].

### 4.5. Large Scale Co-Cultivation and Isolation of Compounds

About 1 cm plugs from each pre-culture, *Cosmospora* sp. and *M. oryzae*, were streaked and inoculated on opposite sides of the same agar plate. To obtain enough extract amount for compound isolation, 100 plates on PDA medium were inoculated. The co-culture plates were incubated at 22 °C in the dark for 21 days. Co-cultures were extracted twice with EtOAc (at a ratio of 4 plates: 200 mL of EtOAc) after homogenising with an Ultra-Turrax at 19,000 rpm. EtOAc extracts were washed with equal volumes of water (200 mL). The pooled organic phase was concentrated in vacuo to yield 1.01 g of dry organic extract, and then partitioned using a modified protocol originally designed by Kupchan and Tsou [15,29]. This resulted in three subextracts: *n*-hexane (KH, 314.1 mg), CH_2_Cl_2_ (KC, 646.7 mg), and aqueous MeOH (KM, 7.1 mg). The subextracts were analysed by UPLC-MS/MS, and ions of interest (compounds **1**-**5**) were annotated in KC. The KC extract was then fractionated over a Chromabond SPE C18 column cartridge (Macherey-Nagel, Düren, Germany) with a step gradient of 10% to 100% MeOH to afford 11 fractions (F1-F11). Each subfraction was checked for its composition by TLC using CHCl_3_-CH_3_OH (9.5:0.5 *v*/*v*) as mobile phase and vanillin sulfuric acid as visualization reagent. Fractions 7 and 8 (F7–8) were combined (250 mg) and purified by HPLC on a semi-preparative RP column (Phenomex, Onyx monolythic C18, 100 × 10 mm) with H_2_O:MeCN mixture (70:30 to 0:100 in 17 min, flow rate 3.5 mL/min) as mobile phase to yield compounds **6** (4 mg, *t*_R_ 12.3 min) and **1** (2.8 mg, *t*_R_ 14.5 min). Combined fractions 5 and 6 (33 mg) were subjected to semi-preparative RP–HPLC, on the same column, eluting with H_2_O:MeCN (64:36 to 0:100 in 11 min, flow rate 3.5 mL/min) to yield 6 subfractions (fraction F5–6.1 to 5–6.6). Subfraction 5–6.1 contained pure compound **7** (5 mg, *t*_R_ 3.6 min), while 5–6.3 contained pure compound **3** (0.6 mg, *t*_R_ 6.5 min). Subfraction 5–6.2 (2.4 mg, *t*_R_ 4–6 min) was re-chromatographed by RP-HPLC on a Xselect HSS T3 column (2.5 µm, 150 × 4.6 mm) using an isocratic mixture of H_2_O:MeCN (50:50) and flow rate of 1 mL/min, to afford compounds **2** (1.5 mg, *t*_R_ 4.1 min), **4** (0.5 mg, *t*_R_ 5.2 min) and **5** (0.2 mg, *t*_R_ 5.9 min). Subfraction 5–6.5 was also purified by RP-HPLC on a Synergi 4u Polar-RP 80A (250 × 4.6 mm) using an isocratic mixture of H_2_O:MeCN (35:65) and flow rate of 1 mL/min to yield **8** (2.2 mg, *t*_R_ 4.1 min) and **9** (1.2 mg, *t*_R_ 4.3 min). In an effort to obtain sufficient amounts of **1**, KH was fractionated over modified silica SPE cartridge (CHROMOBOND^®^, Macherey-Nagel, Dueren, Germany) with a step gradient of EtOAc in *n*-hexane (0 to 100% EtoAc) to afford 11 fractions (H1-H11). Compound **10** (10 mg) was crystallised out of fraction H2 and purified by filtering.

Soudanone A (**1)**: amorphous powder;$\;{\left[\alpha \right]}_{D}^{20}$-59 (*c* 0.5, MeOH); IR (film) *ν*_max_ 3251, 2928, 2856, 1645, 1614, 1463, 1386, 1246, 1142 cm^−1^; ^1^H (600 MHz) and ^13^C (150 MHz) NMR data, see Table 1 and Table 2; HR-ESIMS *m/z* 293.1755 [M+H]^+^ (calcd for C_17_H_25_O_4_, 293.1753).

Soudanone E (**2**): yellow amorphous powder;$\;{\left[\alpha \right]}_{D}^{20}$-50 (*c* 0.05, MeOH); IR (film) *ν*_max_ 3316, 2930, 2858, 1644, 1618, 1460, 1382, 1253, 1109 cm^−1^; ^1^H (600 MHz) and ^13^C (150 MHz) NMR data, see Table 1 and Table 2; HR-ESIMS *m/z* 309.1705 [M+H]^+^ (calcd for C_17_H_25_O_5_, 309.1702).

Soudanone D (**3**): yellow amorphous powder;$\;{\left[\alpha \right]}_{D}^{20}$-56 (*c* 0.35, MeOH); IR (film) *ν*_max_ 3252, 2930, 2861, 1705, 1655, 1618, 1465, 1379, 1254, 1174 cm^−1^; ^1^H (600 MHz) and ^13^C (150 MHz) NMR data, see Table 1 and Table 2; HR-ESIMS *m/z* 307.1546 [M+H]^+^ (calcd for C_17_H_23_O_5_, 307.1545).

Soudanone H (**4**): yellow amorphous powder;$\;{\left[\alpha \right]}_{D}^{20}$-44 (*c* 0.05, MeOH); IR (film) *ν*_max_ 3390, 2918, 2850, 1646, 1598, 1464, 1380, 1255, 1179 cm^−1^; ^1^H (600 MHz) and ^13^C (150 MHz) NMR data, see Table 1 and Table 2; HR-ESIMS *m/z* 309.1703 [M+H]^+^ (calcd for C_17_H_25_O_5_, 309.1702).

Soudanone I (**5**): yellow amorphous powder;$\;{\left[\alpha \right]}_{D}^{20}$-20 (*c* 0.07, MeOH); IR (film) *ν*_max_ 3311, 2948, 2864, 1729, 1648, 1616, 1382, 1253, 1174 cm^−1^; ^1^H (600 MHz) and ^13^C (150 MHz) NMR data, see Table 1 and Table 2; HR-ESIMS *m/z* 309.1341 [M+H]^+^ (calcd for C_16_H_21_O_6_, 309.1338).

### 4.6. Co-Cultivation by Overlaid Inoculation on PDA and Extraction

A 1 cm agar plug of *M. oryzae* pre-culture was streaked over the surface of an agar plate and incubated at 22 °C for 7 days until it formed a lawn on the agar surface. Agar plugs of *Cosmospora* sp. were then inoculated onto the *M. oryzae* lawn (overlaid inoculation) and incubated for 14 days, resulting in a total incubation period of 21 days. For extraction, the entire co-culture was chopped into pieces and extracted with EtOAc (2 × 20 mL) and extracted as previously described [48]. Briefly, the co-culture was homogenized by an Ultra-Turrax in 20 mL EtOAc, and the organic layer decanted into a separating funnel. The extraction with EtOAc was done twice to increase the efficiency. The pooled EtOAc layer was then washed twice with 20 mL of Milli-Q^®^ water (Arium^®^ Lab water systems, Sartorius). The EtOAc phase was collected and dried under vacuum, re-dissolved in methanol (3 mL of ULC/MS grade MeOH), filtered through a 0.2 μm PTFE membrane (VWR International, Darmstadt, Germany) into a pre-weighed vial, and dried under nitrogen. An aliquot (0.1 mg/mL in MeOH) of extract was prepared for analysis by UPLC-MS/MS, and remaining extract was stored at −20 °C.

### 4.7. Co-Cultivation in Liquid Broth (PDB)

About 3 cm agar plugs, each of *Cosmospora* sp. and *M. oryzae* precultures, were inoculated into a 2 L Erlenmeyer flask containing 500 mL of potato dextrose broth (PDB: potato infusion powder 4 g, glucose monohydrate 20 g in 1 L; pH 5.6) and incubated at 22 °C on an orbital shaker (VKS-75 control, Edmund Bühler, Hechingen, Germany) at 120 rpm. An aliquot of the broth (5 mL) was sampled each day with a sterile pipette for 21 days. The daily aliquots were extracted with EtOAc (10 mL) and washed twice with 10 mL of Milli-Q^®^ water (Arium^®^ Lab water systems, Sartorius). Aliquots (0.1 mg/mL in MeOH) of extracts were prepared for analysis by UPLC-MS/MS and remaining extracts were stored at −20 °C.

### 4.8. Biological Assays

Anti-phytopathogenic activity assays were performed using the broth dilution technique in 96-well microplates. Compounds **1**-**10** (except **4** and **5**) were tested for in vitro anti-phytopathogenic activity against six phytopathogens, i.e., four bacteria (*X. campestris, R. solanacearum, P. syringae, and E. amylovora*), a fungus (*M. oryzae*), and an oomycete (*P. infestans*). The selected plant pathogens are among the most widespread and devastating microbes and pose a direct impact on global food supply and forest products [49,50]. The protocol used was described in a previous publication [4]. Briefly, compounds were first dissolved in DMSO (effective conc. 0.5% (*v*/*v*)) and added to test organisms suspended in appropriate broth in 96-well microplates. Compounds were tested at varying concentrations (max test conc. 100 μg/mL) in triplicate. Chloramphenicol and tetracycline were used as positive controls in the antibacterial tests, nystatin in the antifungal test, and cycloheximide in the antioomycete test; growth media and 0.5% (*v*/*v*) DMSO were tested as negative controls. Optical density measurements were made at 600 nm using an Infinite M200 reader (TECAN Deutschland GmbH, Crailsheim, Germany) before and after incubation to obtain growth inhibition values. The IC_50_ values were estimated using the Microsoft Excel program.

### 4.9. Mosher’s Esterification

Compounds **2** and **4** (approximately 0.2 mg and 0.1 mg, respectively) were treated with (trimethylsilyl)diazomethane to protect the phenolic OH groups prior to MPA derivatization. The reaction product was split into two aliquots, which were treated separately with a ten-fold excess of (*R*)- and (*S*)-MPA (methoxyphenylacetic acid), respectively, in the presence of EDC (1-ethyl-3-(3-dimethylaminopropyl)carbodiimide) and 4-DMAP (4-dimethylaminopyridine), to give the corresponding (*R*)- and (*S*)-MPA esters. The structure of the esterification products was confirmed by LC-MS, ^1^H NMR and 1D-TOCSY experiments (detailed description in Supplementary Materials, Figures S50–S54).

## 5. Conclusions

Five isochromanones, including two new soudanones H-I (**4–5**), with varying anti-phytopathogenic activities were isolated and identified in the co-culture of a marine-derived fungus, *Cosmospora* sp., and the phytopathogen, *M. oryzae*, but were not produced in the axenic fungal cultures. This supports the hypothesis that using phytopathogens as model competitors in co-cultivation triggers the biosynthesis of a new bioactive and diverse chemistry in marine fungi. This is the first study to report on isochromans and isochromanones from *Cosmospora* and its co-culture, and the second on the chemical class of soudanones from fungi. MN was used as an essential metabolomics tool to unravel the putative producer of the induced compounds in the co-culture. It further facilitated the annotation and structural elucidation of the isolated compounds. Further investigation into the optimal fermentation conditions to enhance the production of the isochromanones (**1–5**), as well as to elucidate the BGC responsible for their biosynthesis will be the focus of our future work. This will expand our knowledge on the influence of the conditions of co-cultivation on the expression of BGCs and biosynthesis of metabolites in these two fungi.

## Figures and Tables

**Table 1:** ^1^H NMR data of compounds **1–5** (^a^ acquired in CD_3_OD at 500 MHz, ^b^ acquired in CDCl_3_ at 600 MHz), δ in ppm, Mult (*J* in Hz).

Position	1 ^a^	1 ^b^	2 ^b^	3 ^b^	4 ^b^	5 ^b^
**3**	4.47 m	4.46 m	4.45 m	4.45 m	4.47 m	4.46 m
**4**	3.03 dd (16.7, 3.4) 2.69 dd (16.7,11.5)	2.94 dd (16.5, 3.4) 2.70 dd (16.5, 11.6)	2.93 dd (16.5, 3.2) 2.69 dd (16.5, 11.8)	2.93 dd (16.5, 3.3) 2.69 dd (16.5, 11.7)	2.94 dd (16.5, 3.2) 2.71 dd (16.5, 11.8)	2.94 dd (16.5, 3.3) 2.70 dd (16.5, 11.7)
**7**	6.24 s	6.31 s	6.31 s	6.30 s	6.29 s	6.3 s
**1’**	1.83 m 1.76 m	1.88 m 1.73 m	1.87 m 1.73 m	1.86 m 1.76 m	1.90 m 1.75 m	1.90 m 1.76 m
**2’**	1.58 m 1.48 m	1.56 m 1.45 m	1.60 m 1.48 m	1.62 m 1.48 m	1.64 m 1.52 m	1.64 m 1.53 m
**3’**	1.35 m	1.34 m	1.37 m	1.36 m	1.52 m 1.42 m	1.70 m
**4’**	1.35 m	1.32 m	1.36 m 1.44 m	1.62 m	1.53 m 1.45 m	2.36 t (7.3)
**5’**	1.35 m	1.26 m	1.45 m	2.45 t (7.3)	3.54 m	
**6’**	1.35 m	1.29 m	3.81 m		1.52 m 1.44 m	3.68 s
**7’**	0.91 t (6.9)	0.88 t (6.9)	1.20 d (6.1)	2.15 s	0.95 t (7.5)	
**5-Me**	2.04 s	2.07 s	2.06 s	2.06 s	2.07 s	2.07 s
**OH**		11.26 s	11.26 s	11.18 s	11.26 s	11.26 s

**Table 2:** ^13^C NMR data of compounds **1**-**5** (150 MHz, CDCl_3_).

Position	1	2	3	4	5
1	170.6, C	170.6, C	170.5, C	170.5, C	170.4, C
3	78.7, CH	78.6, CH	78.5, CH	78.5, CH	78.3, CH
4	30.6, CH_2_	30.7, CH_2_	30.7, CH_2_	30.7, CH_2_	30.6, CH_2_
4a	139.5, C	139.4, C	139.4, C	139.5, C	139.4, C
5	113.1, C	113.2, C	113.0, C	112.9, C	113.0, C
6	160.9, C	161.0, C	160.7, C	160.5, C	160.6, C
7	101.6, CH	101.6, CH	101.6, CH	101.6, CH	101.7, CH
8	162.5, C	162.5, C	162.5, C	162.5, C	162.6, C
8a	102.1, C	102.0, C	102.1, C	102.2, C	102.2, C
1’	35.1, CH_2_	35.0, CH_2_	34.9, CH_2_	35.1, CH_2_	34.8, CH_2_
2’	25.1, CH_2_	25.0, CH_2_	24.9, CH_2_	25.6, CH_2_	24.6, CH_2_
3’	29.5, CH_2_	29.4, CH_2_	29.0, CH_2_	25.2, CH_2_	24.8, CH_2_
4’	29.3, CH_2_	25.7, CH_2_	23.6, CH_2_	36.8, CH_2_	34.0, CH_3_
5’	31.9, CH_2_	39.2, CH_2_	43.7, CH_2_	73.3, CH	174.1, C
6’	22.8, CH_2_	68.4, CH	209.3, C	30.4, CH_2_	51.7, CH_3_
7’	14.2, CH_3_	23.7, CH_3_	30.1, CH_3_	10.0, CH_3_	
5-Me	10.6, CH_3_	10.6, CH_3_	10.6, CH_3_	10.6, CH_3_	10.6, CH_3_

**Table 3:** Anti-phytopathogenic activity (IC_50_ values in µg/mL) of isolated compounds against phytopathogens. Compounds **4** and **5** were not tested due to their minute amounts. Positive controls for *Pseudomonas syringae (*Ps*), Xanthomonas campestris* (Xc) and *Erwinia amylovora* (Ea): chloramphenicol; *Ralstonia solanacearum* (Rs): tetracycline; *Phytophthora infestans* (Pi): cycloheximide, *Magnaporthe oryzae* (Mo): nystatin.

Compound	Bacteria				Oomycete	Fungus
	Ps	Xc	Ea	Rs	Pi	Mo
**1**	>100	>100	>100	>100	>100	>100
**2**	>100	71.5	>100	>100	27.6	12.8
**3**	>100	15.7	>100	>100	52.1	60.3
**6**	23.4	7.4	>100	>100	3.2	0.8
**7**	>100	67.1	>100	42.2	>100	>100
**8**	95.7	12.1	>100	27.6	2.3	>100
**9**	>100	21.7	>100	>100	7.2	>100
**10**	>100	>100	>100	>100	>100	>100
Control	0.4	0.5	0.2	1.0	0.6	0.4

## Data Availability

Not applicable.

## Supplementary Materials

The following supporting information can be downloaded at: https://www.mdpi.com/article/10.3390/ijms23020782/s1.
