# Supplementary material for: Induction of Isochromanones by Co-Cultivation of the Marine Fungus Cosmospora sp. and the Phytopathogen Magnaporthe oryzae
Source: Int J Mol Sci. 2022 Jan 11;23(2):782. doi: 10.3390/ijms23020782 (PMC8775470; doi:10.3390/ijms23020782)
Supplement: Supplementary file 1 [file ijms-23-00782-s001.zip › ijms-1537620-supplementary.pdf]

Supporting Information

Induction of isochromanones by co-cultivation of the marine fungus *Cosmospora* sp. and the phytopathogen *Magnaporthe oryzae*

Ernest Oppong-Danquah [1], Martina Blümel [1], Silvia Scarpato [2], Alfonso Mangoni [2] and Deniz Tasdemir [1,3*]

[1] GEOMAR Centre for Marine Biotechnology (GEOMAR-Biotech), Research Unit Marine Natural Products Chemistry, GEOMAR Helmholtz Centre for Ocean Research Kiel, Am Kiel-Kanal 44, 24106 Kiel, Germany

[2] Dipartimento di Farmacia, Università degli Studi di Napoli Federico II, via Domenico Montesano 49, 80131 Napoli, Italy

[3] Faculty of Mathematics and Natural Science, Kiel University, Christian-Albrechts-Platz 4, 24118 Kiel, Germany

* Correspondence: dtasdemir@geomar.de; Tel.: +49-431-6004430

List of Tables Page

Table S1. Putative annotation of metabolites produced in the axenic cultures of *Cosmospora* sp. and *M. oryzae*, and their co-culture 4

Table S2. In vitro anti-phytopathogenic activity (IC_{50} values µg/mL) of the Kupchan subextracts *n*-hexane, CH_2Cl_2 and aqueous MeOH (Aq.) 8

Table S3. Comparison of the ^1H NMR and ^{13}C NMR data of pseudoanguillosporin B (**7**) and soudanone E (**2**) with focus on the aliphatic side chain, ([a] acquired in CD_3OD, [b] acquired in $CDCl_3$, [C] data from literature (acquired in CD_3OD), δ in ppm, *J* in Hz 9

List of Figures

Figure S1. ^1H NMR spectrum of compound **1** ($CDCl_3$, 600 MHz) 10

Figure S2. ^{13}C NMR spectrum of compound **1** ($CDCl_3$, 150 MHz) 10

Figure S3. COSY spectrum of compound **1** ($CDCl_3$, 600 MHz) 11

Figure S4. HSQC spectrum of compound **1** ($CDCl_3$, 600/150 MHz) 11

Figure S5. HMBC spectrum of compound **1** ($CDCl_3$, 600/150 MHz) 12

Figure S6. TOCSY spectrum of compound **1** ($CDCl_3$, 600 MHz) 12

Figure S7. NOESY spectrum of compound **1** ($CDCl_3$, 600 MHz) 13

Figure S8. ^1H NMR spectrum of compound **1** (CD_3OD, 500 MHz) 13

Figure S9. HR-ESIMS spectrum of compound **1** 14

Figure S10. HR-ESIMS/MS spectrum of compound **1** 14

Figure S11. FT-IR spectrum of compound **1** 14

Figure S12. ^{1}H NMR spectrum of compound **2** ($CDCl_3$, 600 MHz) 15

Figure S13. ^{13}C spectrum of compound **2** ($CDCl_3$, 150 MHz) 15

Figure S14. COSY spectrum of compound **2** ($CDCl_3$, 600 M 16

Figure S15. HSQC spectrum of compound **2** ($CDCl_3$, 600/150 MHz 16

Figure S16. HMBC spectrum of compound **2** ($CDCl_3$, 600/150 MHz) 17

Figure S17. NOESY spectrum of compound **2** ($CDCl_3$, 600 MHz) 17

Figure S18. HR-ESIMS spectrum of compound **2** 18

Figure S19. HR-ESIMS/MS spectrum of compound **2** 18

Figure S20. FT-IR spectrum of compound **2** 18

Figure S21. ^{1}H NMR spectrum of compound **3** ($CDCl_3$, 600 MHz) 19

Figure S22. ^{13}C NMR spectrum of compound **3** ($CDCl_3$, 150 MHz) 19

Figure S23. COSY spectrum of compound **3** ($CDCl_3$, 600 MHz) 20

Figure S24. HSQC spectrum of compound **3** ($CDCl_3$, 600/150 MHz) 20

Figure S25. HMBC spectrum of compound **3** ($CDCl_3$, 600/150 MHz) 21

Figure S26. NOESY spectrum of compound **3** ($CDCl_3$, 600 MHz) 21

Figure S27. HR-ESIMS spectrum of compound **3** 22

Figure S28. HR-ESIMS/MS spectrum of compound **3** 22

Figure S29. FT-IR spectrum of compound **3** 22

Figure S30. ^{1}H NMR spectrum of compound **4** ($CDCl_3$, 600 MHz) 23

Figure S31. ^{13}C NMR spectrum of compound **4** ($CDCl_3$, 150 MHz) 23

Figure S32. COSY spectrum of compound **4** ($CDCl_3$, 600 MHz) 24

Figure S33. HSQC spectrum of compound **4** ($CDCl_3$, 600/150 MHz) 24

Figure S34. HMBC spectrum of compound **4** ($CDCl_3$, 600/150 MHz) 25

Figure S35. NOESY spectrum of compound **4** ($CDCl_3$, 600 MHz) 25

Figure S36. HR-ESIMS spectrum of compound **4** 26

Figure S37. HR-ESIMS/MS spectrum of compound **4** 26

Figure S38. FT-IR spectrum of compound **4** 26

Figure S39. ^{1}H NMR spectrum of compound **5** ($CDCl_3$, 600 MHz) 27

Figure S40. ^{13}C NMR spectrum of compound **5** ($CDCl_3$, 150 MHz) 27

Figure S41. COSY spectrum of compound **5** ($CDCl_3$, 600 MHz) 28

Figure S42. HSQC spectrum of compound **5** ($CDCl_3$, 600/150 MHz) 28

Figure S43. HMBC spectrum of compound **5** ($CDCl_3$, 600/150 MHz) 29

Figure S44. NOESY spectrum of compound **5** ($CDCl_3$, 600 MHz) 29

Figure S45. HR-ESIMS spectrum of compound **5** 30

Figure S46. HR-ESIMS/MS spectrum of compound **5** 30

Figure S47. FT-IR spectrum of compound **5** 30

Figure S48. Agar plate (9 cm Petri dish) of overlaid co-cultivation of *Cosmospora* sp. on *M. oryzae* after 21 days. UPLC chromatogram of the extract shows the expression of compounds **1-5** with **2-4** in very low intensity. 31

Figure S49. UPLC chromatograms of the extracts of co-cultures of *Cosmospora* sp. and *M. oryzae* in potato dextrose broth (PDB) at selected time points (Day 2, 3, 6, 9, 13, 14, 16 and 21). Extracted ion chromatogram (XIC) of compounds **8/9**, showing their biosynthesis from day 3 of co-cultivation in PDB medium. 32

Figure S50. Mosher's ester analysis scheme for compound **2** 33

Figure S51. LC-MS chromatograms of the (*R*)- and (*S*)-MPA reaction mixtures from compound **2** 34

Figure S52. NMR analysis of the (*R*)- and (*S*)-MPA reaction mixtures from compound **2** 34

Figure S53. Mosher's ester analysis scheme for compound **4** 35

Figure S54. LC-MS chromatograms of the (*R*)-MPA reaction mixture from compound **4** 35

References 36

Table S1. Putative annotation of metabolites produced in the axenic cultures of *Cosmospora* sp. and *M. oryzae*, and their co-culture. Annotation/identification was based on the *m/z* $[M+H]^+$ or other adducts (specified), retention time (t_R), predicted molecular formula, fragmentation pattern and spectral data analysis. The source of the compound is indicated as B - co-culture, R - *Cosmospora* sp. and G - *M. oryzae*. Confidence level of annotation are given based on the reporting standards (1- 4) proposed by Sumner, *et al.* [1] where 1 is identified compound - 4 is unknown compound.

Ser. No.	Putative ID/ chemical family	Structure	Molecular formula (*m/z*)	Parent mass *m/z* $[M+H]^+$	MS/MS fragmentation	t_R / source	Confidence / Reference
1	Soudanone A (isochromanone)	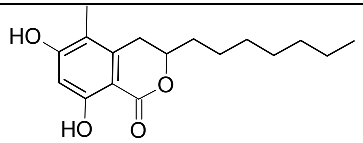	$C_{17}H_{25}O_4$ $[M+H]^+$	293.1755	275.1652; 265.1807; 191.0713; 137.0602	8.77 B	1 [2]
2	Soudanone E (isochromanone)	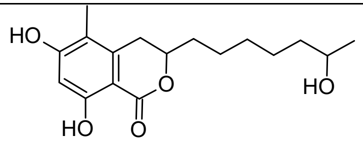	$C_{17}H_{25}O_5$ $[M+H]^+$	309.1705	273.1491; 255.1376; 191.0704; 137.0592	5.91 B	1 [2]
3	Soudanone D (isochromanone)	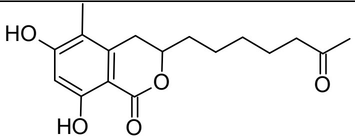	$C_{17}H_{23}O_5$ $[M+H]^+$	307.1546	289.1442; 271.1331; 229.1230; 179.0704	6.17 B	1 [2]
4	Soudanone H (isochromanone)	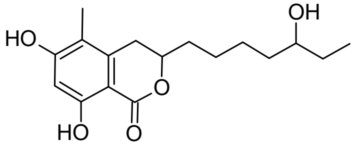	$C_{17}H_{25}O_5$ $[M+H]^+$	309.1703	273.1492; 255.1387; 203.0710; 181.0503; 163.0760; 137.0604	5.95 B	1
5	Soudanone I (isochromanone)	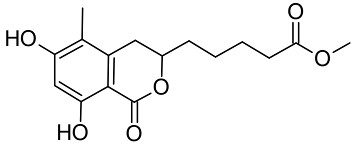	$C_{16}H_{21}O_6$ $[M+H]^+$	309.1341	277.1082; 231.1028; 215.1077; 203.1076; 137.0605	6.07 B	1
6	Pseudoanguillosporin A (isochroman)	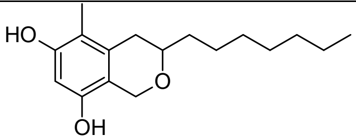	$C_{17}H_{27}O_3$ $[M+H]^+$	279.1939	260.1745; 151.0768	4.77 R/B	1 [3]

7	Pseudoanguillosporin B (isochroman)	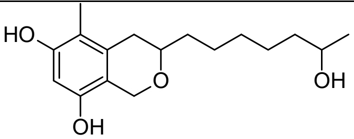	$C_{17}H_{27}O_4$ $[M+H]^+$	295.1912	277.1805; 259.1705; 151.0754	8.17 R/B	1 [3]
8	Cephalochromin (naphtho-γ-pyrone)	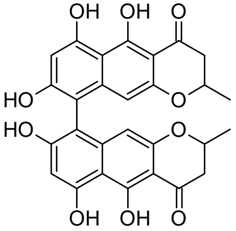	$C_{28}H_{23}O_{10}$ $[M+H]^+$	519.1284	501.1195, 260.0698, 245.0457, 231.0683, 219.0297, 218.0229	7.46 R/B	1 [3]
9	Ustilaginoidin G/ Dihydroisoustilaginoidin A (naphtho-γ-pyrone)	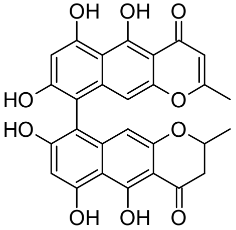	$C_{28}H_{21}O_{10}$ $[M+H]^+$	517.1150	499.1036, 260.0694, 258.0541, 245.0427, 231.0701, 219.0291	7.31 R/B	1 [4]
10	Ergosterol (steroid)	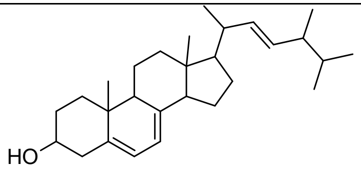	$C_{28}H_{44}ONa$ $[M+Na]^+$	419.3290	300.8700; 253.2001; 159.12	10.96 R/B	1 [5]
11	Ustilaginoidin A (naphtho-γ-pyrone)	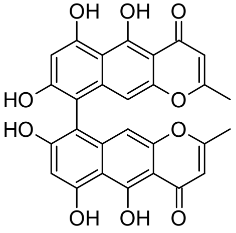	$C_{28}H_{19}O_{10}$ $[M+H]^+$	515.1259	258.0501	7.23 R/B	2 [4]

12	Ustilaginoidin H or I (naphtho-γ-pyrone)	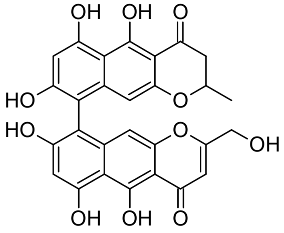	$C_{28}H_{21}O_{11}$ $[M+H]^+$	533.1090	515.0963, 493.1126, 477.0819, 451.0999, 260.0693	7.8 R/B	2 [4]
13	Ustilaginoidin V (naphtho-γ-pyrone)	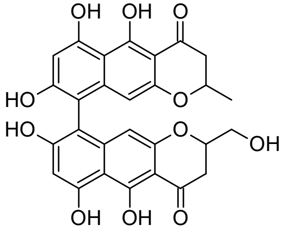	$C_{28}H_{23}O_{11}$ $[M+H]^+$	535.1237	517.1116, 493.1128, 477.0807, 451.1015, 260.0688, 517.1116, 493.1128, 477.0807, 451.1015, 260.0688	7.85 R/B	2 [6]
14	Ustilaginoidin E (naphtho-γ-pyrone)	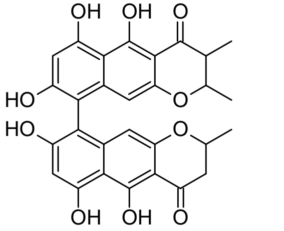	$C_{29}H_{25}O_{10}$ $[M+H]^+$	533.1479	515.1333, 490.1250, 477.0801, 274.0849, 260.0694	8.1 R/B	2 [4]
15	Ustilaginoidin D (naphtho-γ-pyrone)	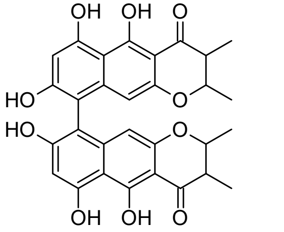	$C_{30}H_{27}O_{10}$ $[M+H]^+$	547.1599	529.1428, 504.1302, 491.1051, 274.0846, 201.0479	8.25 R/B	2 [4]

16	Ustilaginoidin M (naphtho-γ-pyrone)	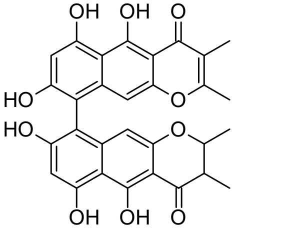	$C_{30}H_{25}O_{10}$ $[M+H]^+$	545.1447	464.2164, 424.1467, 272.0674	8.62 R/B	2 [6]
17	Ustilaginoidin B (naphtho-γ-pyrone)	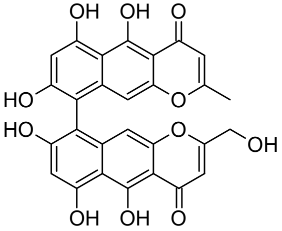	$C_{28}H_{20}O_{11}$ $[M+H]^+$	531.1321	272.0701; 260.0700	8.1 R/B	2 [4]
18	Acuminatum C (cyclic peptide)	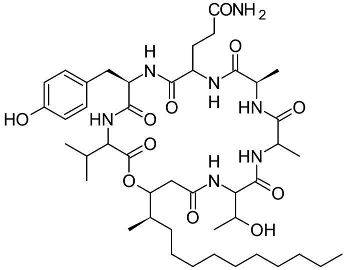	$C_{44}H_{72}N_7O_{11}$ $[M+H]^+$	874.5460	856.5190, 551.2844, 466.3281, 434.2038, 395.2917, 377.2818, 271.1421, 200.1040, 101.0707	8.14 R/B	3 [7]
19	Acuminatum B (cyclic peptide)	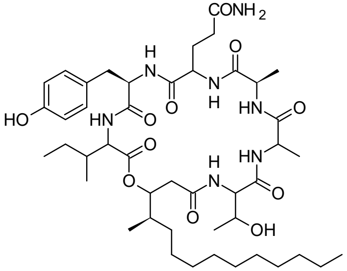	$C_{45}H_{73}N_7O_{11}$ $[M+H]^+$	888.5449	870.5326, 565.2996, 466.3303, 434.2056, 395.2923, 271.1425	8.44 R/B	3 [7]

20	Dehydroergosterol<br>(steroid)	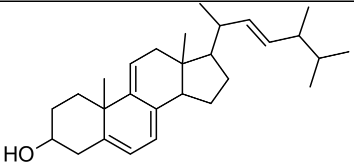	$C_{28}H_{42}O$<br>$[M-H_2O+H]^+$	377.3200	309.2600; 253.2001; 159.1202	10.9<br>R/B	3<br>[8]
21	Phosphatidylcholine<br>(18:2/0:0)<br>(phospholipid)	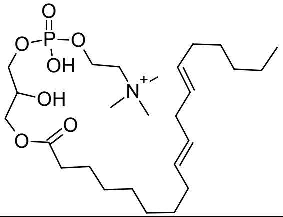	$C_{26}H_{51}N_7OP$<br>$[M+H]^+$	520.3740	184.0751	7.7<br>G/B	3<br>[9]

Table S2. In vitro anti-phytopathogenic activity (IC_{50} values μg/mL) of the Kupchan subextracts *n*-hexane, CH_2Cl_2 and aqueous MeOH. Test phytopathogens include Ps, *P. syringae*; Xc, *X. campestris*, Ea, *E. amylovora*; Rs, *R. solanacearum*; Pi, *P. infestans*; Mo. *M. oryzae*. DMSO (0.5%) was used as a solvent control. Positive controls for Xc, Ea and Ps: chloramphenicol, for Rs: tetracycline, for Mo: nystatin and for Pi: cycloheximide.

Kupchan subextracts	Ps	Xc	Ea	Rs	Pi	Mo
Aq. MeOH	> 100	> 100	> 100	> 100	> 100	> 100
CH_2Cl_2	> 100	10.5	> 100	99	8,5	3.9
n-hexane	> 100	> 100	> 100	>100	> 100	> 100
Positive control	0.7	0.5	0.7	1.0	0.3	0.4

Table S3. Comparison of the ^{1}H NMR and ^{13}C NMR data of pseudoanguillosporin B (**7**) and soudanone E (**2**) with focus on the aliphatic side chains, ([a] acquired in CD_3OD, [b] acquired in $CDCl_3$, [c] data obtained from literature [3] (acquired in CD_3OD), δ in ppm, *J* in Hz.

**Position**	^{1}H NMR data			^{13}C NMR data		
	**7**[c]	**7**[a]	**2**[a]	**7**[c]	**7**[b]	**2**[b]
**1**	4.50 d (14.7)	4.49 d (14.7)		64.6, CH_2	64.6, CH_2	170.6, C
	4.84 d (14.7)	4.81 d (14.7)				
**2**						
**3**	3.51 m	3.55 m	4.48 m	74.9, CH	74.7, CH	78.6, CH
**4**	2.33 dd	2.35 dd	3.04 dd	32.3, CH_2	32.6, CH_2	30.7, CH_2
	(16.6, 10.8)	(16.6, 10.7)	(16.6, 3.4)			
	2.59 dd (16.6, 2.2)	2.61 dd (16.6, 2.3)	2.69 dd (16.6, 11.5)			
**4a**				133.4, C	134.9, C	139.4, C
**5**				112.7, C	113.7, C	113.2, C
**6**				153.5, C	152.4, C	161.0, C
**7**	6.21 s	6.19 s	6.24 s	99.6, CH	100.4, CH	101.6, CH
**8**				150.8, C	149.8, C	162.5, C
**8a**				112.8, C	113.6, C	102.0, C
**1'**	1.49 m	1.47 m	1.83 m	35.8, CH_2	36.2, CH_2	35.0, CH_2
			1.76 m			
**2'**	1.49 m	1.47 m	1.61 m	25.5, CH_2	25.8, CH_2	25.0, CH_2
			1.46 m			
**3'**	1.49 m	1.47 m	1.46	29.5, CH_2	29.8, CH_2	29.4, CH_2
**4'**	1.49 m	1.47 m	1.46 m	25.3, CH_2	25.7, CH_2	25.7, CH_2
			1.39 m			
**5'**	1.49 m	1.47 m	1.47 m	38.8, CH_2	39.4, CH_2	39.2, CH_2
			1.38 m			
**6'**	3.73 m	3.73 m	3.72 m	67.3, CH	68.3, CH	68.4, CH
**7'**	1.17 d (6.2)	1.16 d (6.3)	1.15 d (6.2)	22.2, CH_3	23.5, CH_3	23.7, CH_3
**5-Me**	1.98 s	1.98 s	2.04 s	9.0, CH_3	10.2, CH_3	10.6, CH_3

Figure S1. ^{1}H NMR spectrum of compound **1** ($CDCl_3$, 600 MHz)

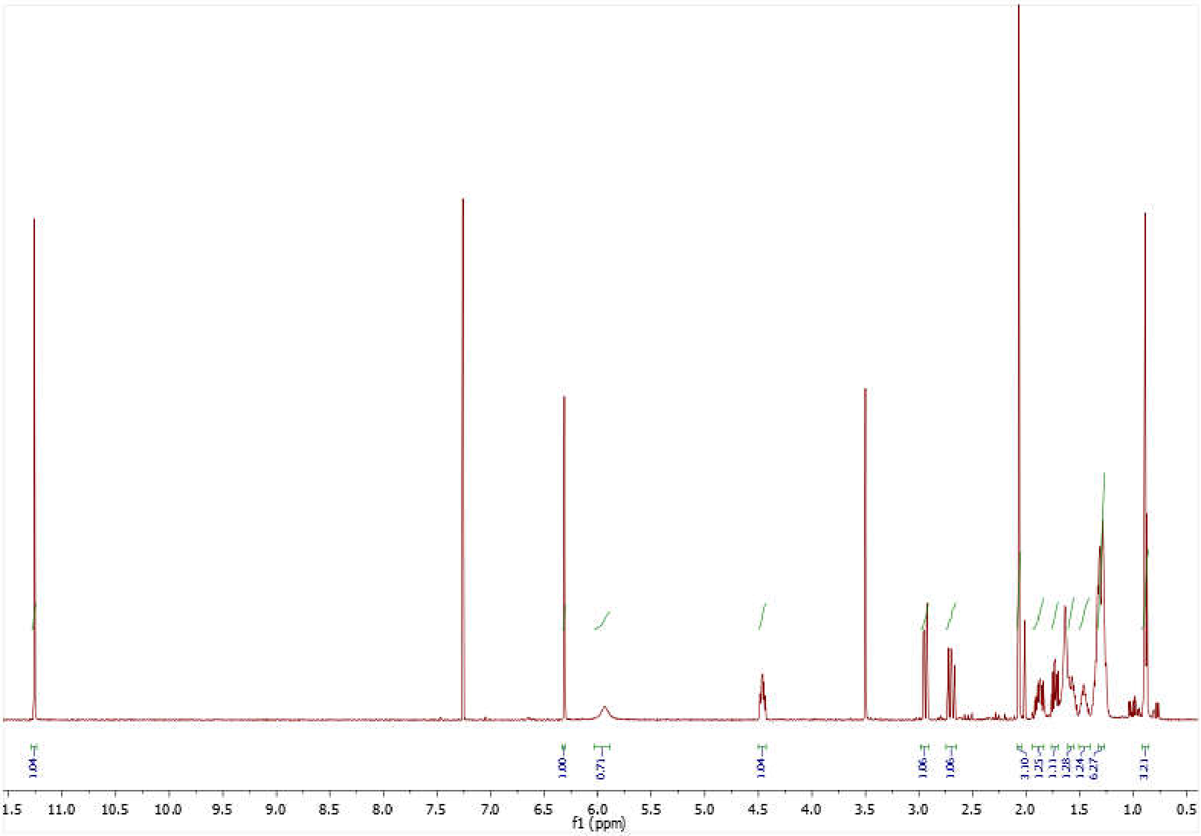

Figure S2. ^{13}C NMR spectrum of compound **1** ($CDCl_3$, 150 MHz)

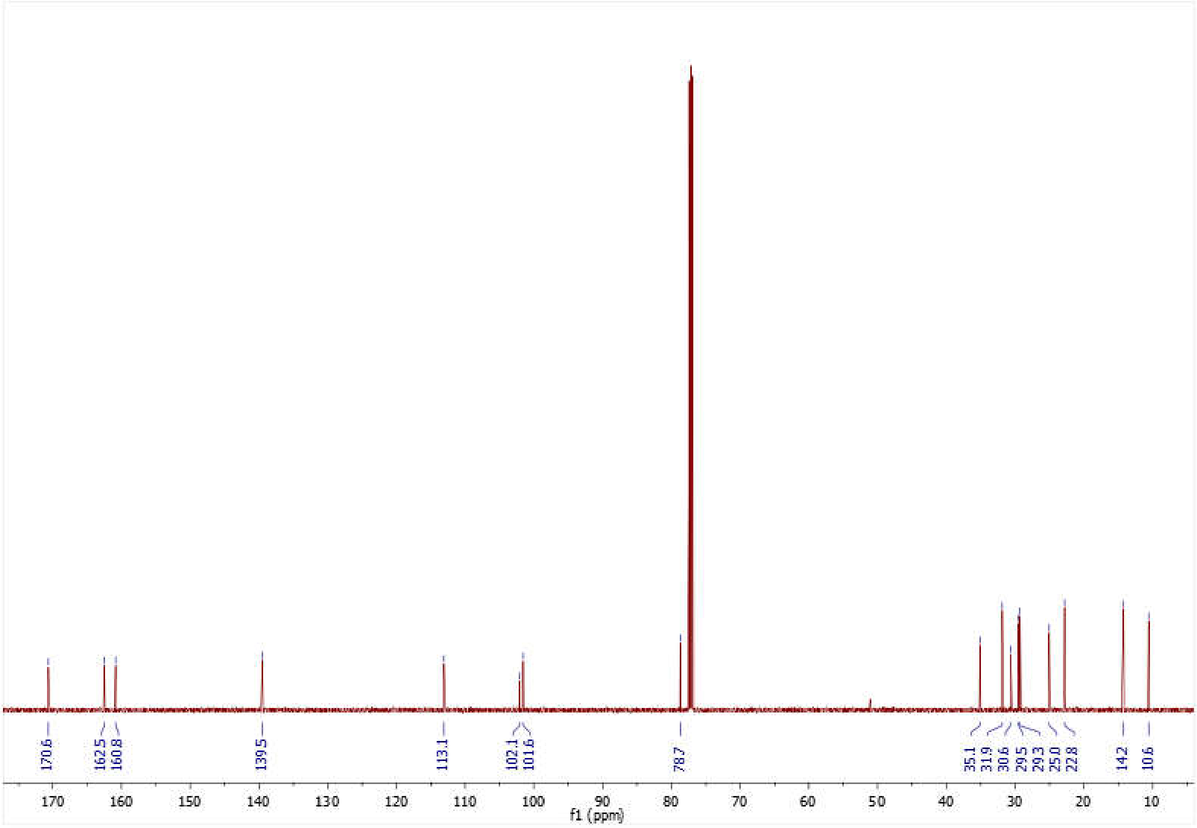

Figure S3. COSY spectrum of compound **1** ($CDCl_3$, 600 MHz)

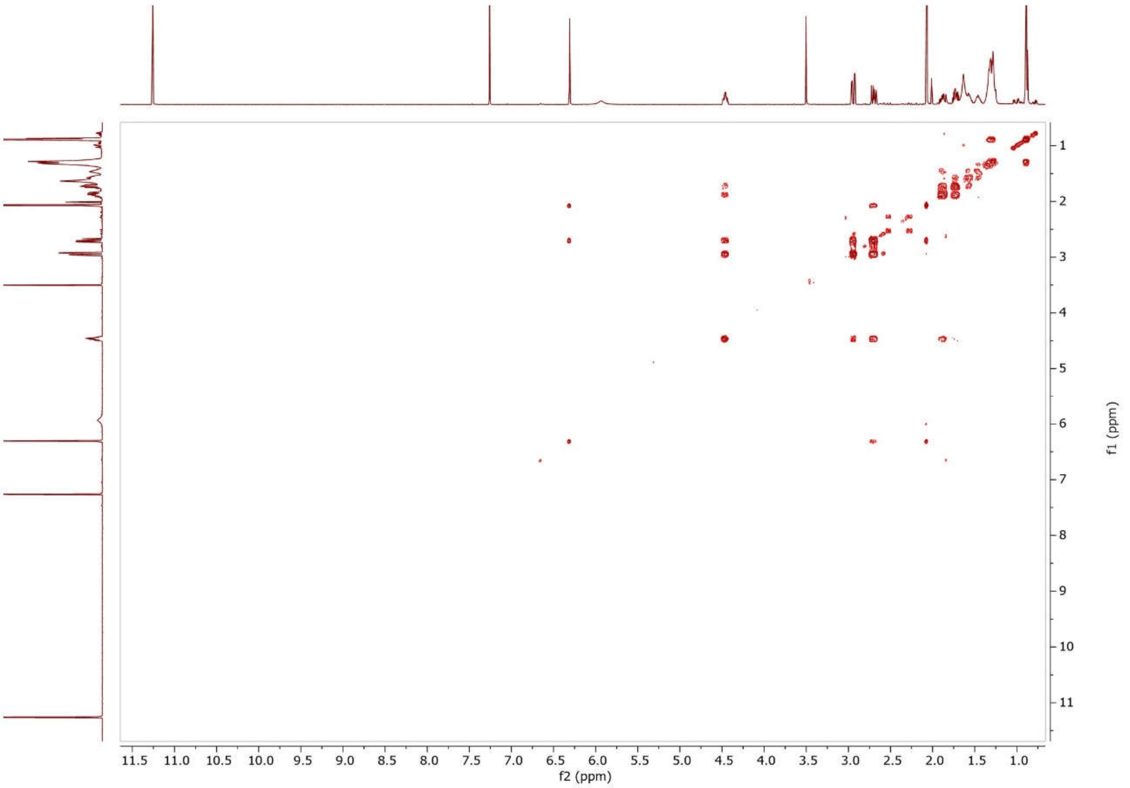

Figure S4. HSQC spectrum of compound **1** ($CDCl_3$, 600/150 MHz)

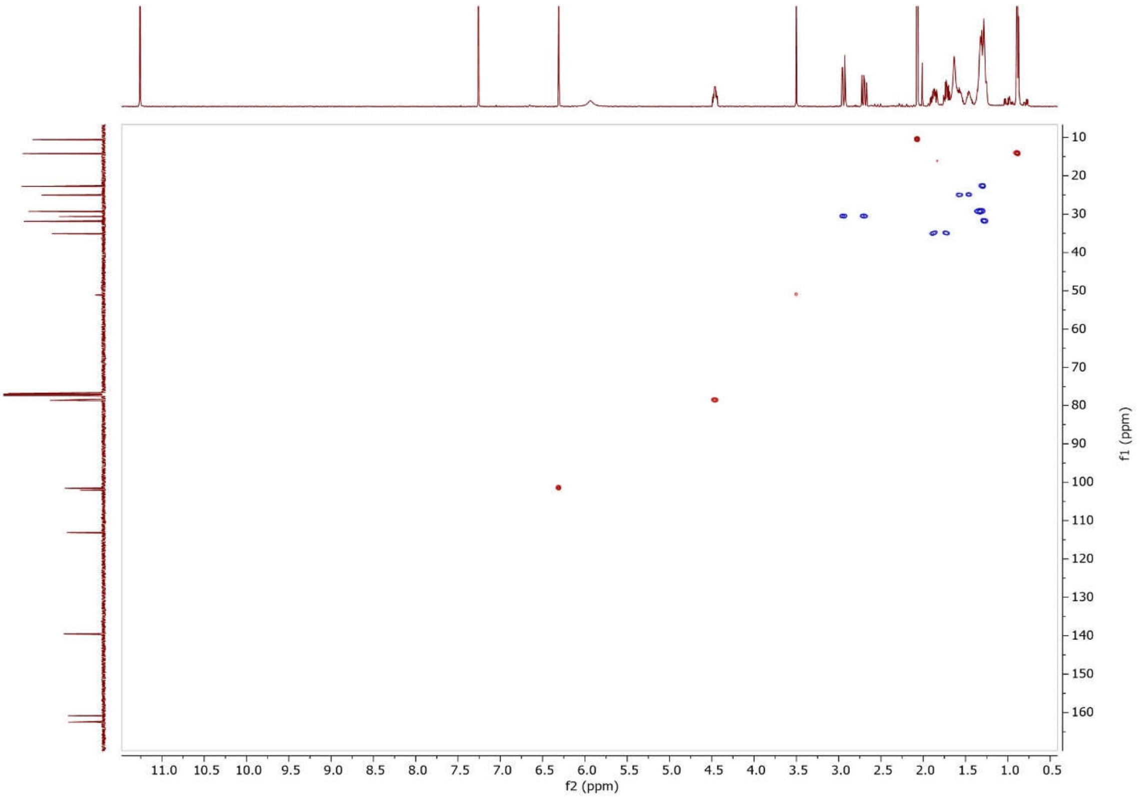

Figure S5. HMBC spectrum of compound **1** ($CDCl_3$, 600/150 MHz)

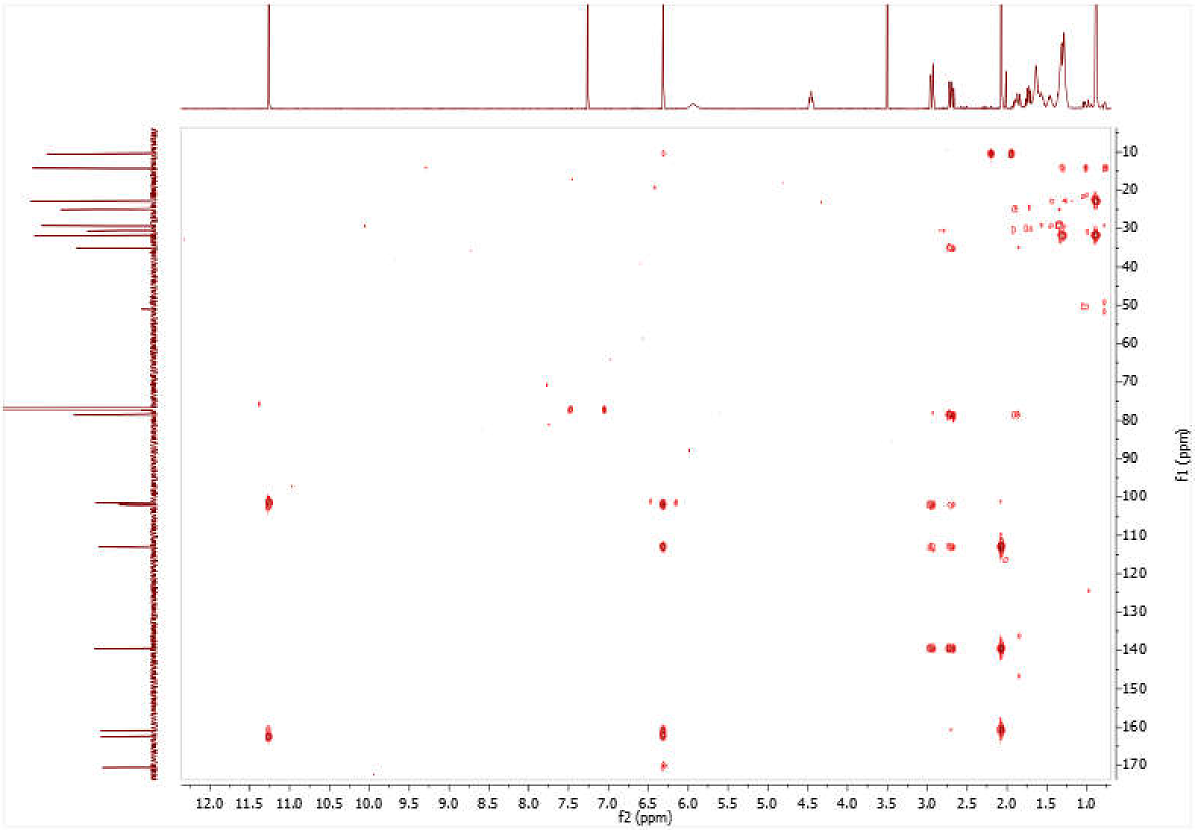

Figure S6. TOCSY spectrum of compound **1** ($CDCl_3$, 600 MHz)

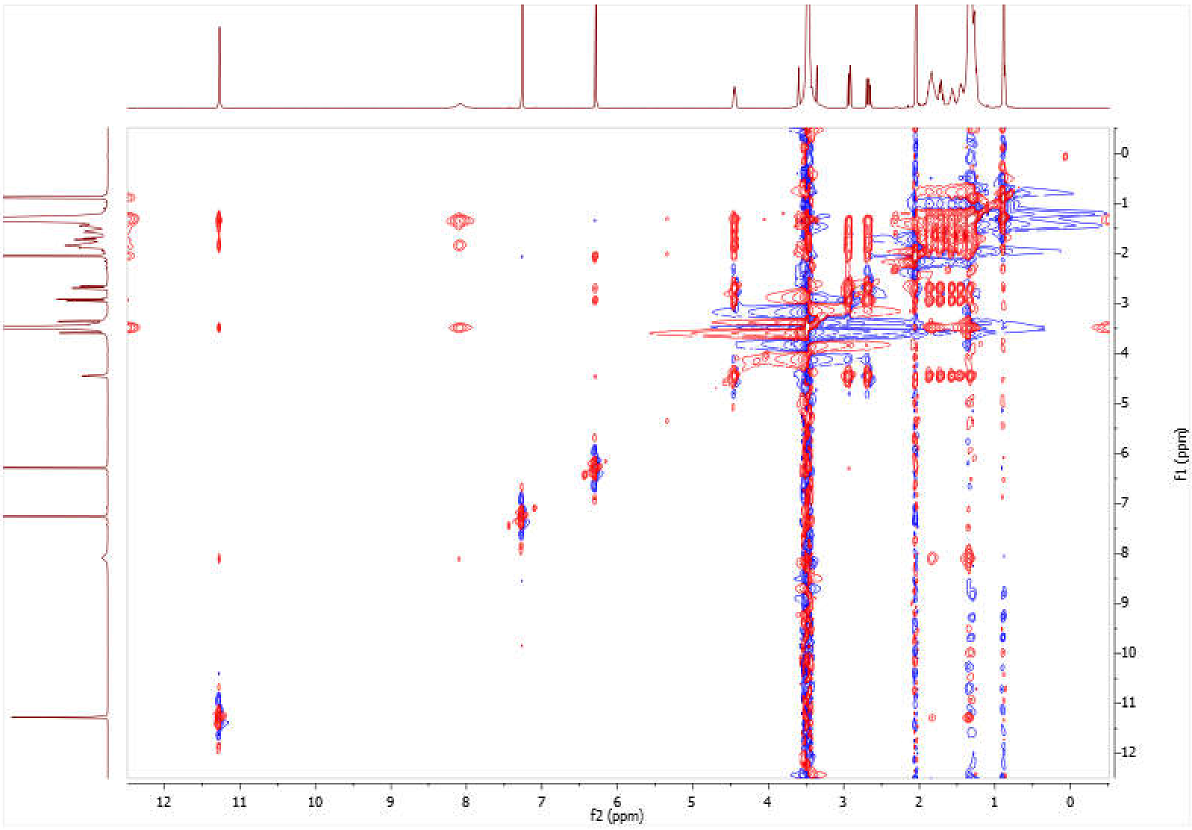

Figure S7. NOESY spectrum of compound **1** ($CDCl_3$, 600 MHz)

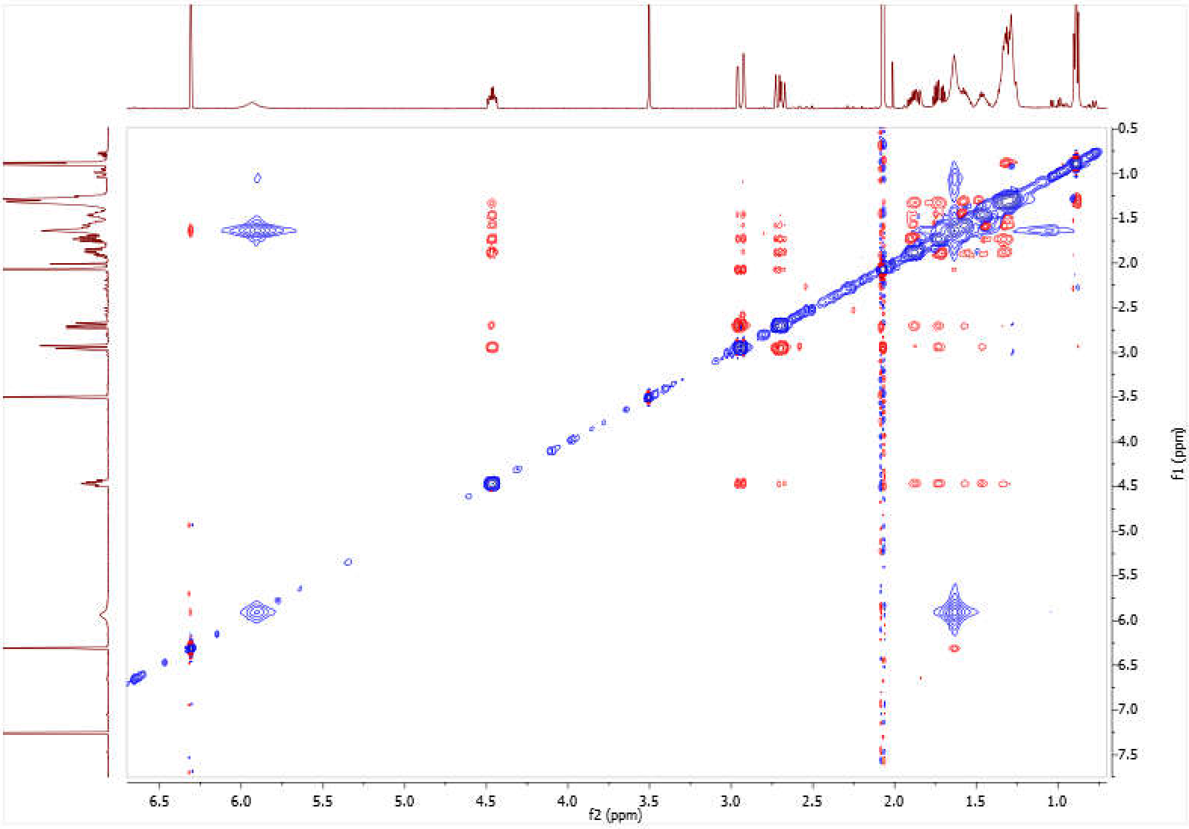

Figure S8. ^{1}H NMR spectrum of compound **1** (CD_3OD, 500 MHz)

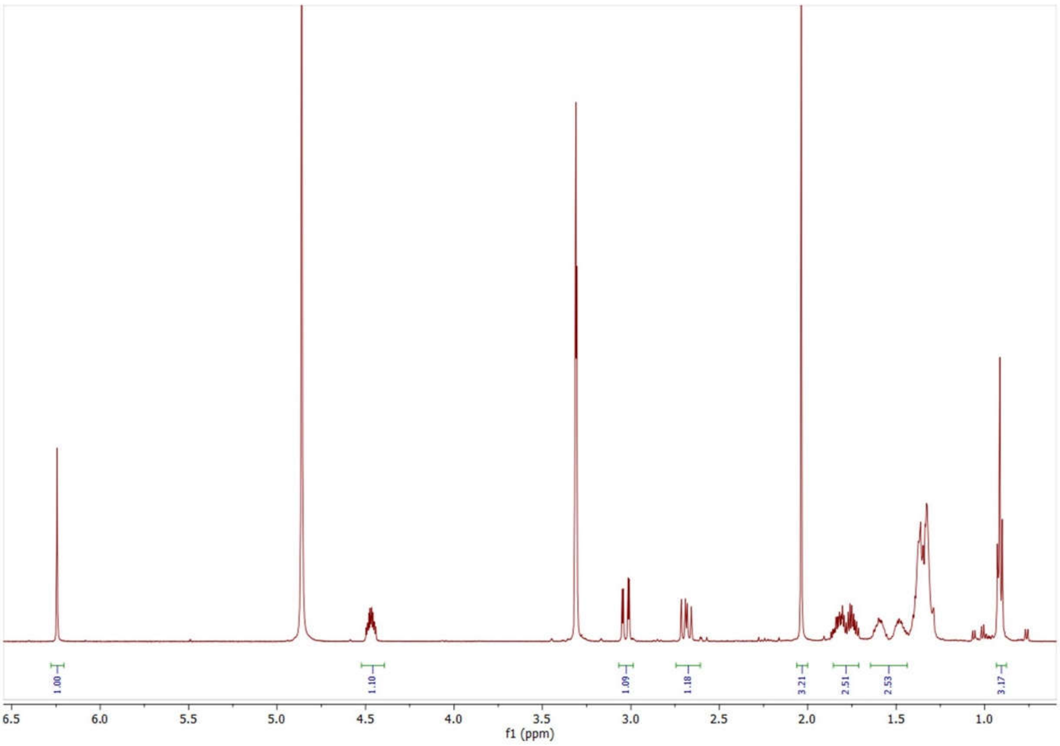

Figure S9. HR-ESIMS spectrum of compound **1**

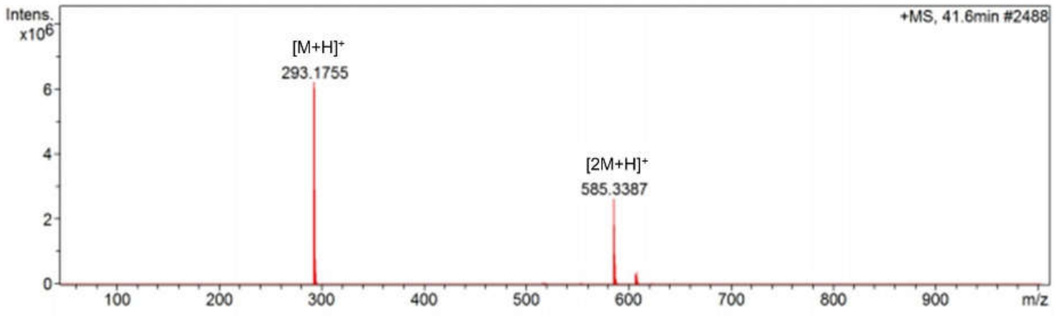

Figure S10. HR-ESIMS/MS spectrum of compound **1**

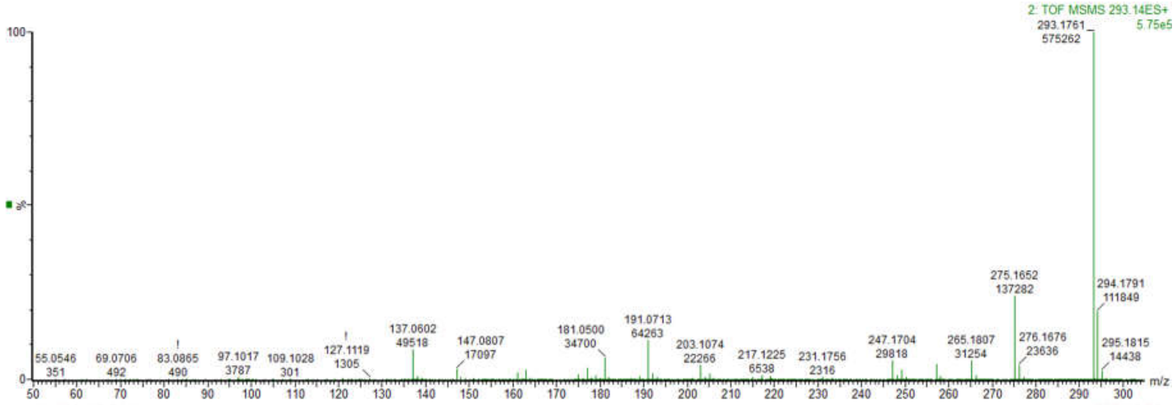

Figure S11. FT-IR spectrum of compound **1**

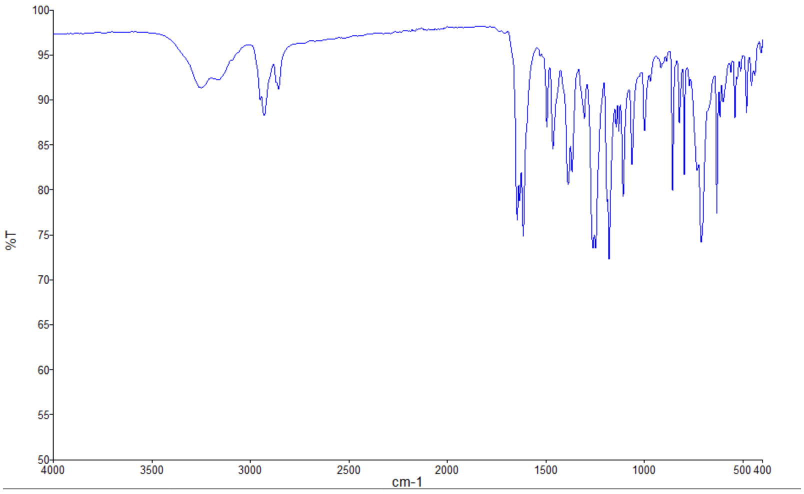

Figure S12. ^{1}H NMR spectrum of compound **2** ($CDCl_3$, 600 MHz)

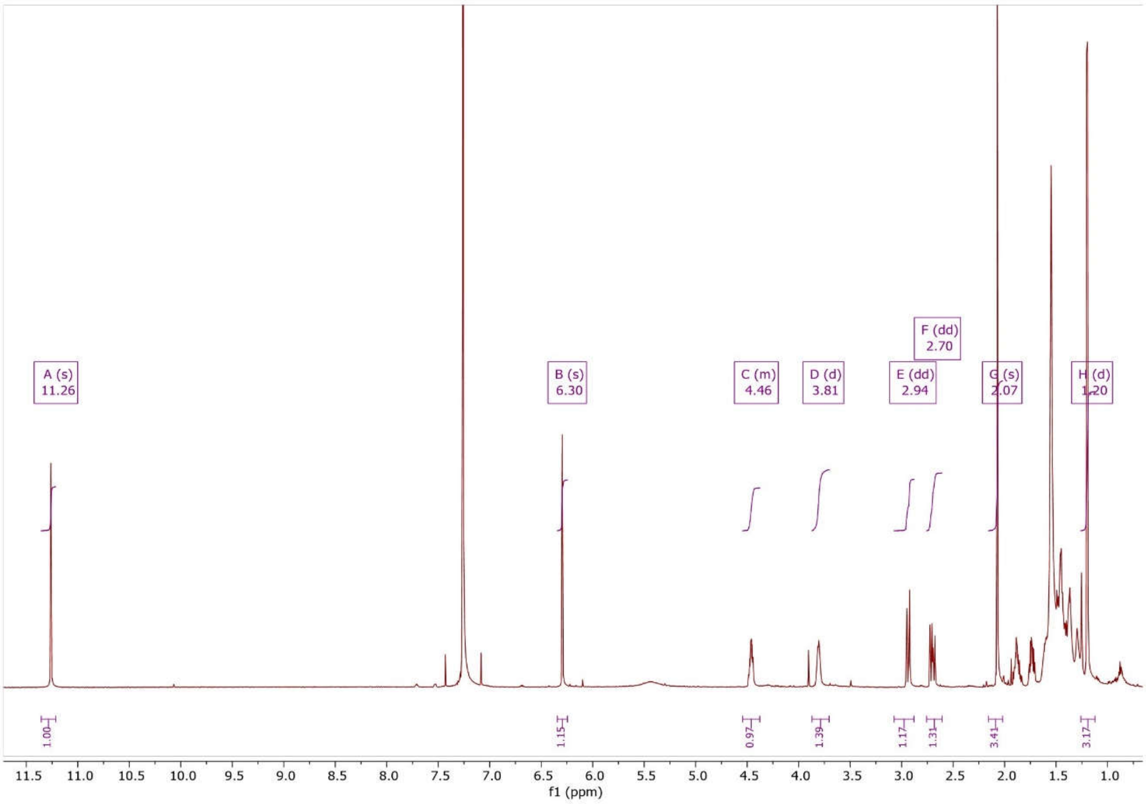

Figure S13. ^{13}C spectrum of compound **2** ($CDCl_3$, 150 MHz)

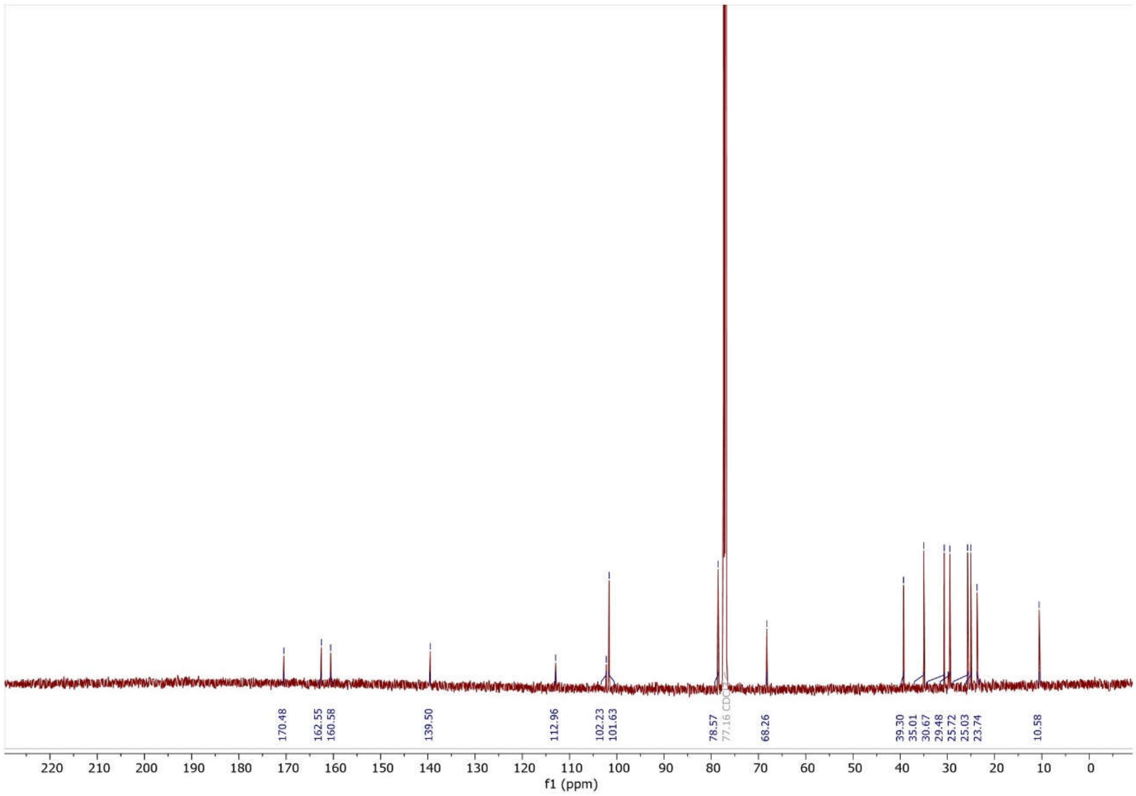

Figure S14. COSY spectrum of compound **2** ($CDCl_3$, 600 MHz)

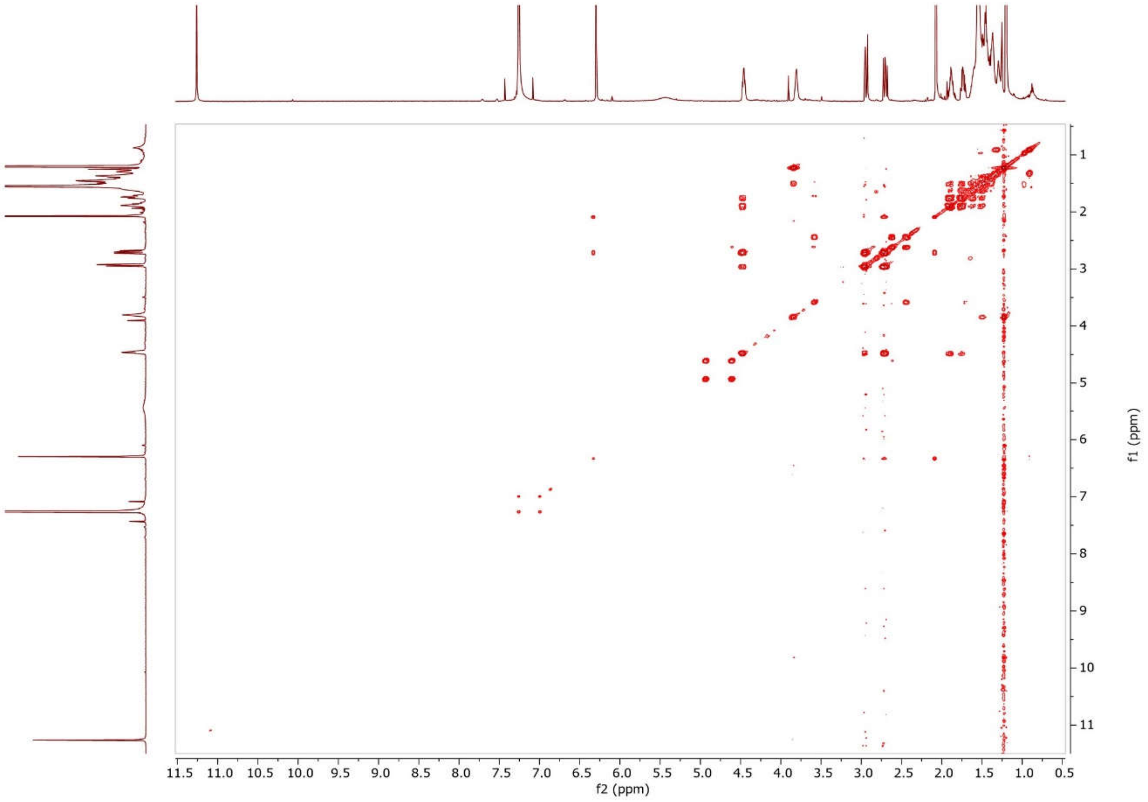

Figure S15. HSQC spectrum of compound **2** ($CDCl_3$, 600/150 MHz)

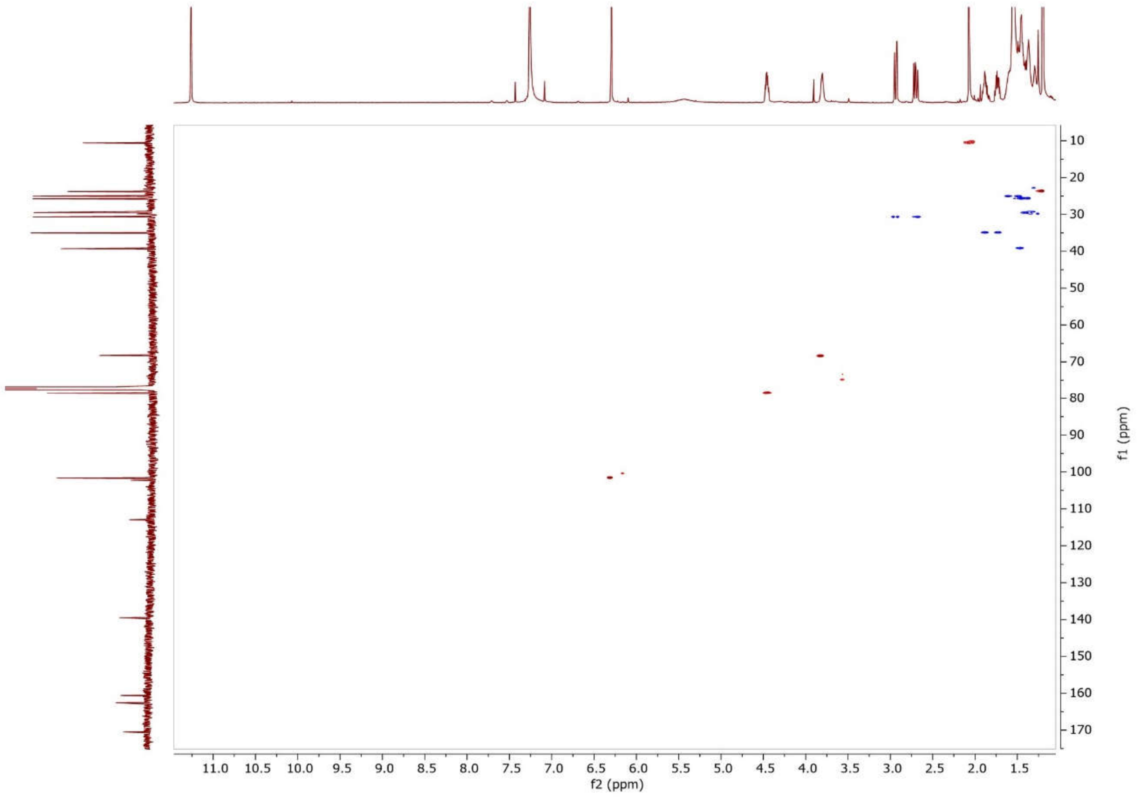

Figure S16. HMBC spectrum of compound **2** ($CDCl_3$, 600/150 MHz)

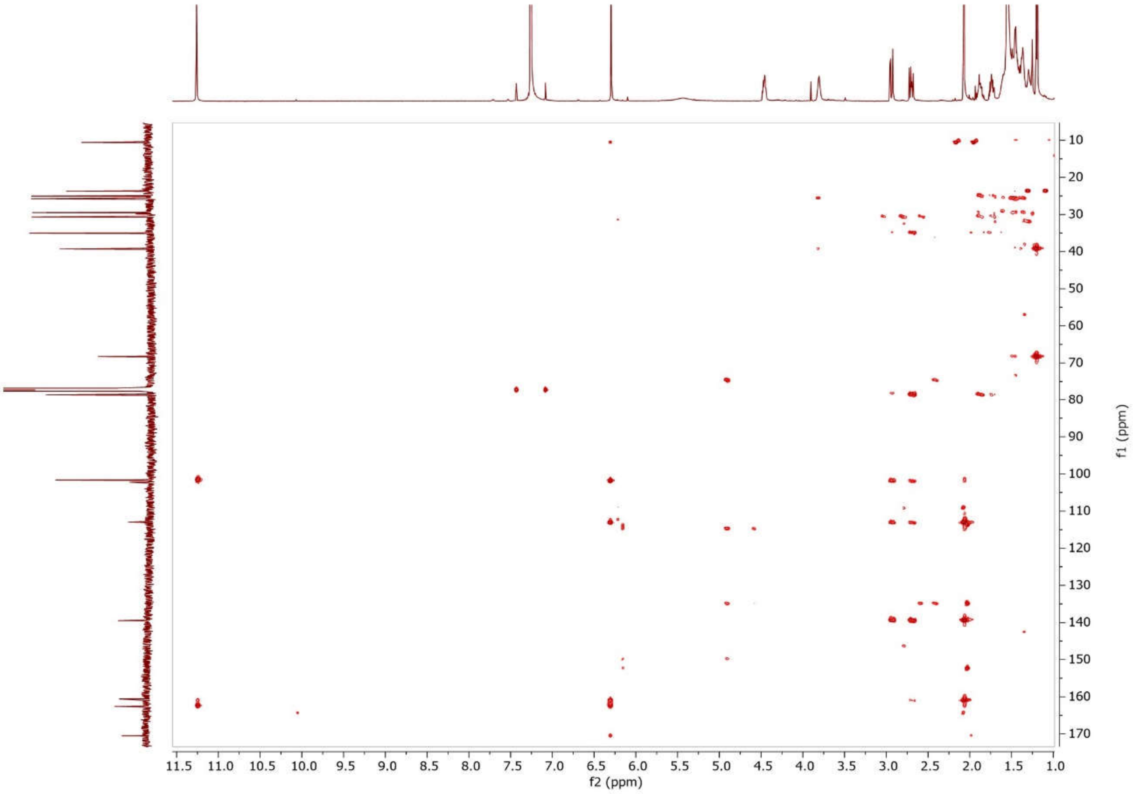

Figure S17. NOESY spectrum of compound **2** ($CDCl_3$, 600 MHz)

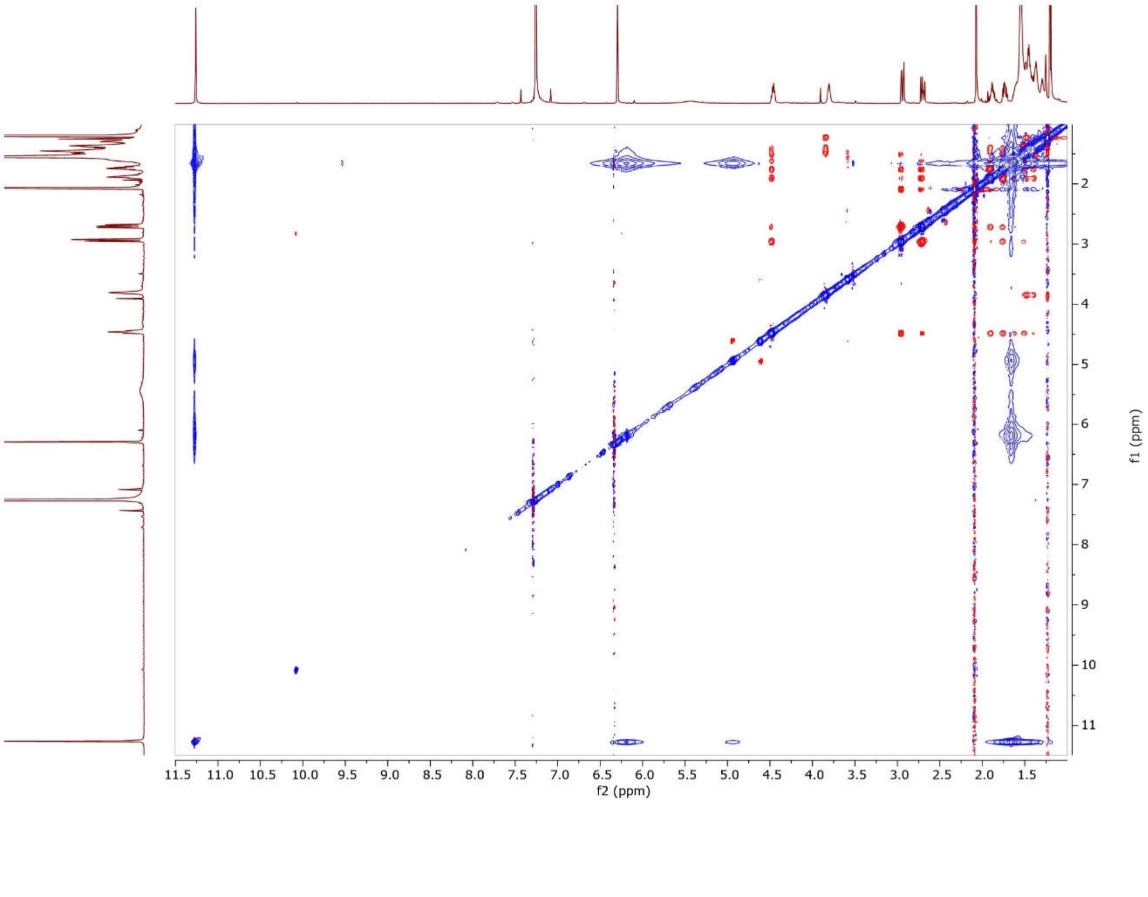

Figure S18. HR-ESIMS spectrum of compound **2**

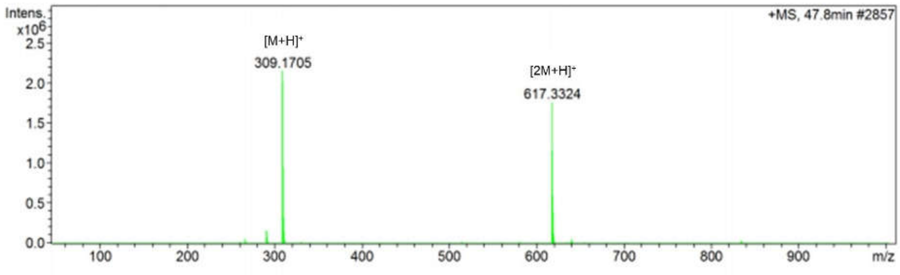

Figure S19. HR-ESIMS/MS spectrum of compound **2**

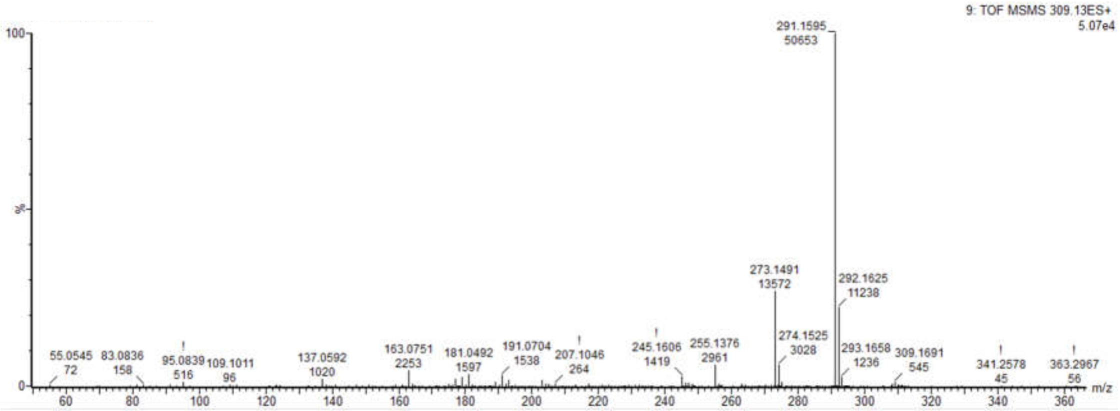

Figure S20. FT-IR spectrum of compound **2**

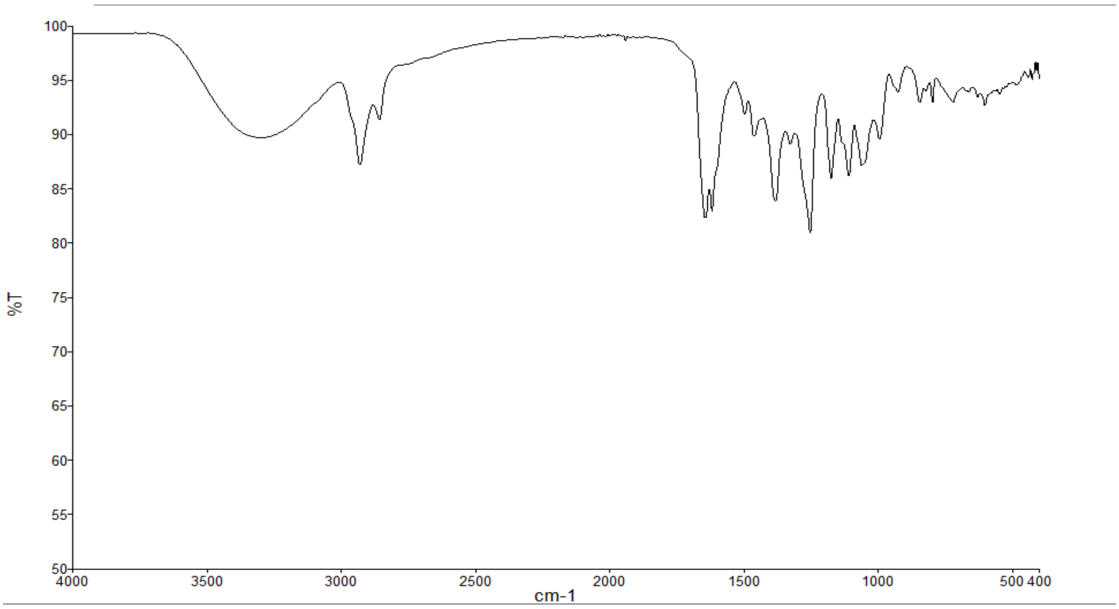

Figure S21. ^{1}H NMR spectrum of compound **3** ($CDCl_3$, 600 MHz)

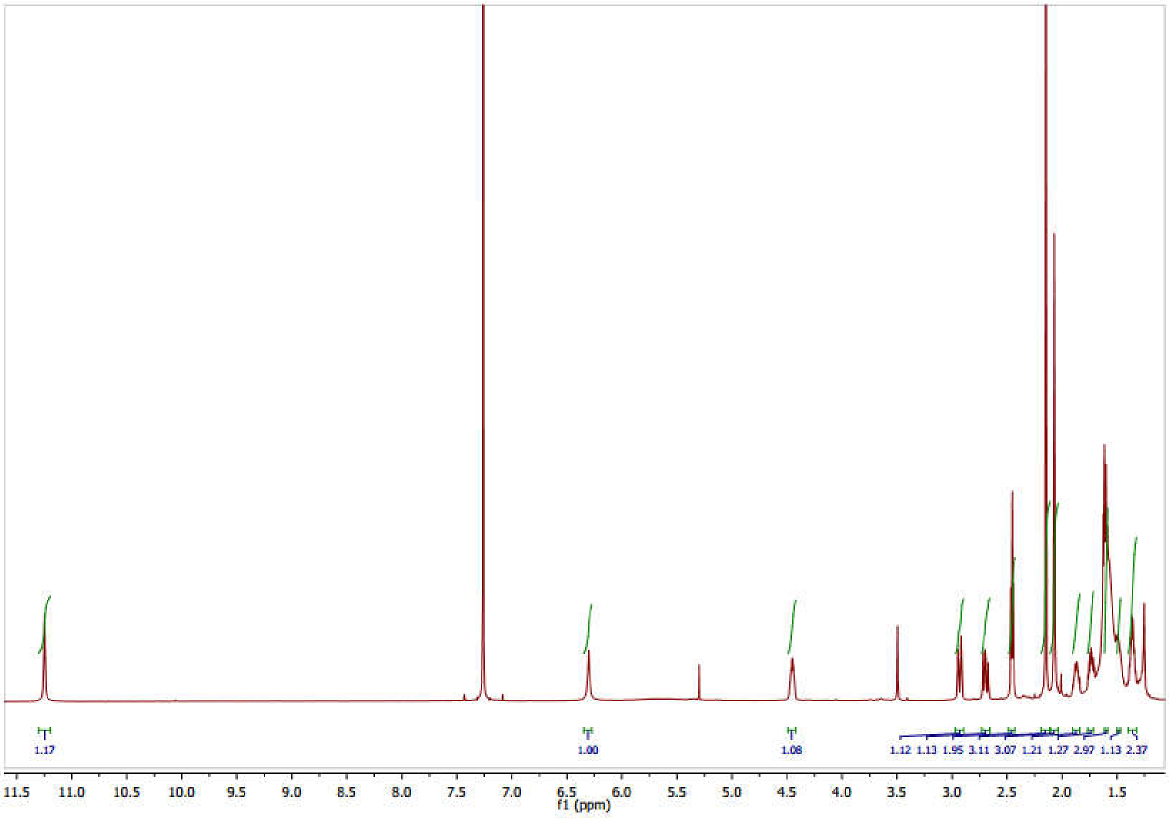

Figure S22. ^{13}C NMR spectrum of compound **3** ($CDCl_3$, 150 MHz)

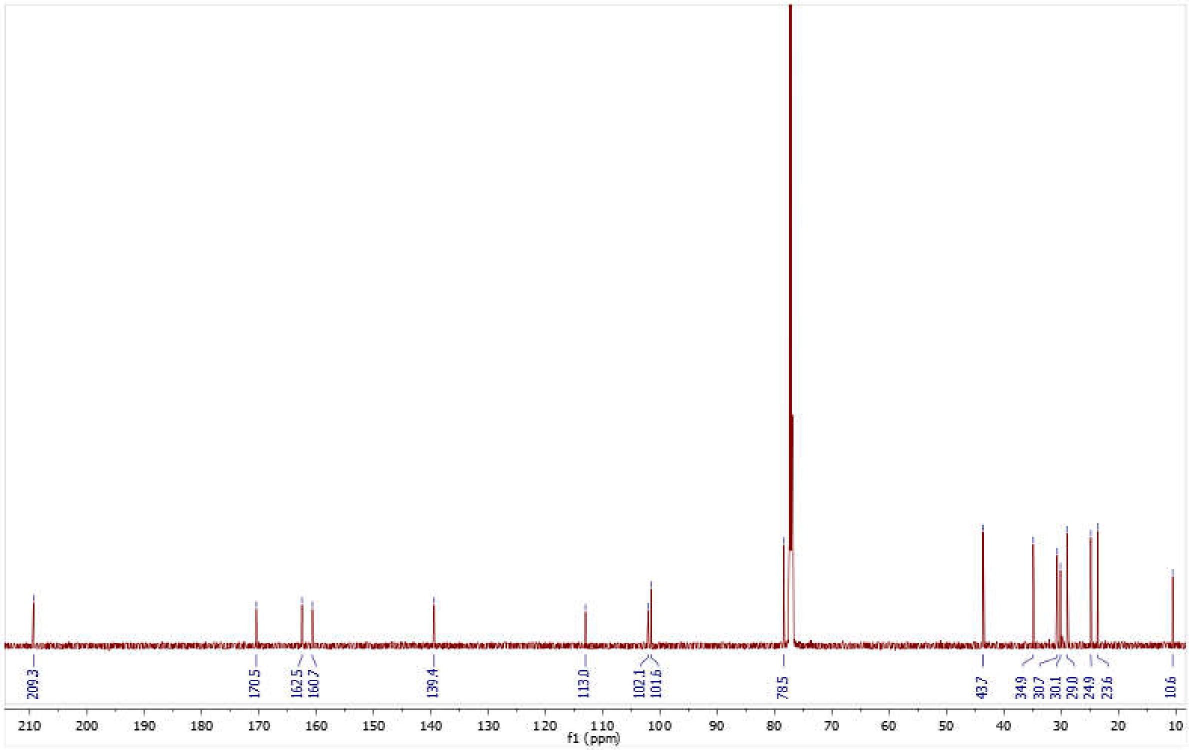

Figure S23. COSY spectrum of compound **3** ($CDCl_3$, 600 MHz)

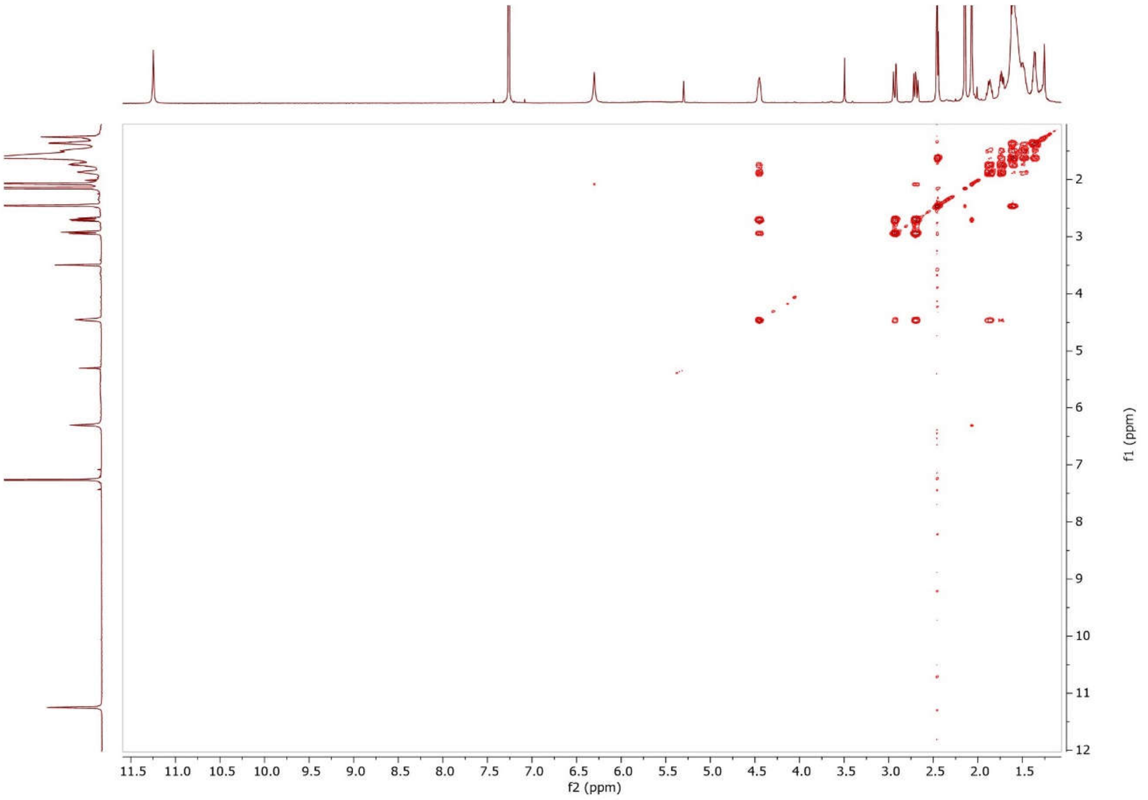

Figure S24. HSQC spectrum of compound **3** ($CDCl_3$, 600/150 MHz)

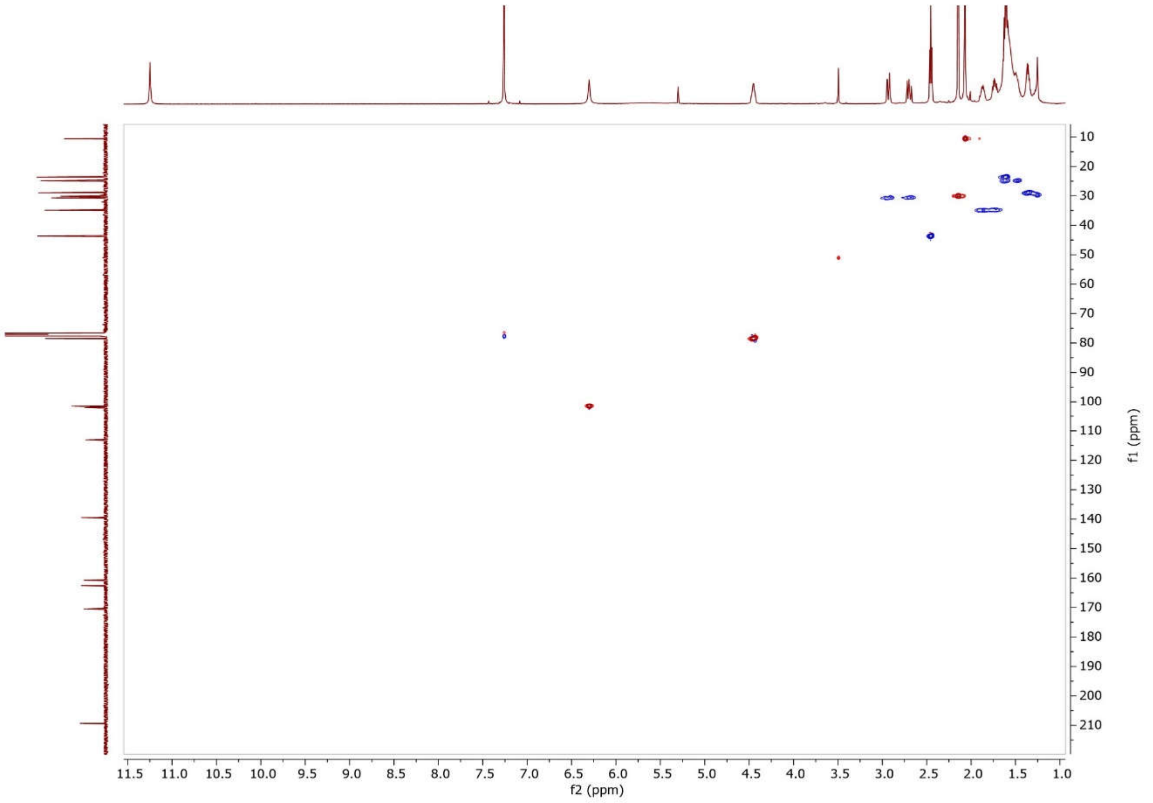

Figure S25. HMBC spectrum of compound **3** ($CDCl_3$, 600/150 MHz)

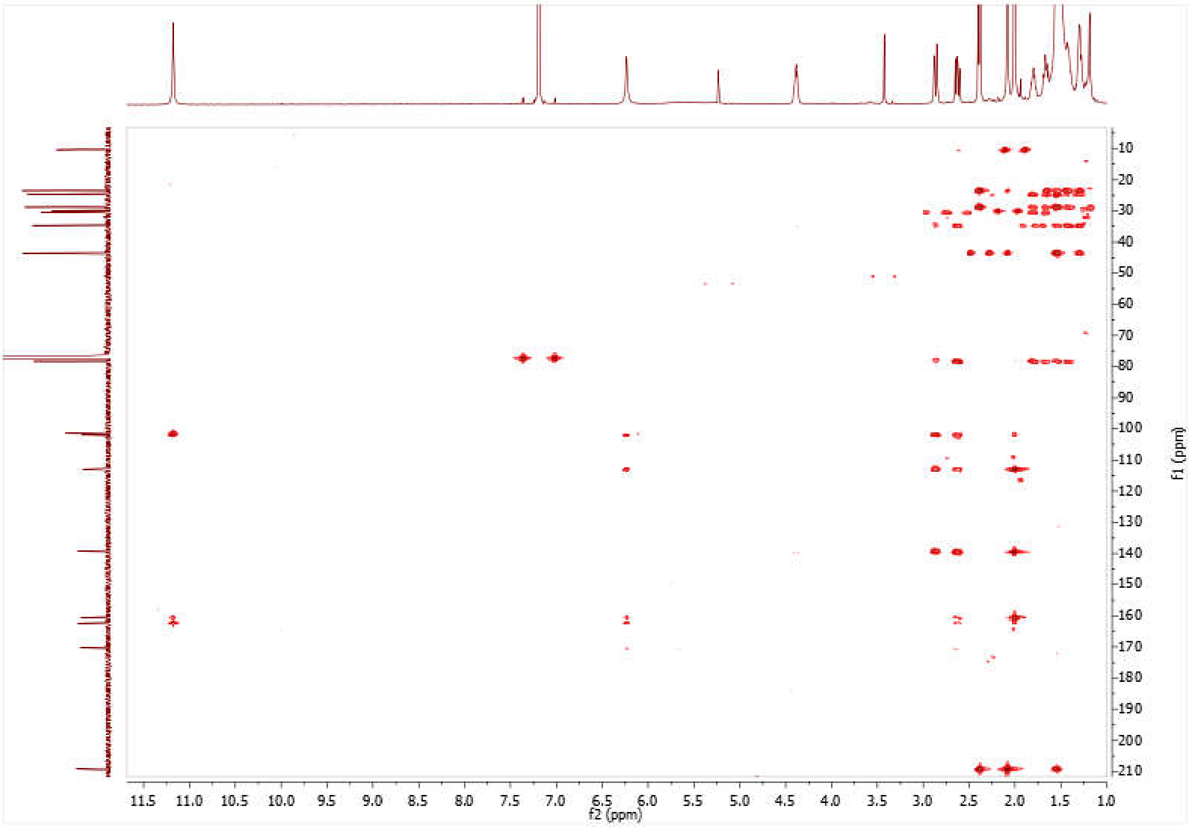

Figure S26. NOESY spectrum of compound **3** ($CDCl_3$, 600 MHz)

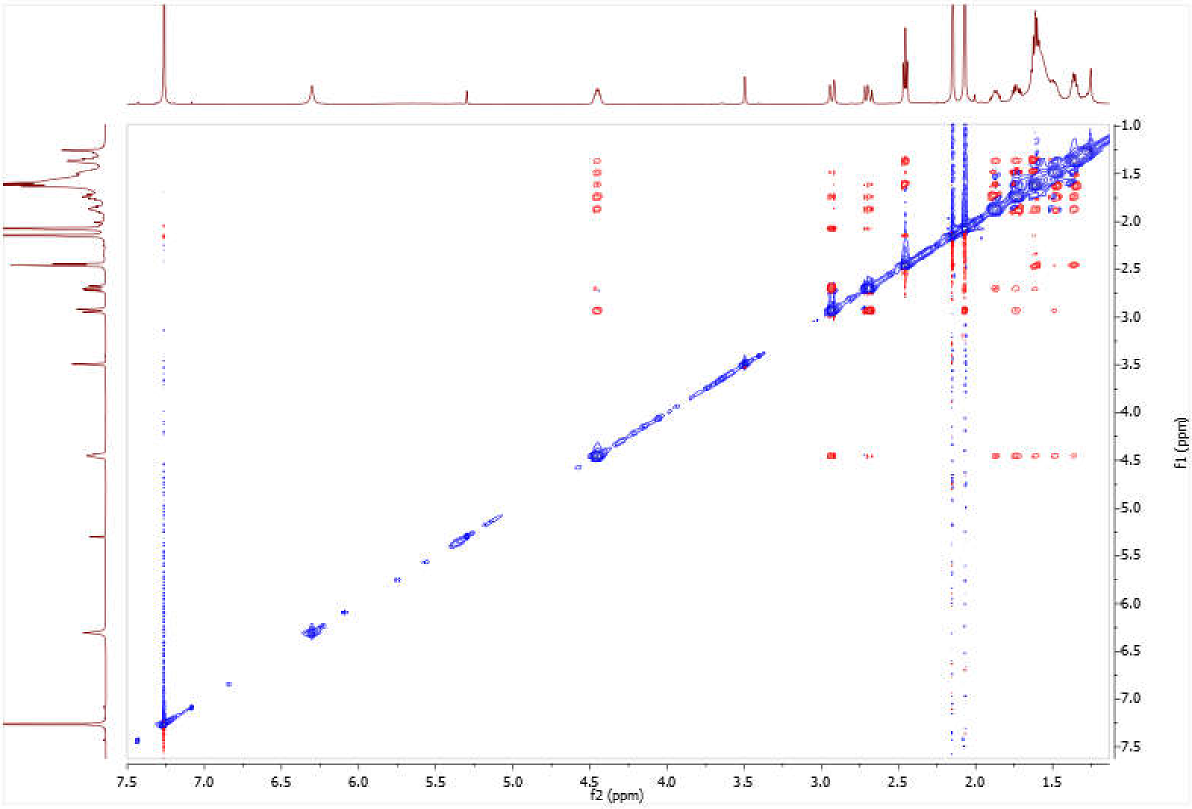

Figure S27. HR-ESIMS spectrum of compound **3**

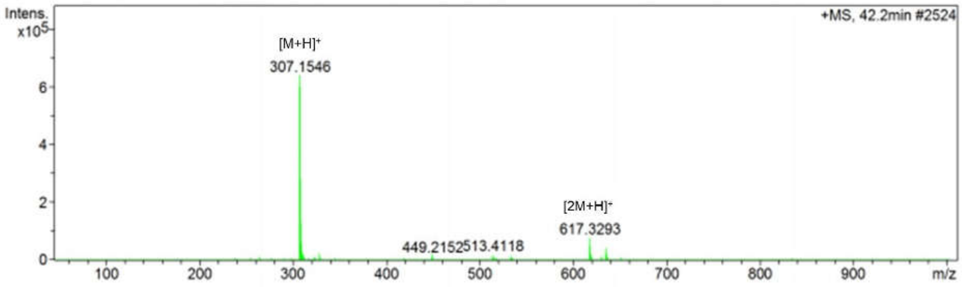

Figure S28. HR-ESIMS/MS spectrum of compound **3**

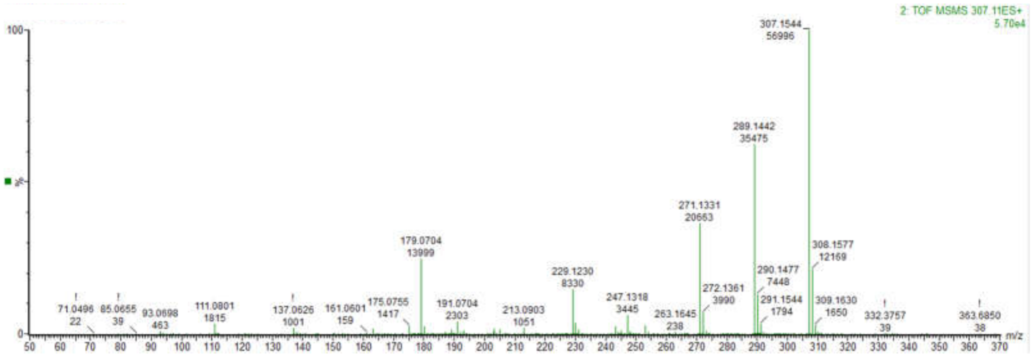

Figure S29. FT-IR spectrum of compound **3**

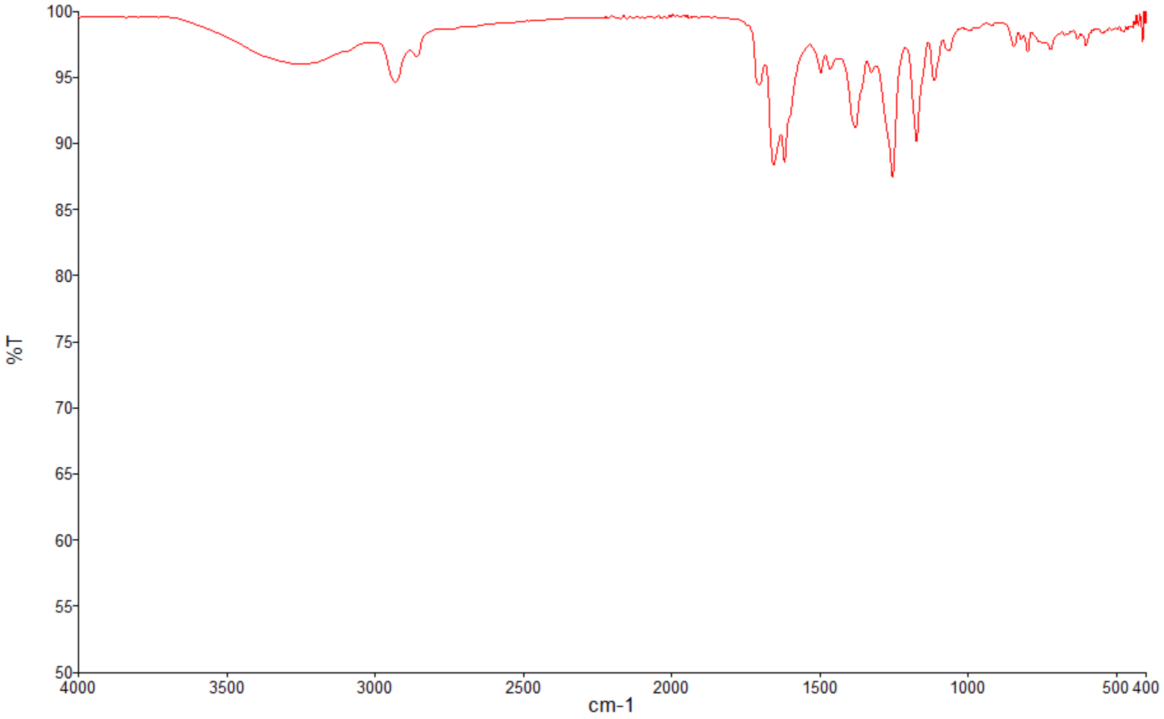

Figure S30. ^{1}H NMR spectrum of compound **4** ($CDCl_3$, 600 MHz)

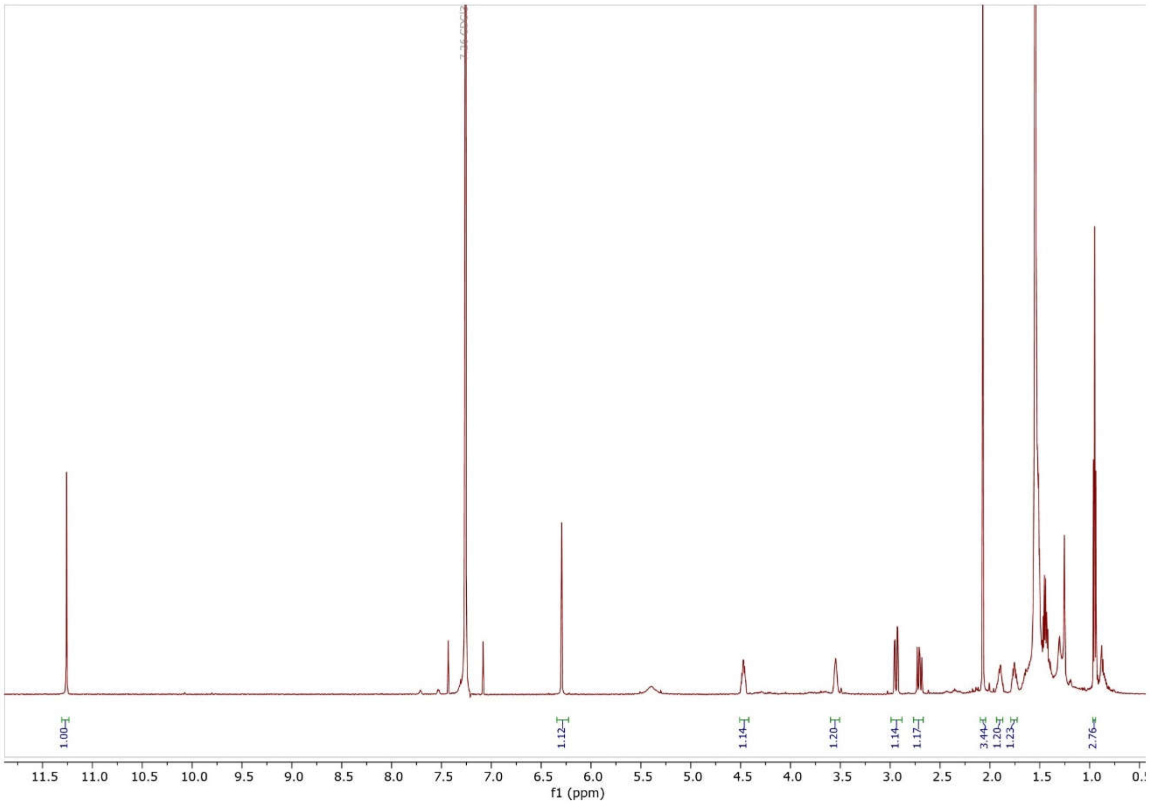

Figure S31. ^{13}C spectrum of compound **4** ($CDCl_3$, 150 MHz)

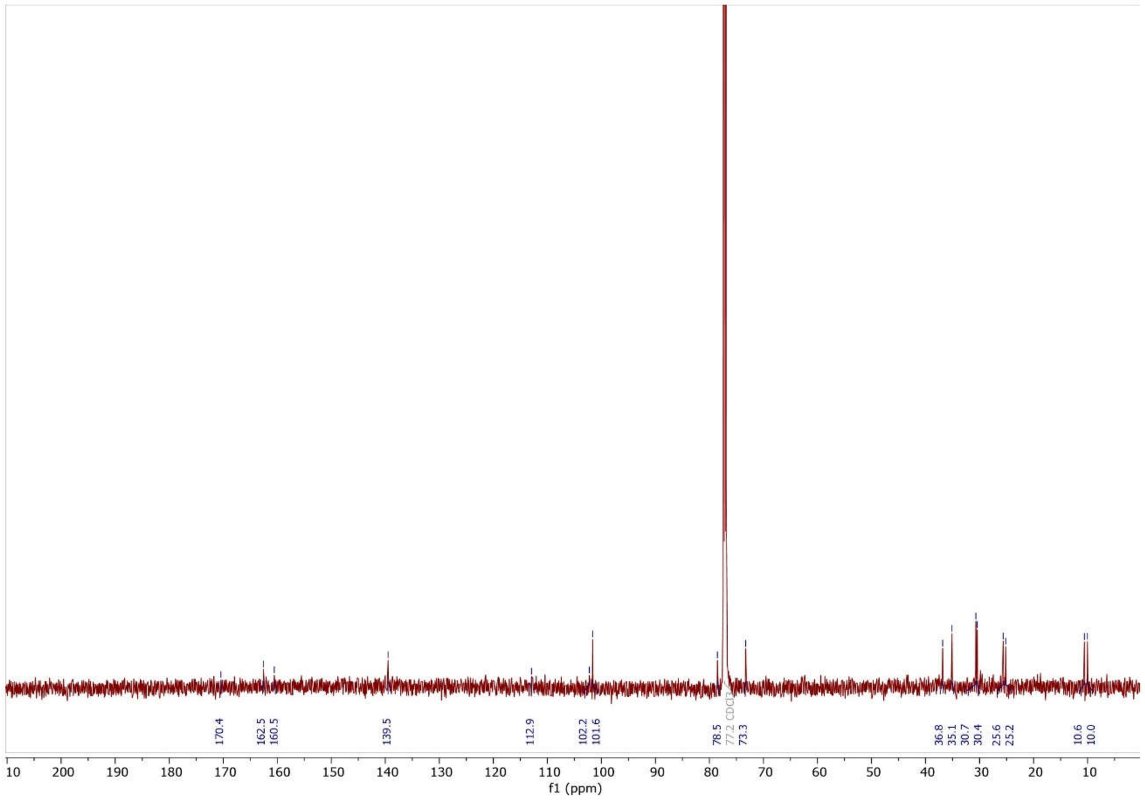

Figure S32. COSY spectrum of compound **4** ($CDCl_3$, 600 MHz)

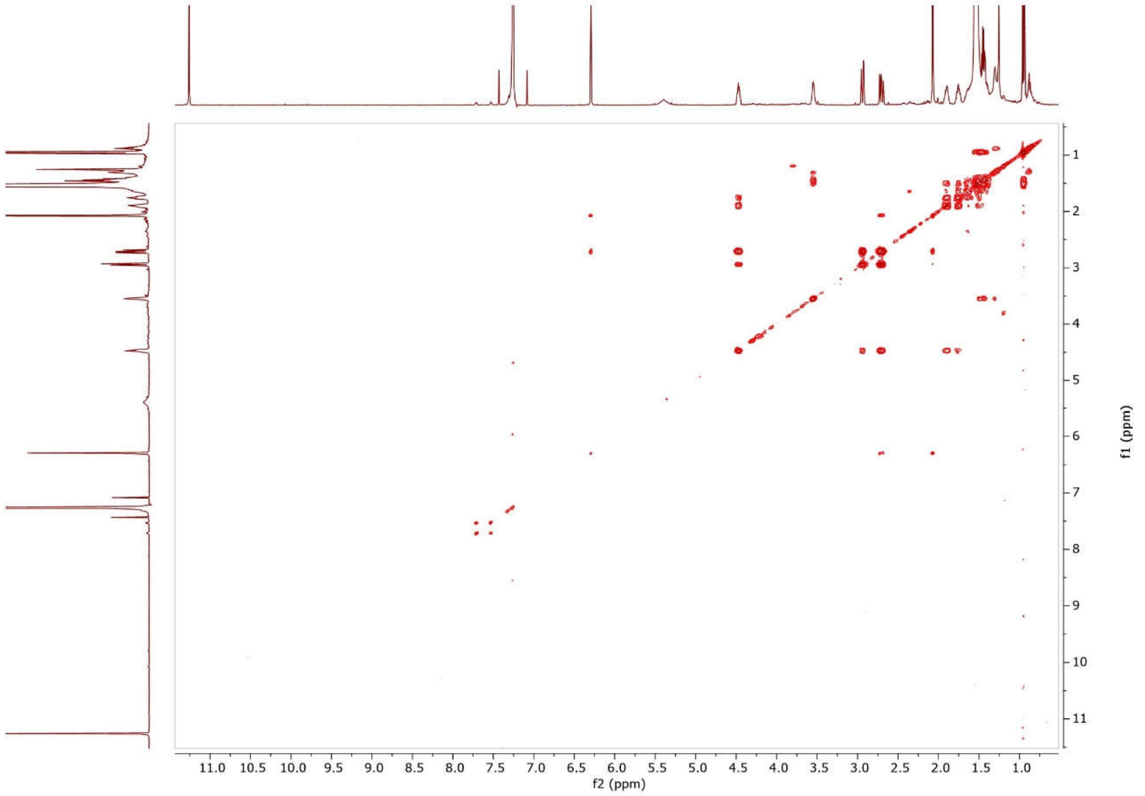

Figure S33. HSQC spectrum of compound **4** ($CDCl_3$, 600/150 MHz)

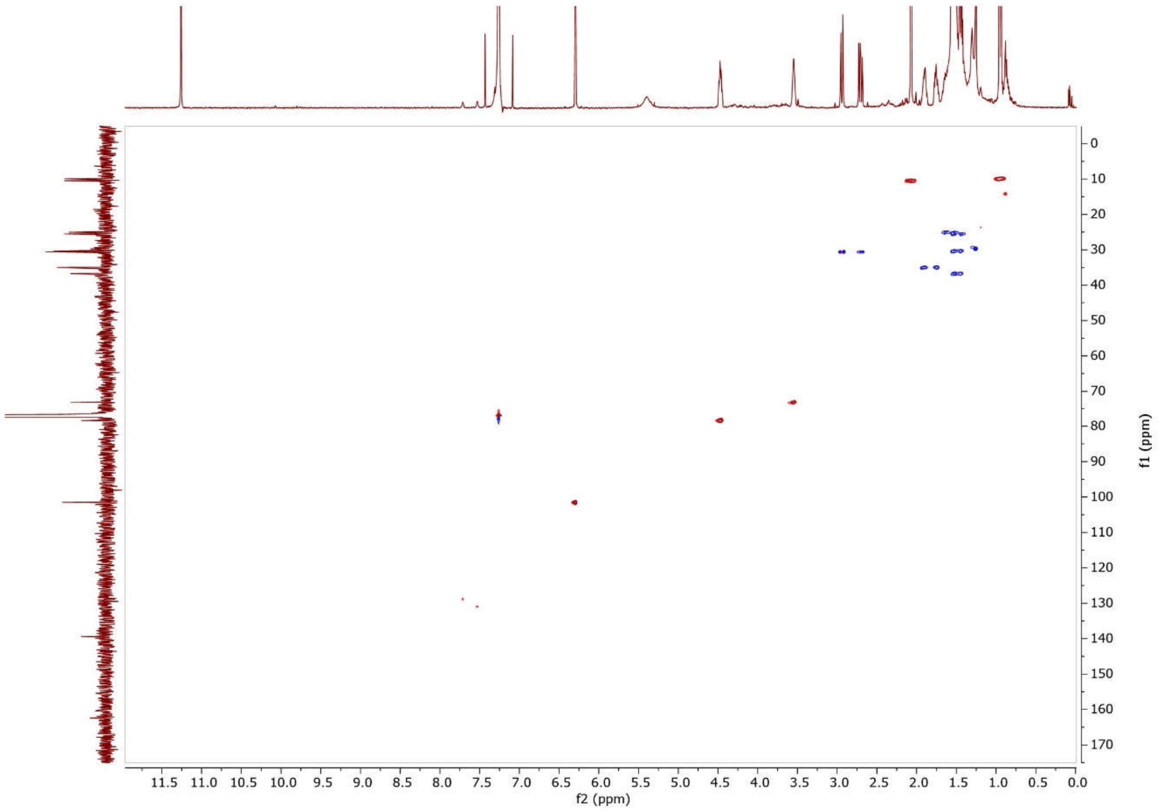

Figure S34. HMBC spectrum of compound **4** ($CDCl_3$, 600/150 MHz)

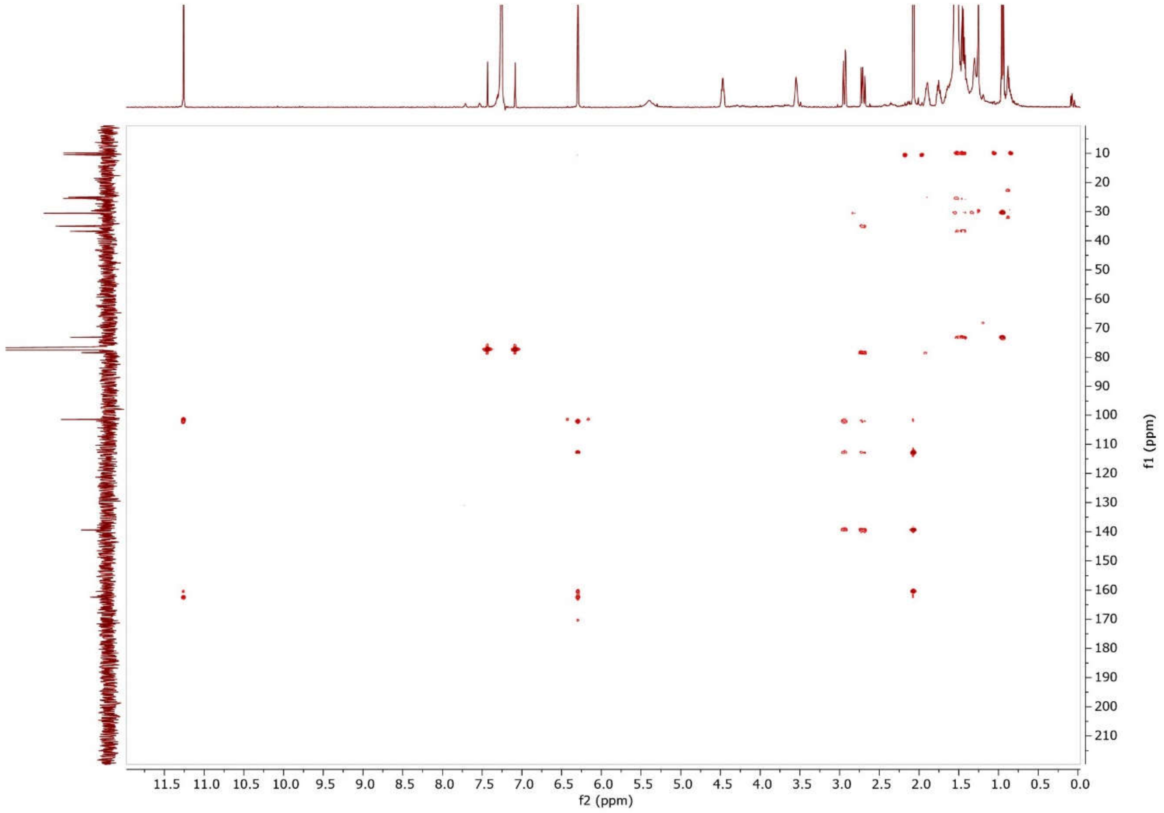

Figure S35. NOESY spectrum of compound **4** ($CDCl_3$, 600 MHz)

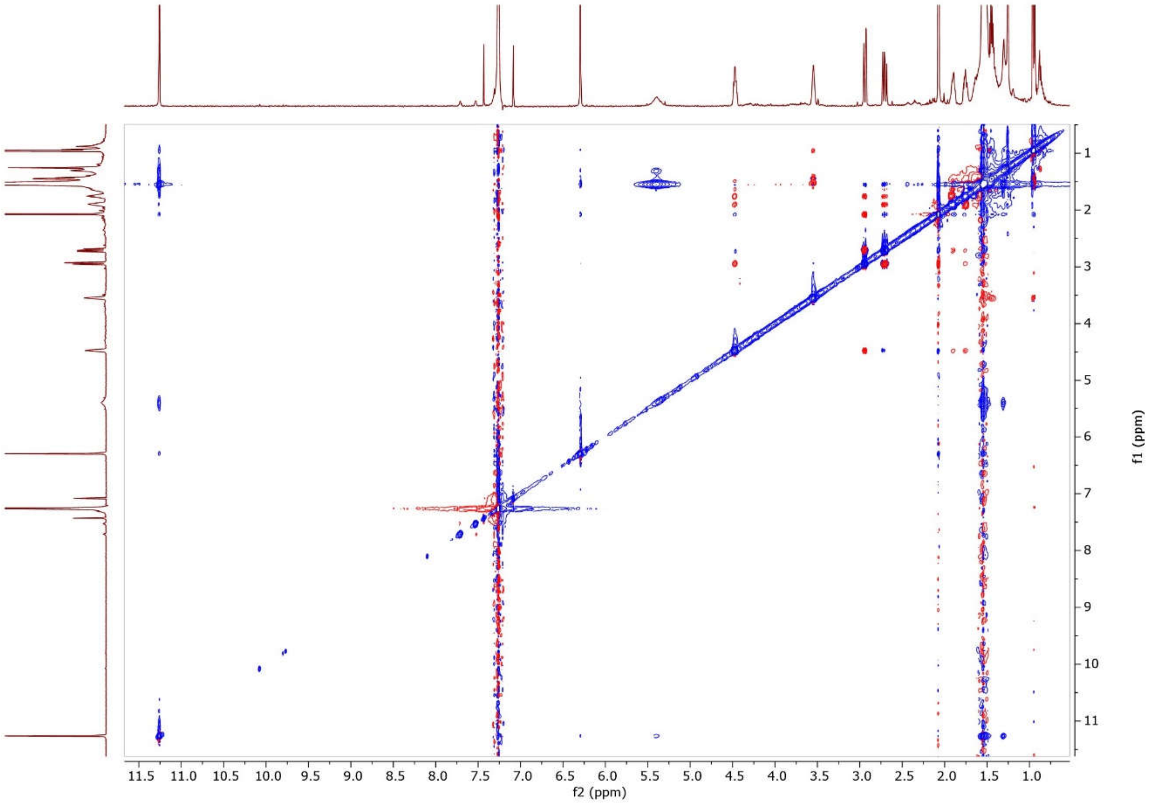

Figure S36. HR-ESIMS spectrum of compound **4**

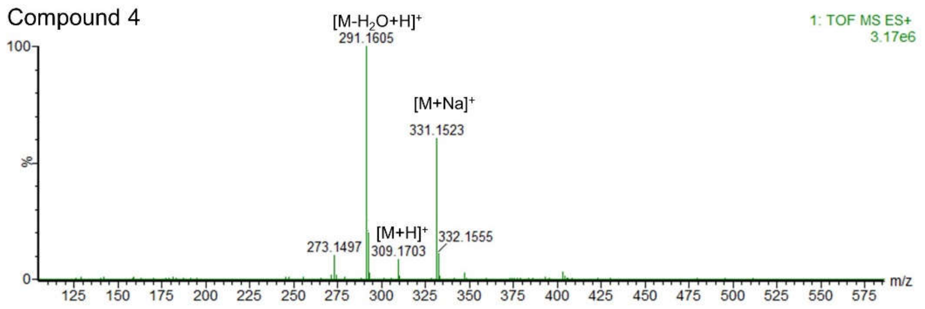

Figure S37. HR-ESIMS/MS spectrum of compound **4**

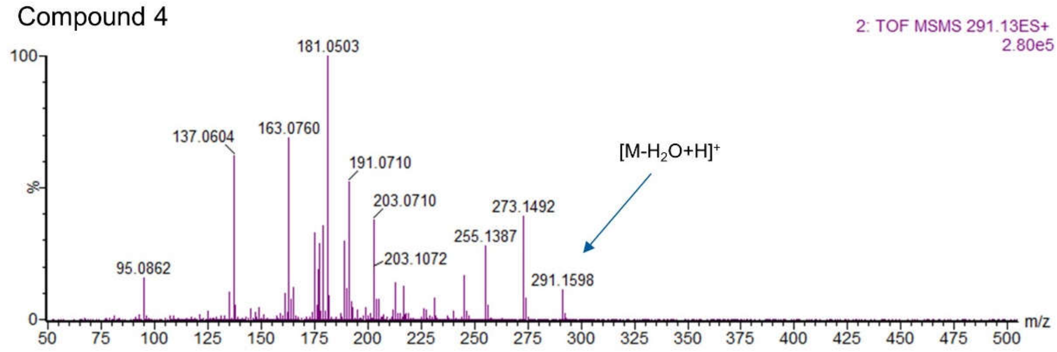

Figure S38. FT-IR spectrum of compound **4**

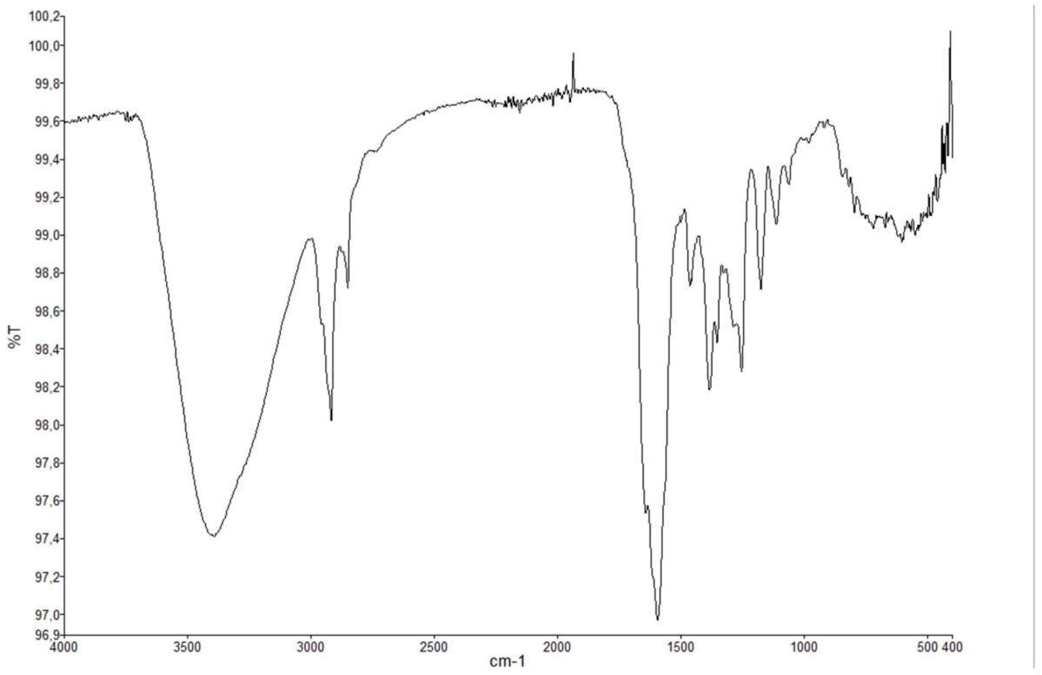

Figure S39. ^{1}H NMR spectrum of compound **5** ($CDCl_3$, 600 MHz)

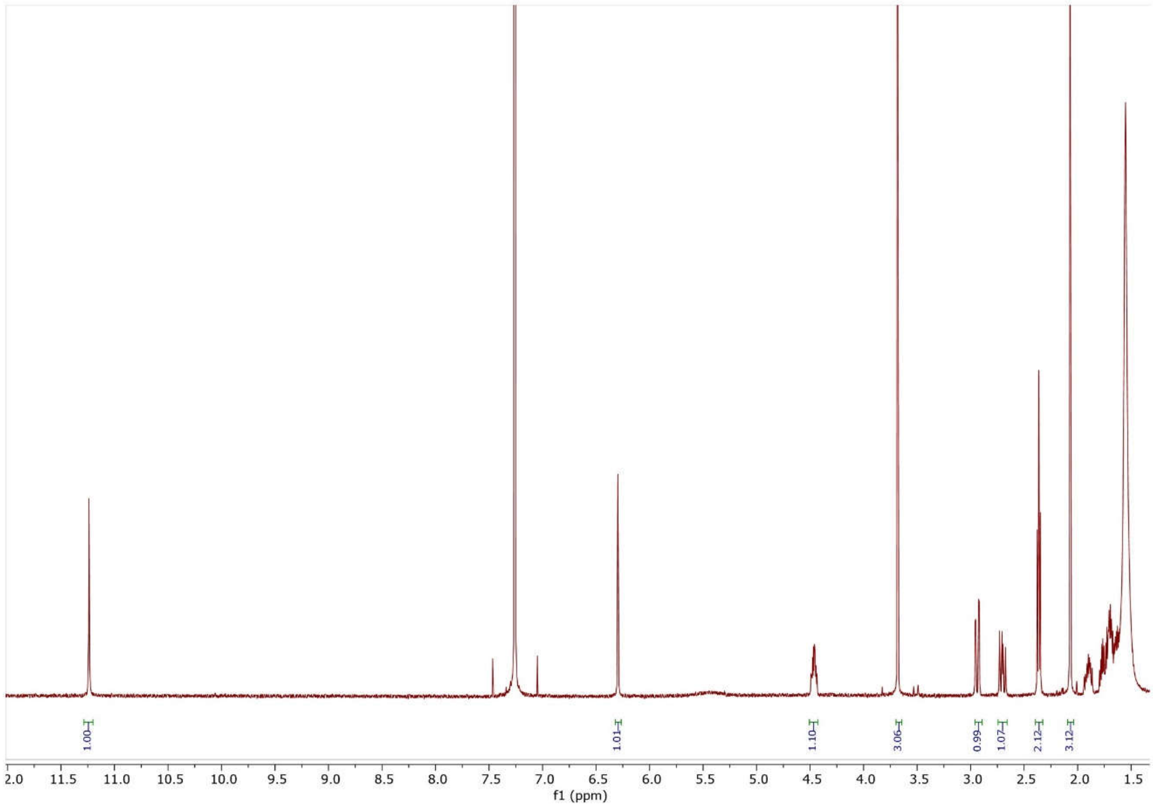

Figure S40. ^{13}C spectrum of compound **5** ($CDCl_3$, 150 MHz)

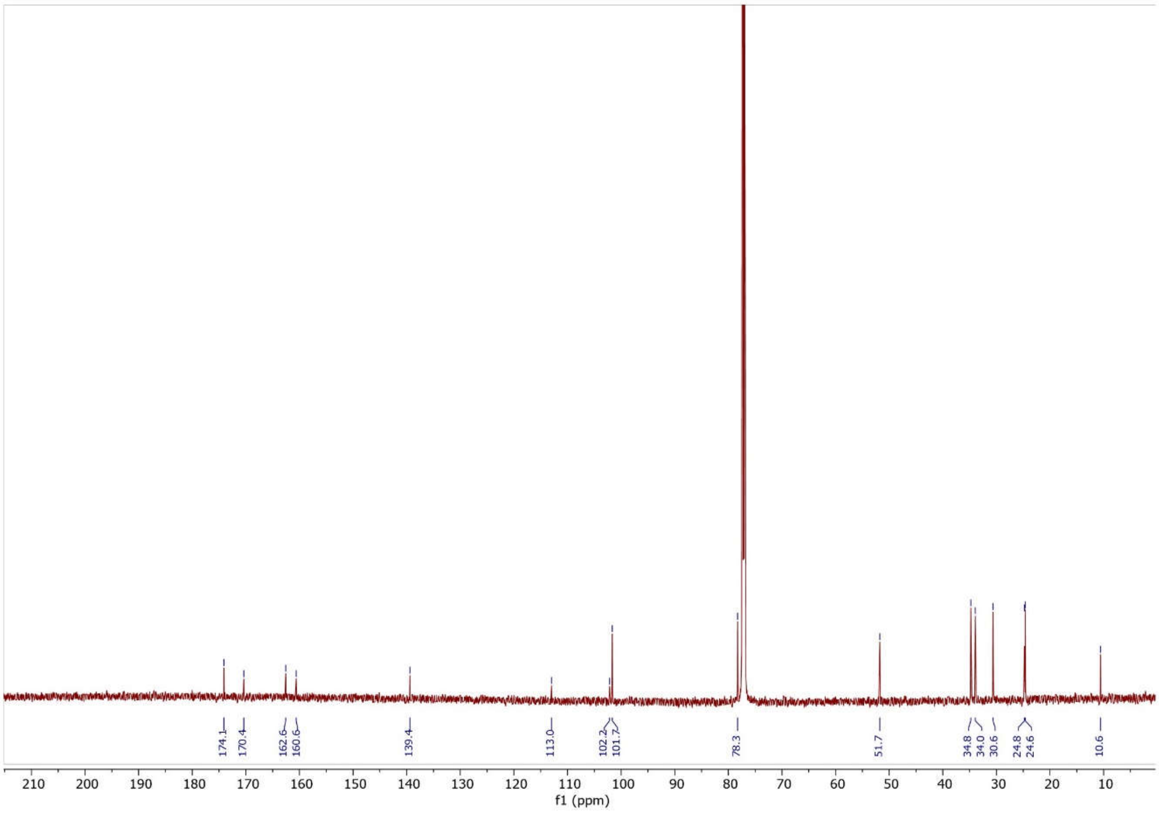

Figure S41. COSY spectrum of compound **5** ($CDCl_3$, 600 MHz)

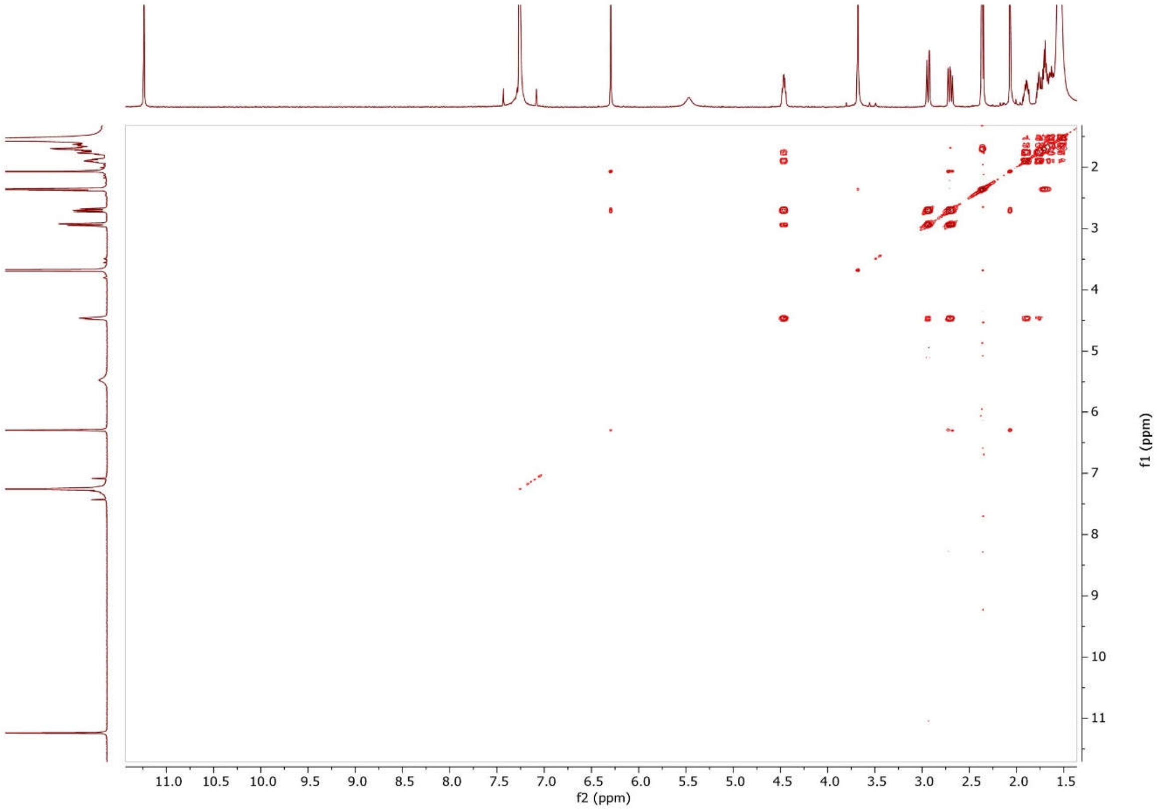

Figure S42. HSQC spectrum of compound **5** ($CDCl_3$, 600/150 MHz)

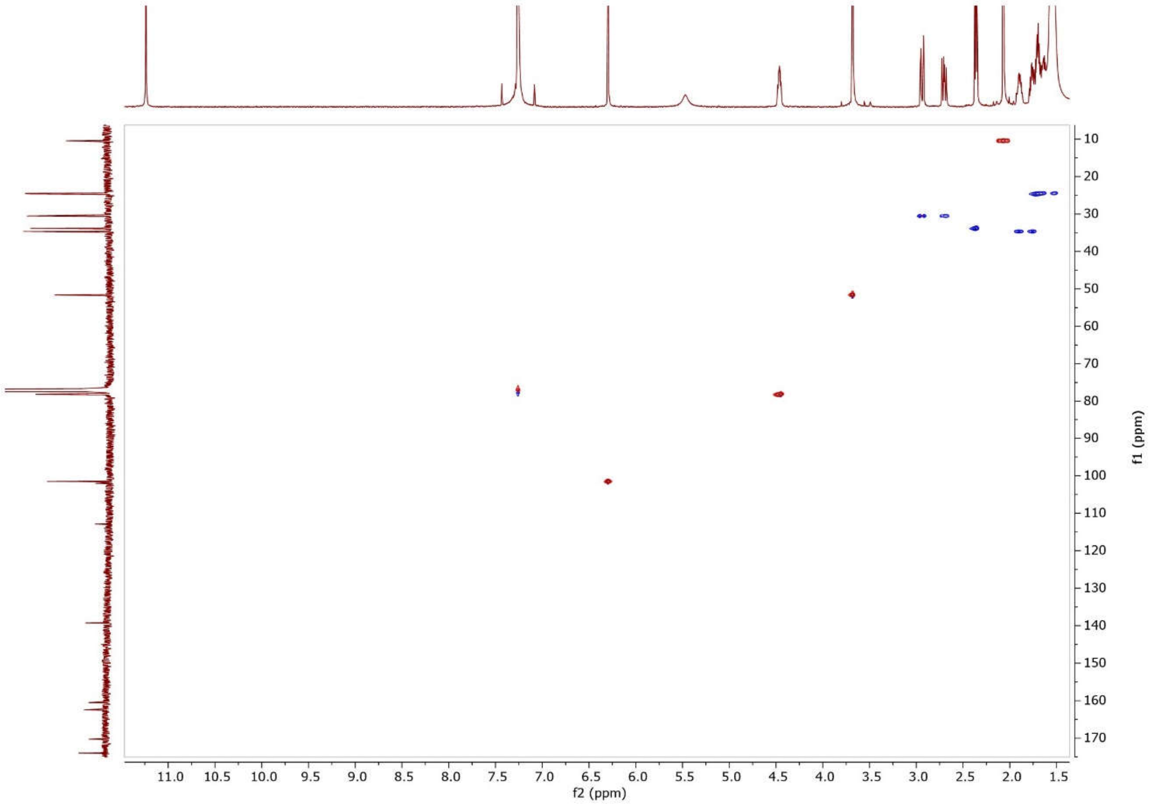

Figure S43. HMBC spectrum of compound **5** ($CDCl_3$, 600/150 MHz)

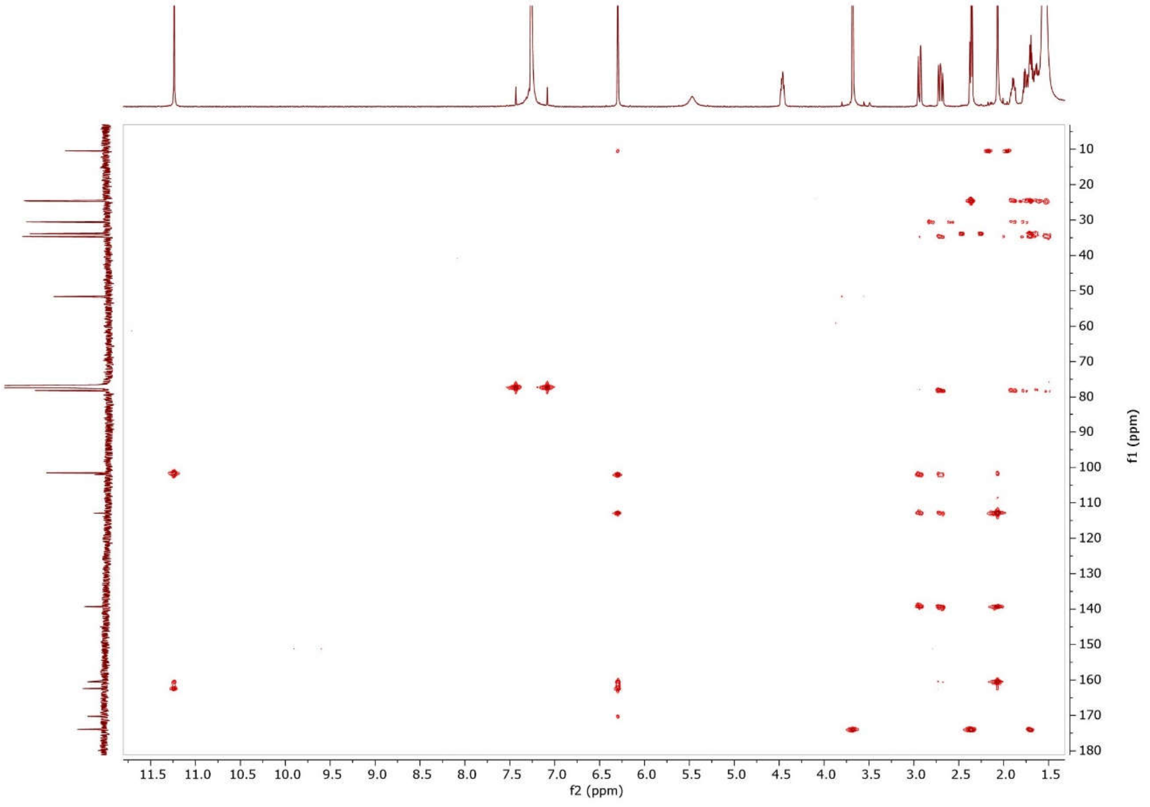

Figure S44. NOESY spectrum of compound **5** ($CDCl_3$, 600 MHz)

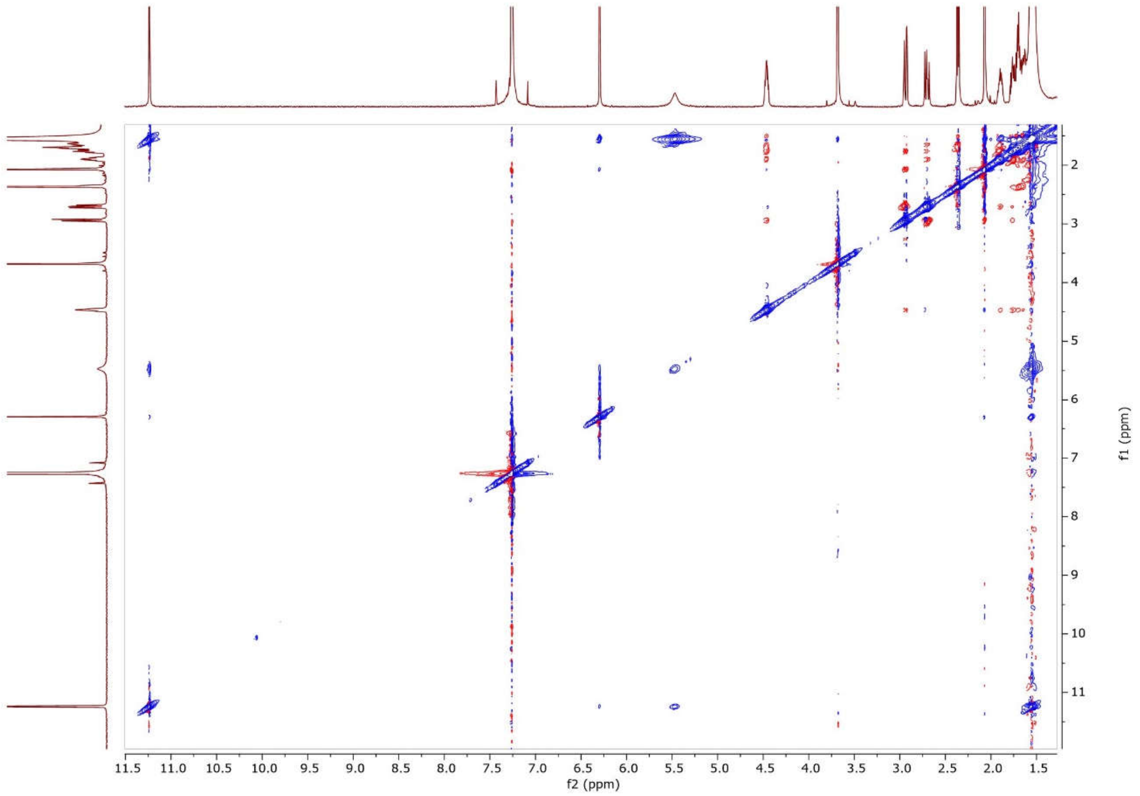

Figure S45. HR-ESIMS spectrum of compound **5**

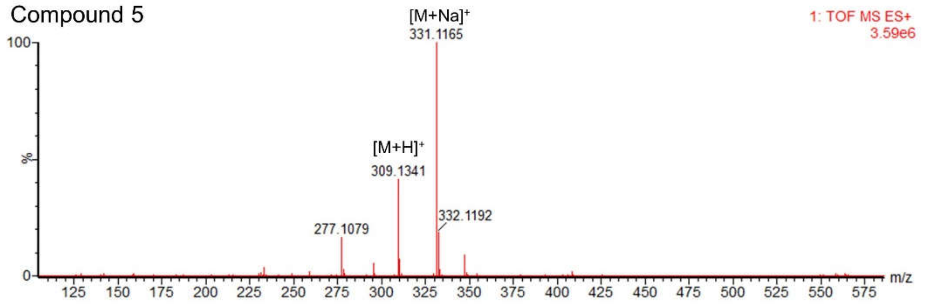

Figure S46. HR-ESIMS/MS spectrum of compound **5**

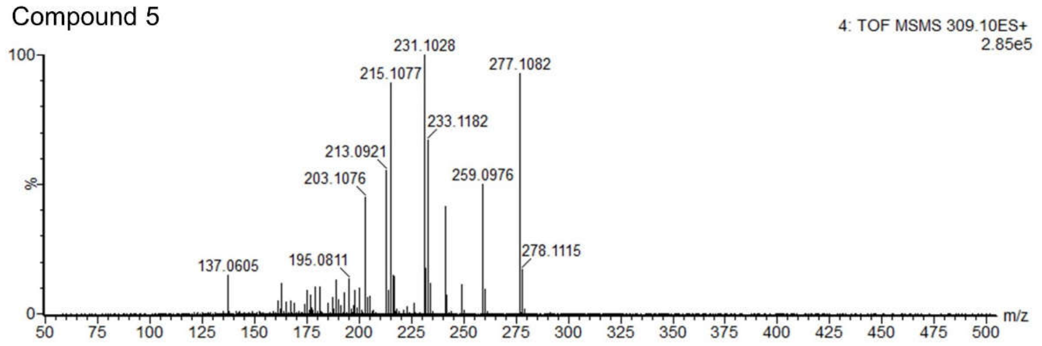

Figure S47. FT-IR spectrum of compound **5**

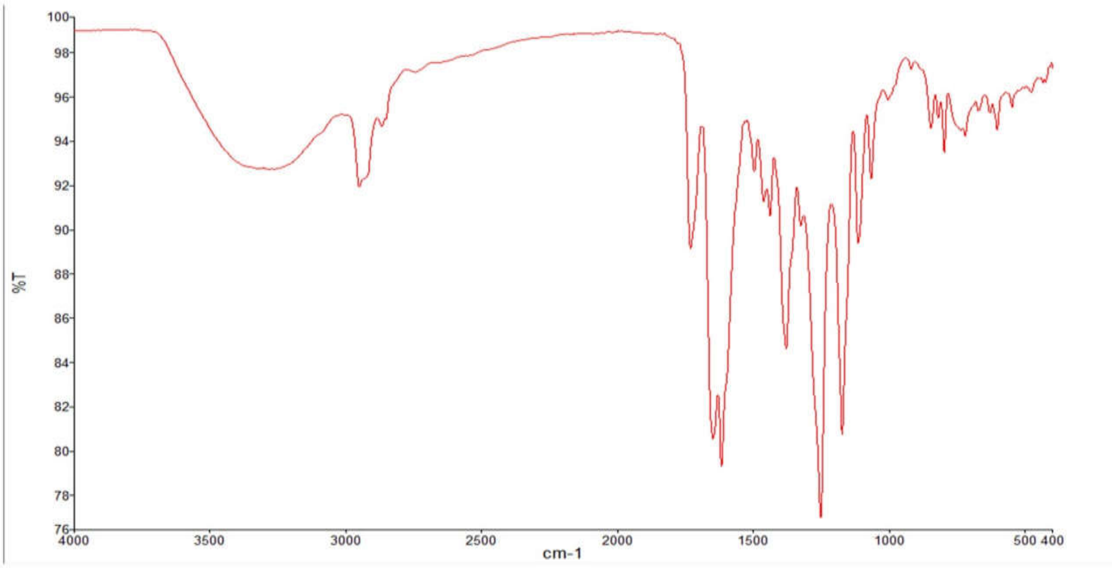

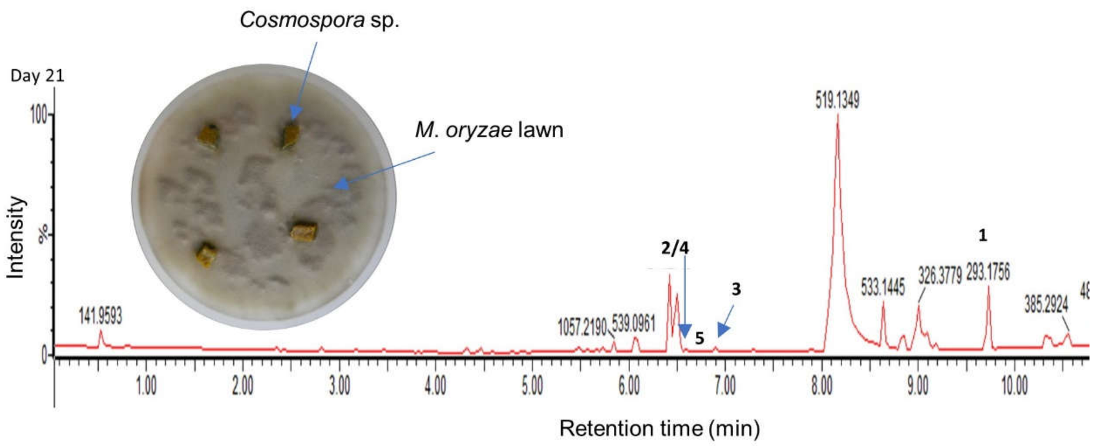

Figure S48. Agar plate (9 cm Petri dish) of overlaid co-cultivation of *Cosmospora* sp. on *M. oryzae* after 21 days. UPLC chromatogram of the extract shows the expression of compounds **1-5** with **2-4** in very low intensity.

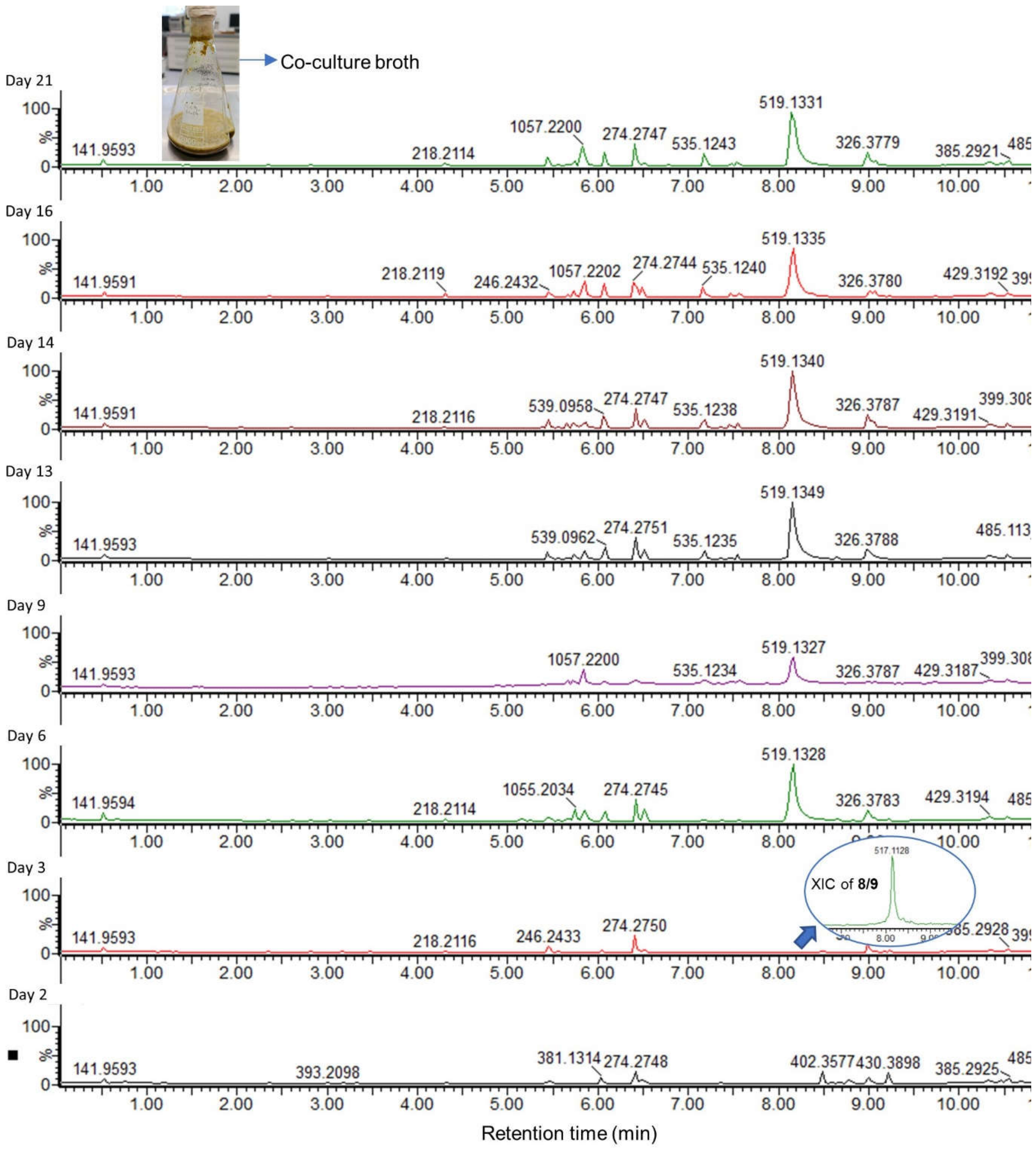

Figure S49. UPLC chromatograms of the extracts of co-cultures of *Cosmospora* sp. and *M. oryzae* in potato dextrose broth (PDB) at selected time points (Day 2, 3, 6, 9, 13, 14, 16 and 21). Extracted ion chromatogram (XIC) of compounds **8/9**, showing their biosynthesis from day 3 of co-cultivation in PDB medium.

Mosher's ester analysis

Compound **2** (0.2 mg) was treated with (trimethylsilyl)diazomethane to protect the phenolic OH groups prior to MPA derivatization, to give compound **2m** (Figure S50). The reaction product was split into two aliquots, which were treated separately with a ten-fold molar excess of (*R*)- and (*S*)-MPA acid (methoxyphenylacetic acid), respectively, in the presence of EDC (1-ethyl-3-(3-dimethylaminopropyl)carbodiimide) and DMAP (4-dimethylaminopyridine), to give the corresponding MPA esters (Figure S50).

LC-MS analysis of crude reaction mixtures showed that each reaction produced a mixture in the approximative 2:1 ratio of two isomers with *m*/*z* 485.2534, with the same retention times, but with exchanged intensities between the (*R*)-MPA and (*S*)-MPA reactions (Figure S51). Considering that enantiomeric compounds, such as (*R*)-MPA-(6'*R*)-**2m** and (*S*)-MPA-(6'*S*)-**2m** must show the same retention time on a non-chiral HPLC column, the isomers were determined as epimers at C-6'. 1D-TOCSY experiments were performed on the samples from the (*R*)- and (*S*)-MPA reactions (Figure S52). Only the chemical shift of the Me group at 7' could be determined, but this was sufficient to assign configuration. For the most abundant epimer, H_3-7' was deshielded in the (*R*)-MPA ester and shielded in the (*S*)-MPA esters. This indicated the 6'*R* configuration for the major epimer.

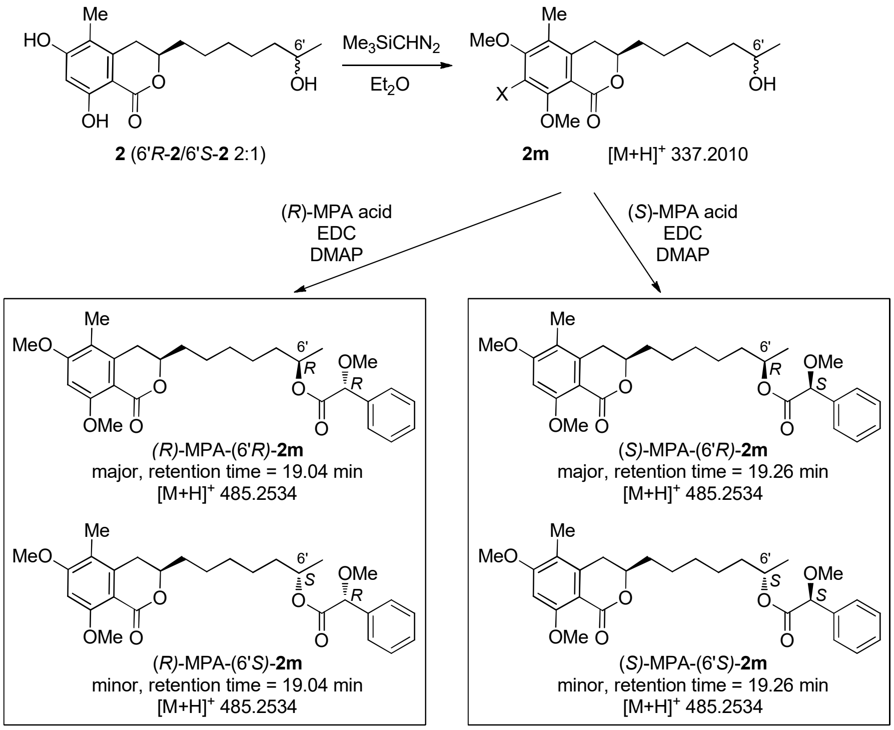

Figure S50: Mosher's ester analysis scheme for compound **2**

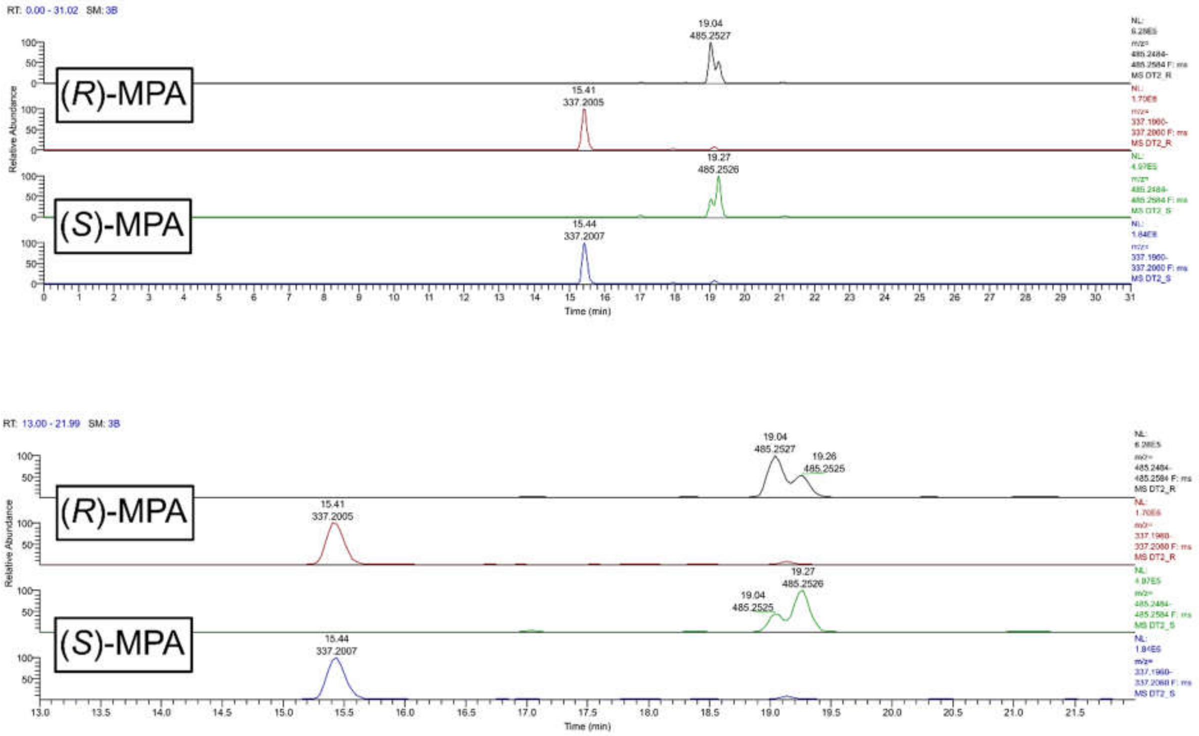

Figure S51: Full (top traces) and expanded (bottom traces) LC-MS chromatograms of the (*R*)- and (*S*)-MPA reaction mixtures from compound **2.** The extracted ion chromatograms (XIC) at *m/z* 485.2534 (MPA esters) of (*R*)-MPA reaction mixture is shown in black and that of (*S*)-MPA reaction mixture is shown in green. The XIC at *m/z* 337.2010 (unreacted **2m**) of (*R*)-MPA reaction mixture (red trace) and (*S*)-MPA reaction mixture (blue trace) are shown as reference for retention time reproducibility.

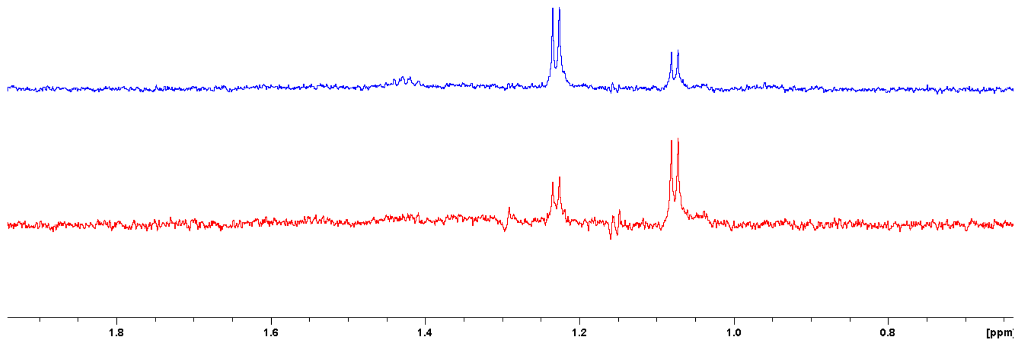

Figure S52: NMR analysis of the (*R*)- and (*S*)-MPA reaction mixtures from compound **2**. The 1D-TOCSY spectra of the two samples with excitation window δ 4.95-4.90 (the chemical shift of H-6' in the esters) are shown. Only the signals of the methyl groups at position 7' were clearly above the noise level. Blue top trace: (*R*)-MPA esters, red bottom trace: (*S*)-MPA esters.

Compound **4** (approximately 0.1 mg) was processed in the same way as described for compound **2**, but only the (*R*)-MPA ester was prepared due to the low amounts of the sample (Figures S53). In this case, LC-MS analysis showed a 1:1 mixture of two isomers at *m*/*z* 485.2534 (Figure S54), assigned as epimers at C-5' using the same reasoning as above. Because the LC-MS analysis revealed a 1:1 mixture, no NMR analysis was performed.

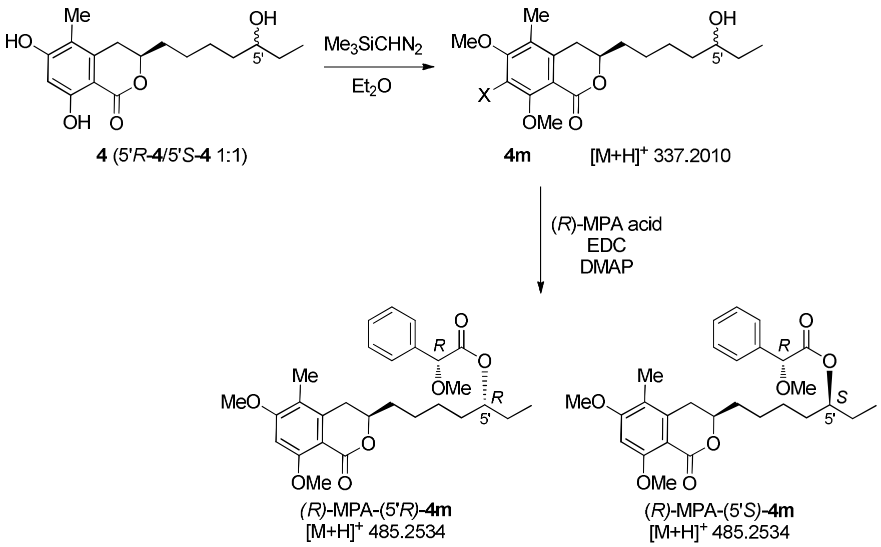

Figure S53: Mosher's ester analysis scheme for compound **4**

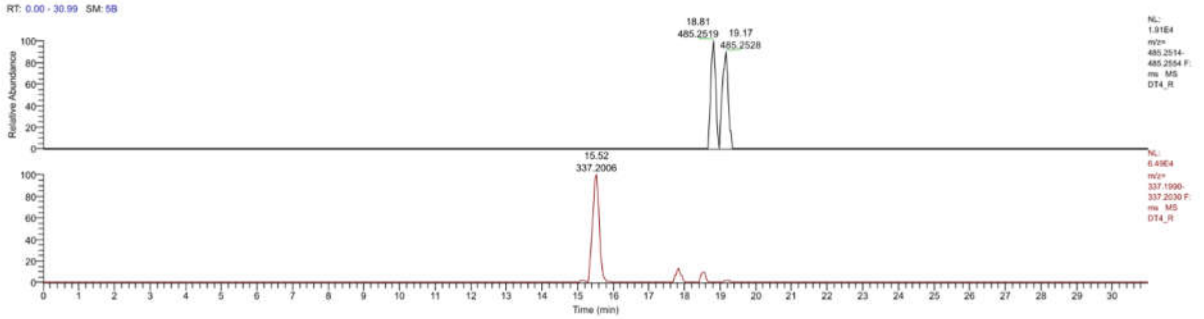

Figure S54: LC-MS chromatograms of the (*R*)-MPA reaction mixture from compound **4.** The extracted ion chromatograms (XIC) at *m*/*z* 485.2534 (MPA ester) is shown in black, the XIC at *m*/*z* 337.2010 (unreacted **4m**) is shown in red.

References

1. Sumner, L.W.; Amberg, A.; Barrett, D.; Beale, M.H.; Beger, R.; Daykin, C.A.; Fan, T.W.-M.; Fiehn, O.; Goodacre, R.; Griffin, J.L.; et al. Proposed minimum reporting standards for chemical analysis. *Metabolomics* **2007**, *3*, 211-221, doi:10.1007/s11306-007-0082-2.
2. Rusman, Y.; Held, B.W.; Blanchette, R.A.; Wittlin, S.; Salomon, C.E. Soudanones A-G: antifungal isochromanones from the Ascomycetous fungus *Cadophora* sp. isolated from an iron mine. *J. Nat. Prod.* **2015**, *78*, 1456-1460, doi:10.1021/acs.jnatprod.5b00204.
3. Kock, I.; Draeger, S.; Schulz, B.; Elsässer, B.; Kurtán, T.; Kenéz, Á.; Rheinheimer, J. Pseudoanguillosporin A and B: two new isochromans isolated from the endophytic fungus *Pseudoanguillospora* sp. *Eur. J. Org. Chem.* **2009**, *2009*, 1427-1434, doi:10.1002/ejoc.200801083.
4. Koyama, K.; Natori, S. Further characterization of seven bis (naphtho-γ-pyrone) congeners of ustilaginoidins, coloring matters of *Claviceps virens* (*Ustilaginoidea virens*). *Chem. Pharm. Bull.* **1988**, *36*, 146-152, doi:10.1248/cpb.36.146.
5. Wang, Y.; Xu, L.; Ren, W.; Zhao, D.; Zhu, Y.; Wu, X. Bioactive metabolites from *Chaetomium globosum* L18, an endophytic fungus in the medicinal plant *Curcuma wenyujin*. *Phytomedicine* **2012**, *19*, 364-368, doi:10.1016/j.phymed.2011.10.011.
6. Sun, W.; Wang, A.; Xu, D.; Wang, W.; Meng, J.; Dai, J.; Liu, Y.; Lai, D.; Zhou, L. New ustilaginoidins from rice false smut balls caused by *Villosiclava virens* and their phytotoxic and cytotoxic activities. *J. Agric. Food Chem.* **2017**, *65*, 5151-5160, doi:10.1021/acs.jafc.7b01791.
7. El-Elimat, T.; Figueroa, M.; Ehrmann, B.M.; Cech, N.B.; Pearce, C.J.; Oberlies, N.H. High-resolution MS, MS/MS, and UV database of fungal secondary metabolites as a dereplication protocol for bioactive natural products. *J. Nat. Prod.* **2013**, *76*, 1709-1716, doi:10.1021/np4004307.
8. Heald, S.L.; Jeffs, P.W.; Wheat, R.W. The identification of ergosterol and Δ9(11)-dehydroergosterol from mycelia of *Coccidioides immitis* by reverse-phase high-performance liquid and gas chromatography and ultraviolet and mass spectrometry. *Exp. Mycol.* **1981**, *5*, 162-166, doi:10.1016/0147-5975(81)90017-7.
9. Liu, H.; Zhao, X.; Guo, M.; Liu, H.; Zheng, Z. Growth and metabolism of *Beauveria bassiana* spores and mycelia. *BMC Microbiol.* **2015**, *15*, 1-12, doi:10.1186/s12866-015-0592-4.
